# Risk thresholds for alcohol consumption: combined analysis of individual-participant data for 599 912 current drinkers in 83 prospective studies

**DOI:** 10.1016/S0140-6736(18)30134-X

**Published:** 2018-04-14

**Authors:** Angela M Wood, Stephen Kaptoge, Adam S Butterworth, Peter Willeit, Samantha Warnakula, Thomas Bolton, Ellie Paige, Dirk S Paul, Michael Sweeting, Stephen Burgess, Steven Bell, William Astle, David Stevens, Albert Koulman, Randi M Selmer, W M Monique Verschuren, Shinichi Sato, Inger Njølstad, Mark Woodward, Veikko Salomaa, Børge G Nordestgaard, Bu B Yeap, Astrid Fletcher, Olle Melander, Lewis H Kuller, Beverley Balkau, Michael Marmot, Wolfgang Koenig, Edoardo Casiglia, Cyrus Cooper, Volker Arndt, Oscar H Franco, Patrik Wennberg, John Gallacher, Agustín Gómez de la Cámara, Henry Völzke, Christina C Dahm, Caroline E Dale, Manuela M Bergmann, Carlos J Crespo, Yvonne T van der Schouw, Rudolf Kaaks, Leon A Simons, Pagona Lagiou, Josje D Schoufour, Jolanda M A Boer, Timothy J Key, Beatriz Rodriguez, Conchi Moreno-Iribas, Karina W Davidson, James O Taylor, Carlotta Sacerdote, Robert B Wallace, J Ramon Quiros, Rosario Tumino, Dan G Blazer, Allan Linneberg, Makoto Daimon, Salvatore Panico, Barbara Howard, Guri Skeie, Timo Strandberg, Elisabete Weiderpass, Paul J Nietert, Bruce M Psaty, Daan Kromhout, Elena Salamanca-Fernandez, Stefan Kiechl, Harlan M Krumholz, Sara Grioni, Domenico Palli, José M Huerta, Jackie Price, Johan Sundström, Larraitz Arriola, Hisatomi Arima, Ruth C Travis, Demosthenes B Panagiotakos, Anna Karakatsani, Antonia Trichopoulou, Tilman Kühn, Diederick E Grobbee, Elizabeth Barrett-Connor, Natasja van Schoor, Heiner Boeing, Kim Overvad, Jussi Kauhanen, Nick Wareham, Claudia Langenberg, Nita Forouhi, Maria Wennberg, Jean-Pierre Després, Mary Cushman, Jackie A Cooper, Carlos J Rodriguez, Masaru Sakurai, Jonathan E Shaw, Matthew Knuiman, Trudy Voortman, Christa Meisinger, Anne Tjønneland, Hermann Brenner, Luigi Palmieri, Jean Dallongeville, Eric J Brunner, Gerd Assmann, Maurizio Trevisan, Richard F Gillum, Ian Ford, Naveed Sattar, Mariana Lazo, Simon G Thompson, Pietro Ferrari, David A Leon, George Davey Smith, Richard Peto, Rod Jackson, Emily Banks, Emanuele Di Angelantonio, John Danesh, Emerging Risk Factors Collaboration/EPIC-CVD/UK Biobank Alcohol Study Group, Angela M Wood, Stephen Kaptoge, Adam Butterworth, Peter Willeit, Samantha Warnakula, Thomas Bolton, Ellie Paige, Dirk S Paul, Michael Sweeting, Stephen Burgess, Steven Bell, William Astle, David Stevens, Albert Koulman, Randi M Selmer, Monique Verschuren, Shinichi Sato, Inger Njølstad, Mark Woodward, Salomaa Veikko, Børge G Nordestgaard, Bu B Yeap, Astrid Flecther, Olle Melander, Lewis H Kuller, Beverley Balkau, Michael Marmot, Wolfgang Koenig, Edoardo Casiglia, Cyrus Cooper, Volker Arndt, Oscar H Franco, Patrik Wennberg, John Gallacher, Agustín Gómez de la Cámara, Henry Völzke, Christina C Dahm, Caroline E Dale, Manuela Bergmann, Carlos Crespo, Yvonne T van der Schouw, Rudolf Kaaks, Leon A Simons, Pagona Lagiou, Josje D Schoufour, Jolanda M.A Boer, Timothy J Key, Beatriz Rodriguez, Conchi Moreno-Iribas, Karina W Davidson, James O Taylor, Carlotta Sacerdote, Robert B Wallace, J. Ramon Quiros, Eric B Rimm, Rosario Tumino, Dan G Blazer III, Allan Linneberg, Makoto Daimon, Salvatore Panico, Barbara Howard, Guri Skeie, Veikko Salomaa, Timo Strandberg, Elisabete Weiderpass, Paul J Nietert, Bruce M Psaty, Daan Kromhout, Elena Salamanca-Fernandez, Stefan Kiechl, Harlan M Krumholz, Sara Grioni, Domenico Palli, José M Huerta, Jackie Price, Johan Sundström, Larraitz Arriola, Hisatomi Arima, Ruth C Travis, Demosthenes B Panagiotakos, Anna Karakatsani, Antonia Trichopoulou, Tilman Kühn, Diederick E Grobbee, Elizabeth Barrett-Connor, Natasja van Schoor, Heiner Boeing, Kim Overvad, Jussi Kauhanen, Nick Wareham, Claudia Langenberg, Nita Forouhi, Maria Wennberg, Jean-Pierre Després, Mary Cushman, Jackie A Cooper, Carlos J Rodriguez, Masaru Sakurai, Jonathan E Shaw, Matthew Knuiman, Trudy Voortman, Christa Meisinger, Anne Tjønneland, Hermann Brenner, Luigi Palmieri, Jean-Pierre Dallongeville, Eric J Brunner, Gerd Assmann, Maurizio Trevisan, Richard F Gillumn, Ian Ford Ford, Naveed Sattar, Mariana Lazo, Simon Thompson, Pietro Ferrari, David A Leon, George Davey Smith, Richard Peto, Rod Jackson, Emily Banks, Emanuele Di Angelantonio, John Danesh

**Affiliations:** a Department of Public Health and Primary Care, University of Cambridge, Cambridge, UK; b Medical University Innsbruck, Innsbruck, Austria; c National Centre for Epidemiology and Population Health, Australian National University, Canberra, Australia; d MRC Biostatistics Unit, Cambridge Institute of Public Health, University of Cambridge, Cambridge, UK; e NIHR BRC Nutritional Biomarker Laboratory, University of Cambridge, Cambridge, UK; f Norwegian Institute of Public Health, Oslo, Norway; g National Institute for Public Health and the Environment, Bilthoven, Netherlands; h Julius Centre for Health Sciences and Primary Care, University Medical Center Utrecht, Utrecht, Netherlands; i Chiba Prefectural Institute of Public Health, Chiba, Japan; j Department of Community Medicine, University of Tromsø, Tromsø, Norway; k Nuffield Department of Population Health, Medical Sciences Division, University of Oxford, Oxford, UK; l The George Institute for Global Health, University of Sydney, Sydney, NSW, Australia; m Bloomberg School of Public Health, Johns Hopkins University, Baltimore, MD, USA; n School of Medicine, Johns Hopkins University, Baltimore, MD, USA; o THL-National Institute for Health and Welfare, Helsinki, Finland; p Copenhagen University Hospital, Copenhagen, Denmark; q Department of Clinical Medicine, University of Copenhagen, Copenhagen, Denmark; r School of Medicine, University of Western Australia, Perth, WA, Australia; s Fiona Stanley Hospital, Perth, WA, Australia; t Harry Perkins Institute of Medical Research, Perth, WA, Australia; u London School of Hygiene & Tropical Medicine, London, UK; v Department of Clinical Sciences, Malmö, Lund University, Malmö, Sweden; w Graduate School of Public Health, University of Pittsburgh, Pittsburgh, PA, USA; x CESP INSERM UMRS 1018, Villejuif Cedex, France; y Department of Epidemiology and Public Health, University College London, London, UK; z 92 Deutsches Herzzentrum München, Technische Universität München, Munich, Germany, DZHK (German Centre for Cardiovascular Research), partner site Munich Heart Alliance, Munich, Germany; aa University of Ulm Medical Center, Ulm, Germany; ab Department of Medicine, University of Padua, Padua, Italy; ac MRC Lifecourse Epidemiology Unit, University of Southampton, Southampton, UK; ad German Cancer Research Center (DKFZ), Heidelberg, Germany; ae Erasmus University Medical Center Rotterdam, Rotterdam, Netherlands; af Department of Public Health and Clinical Medicine, Umeå University, Umeå, Sweden; ag Department of Primary Care and Public Health, Cardiff University, Cardiff, UK; ah 12 de Octubre Research Institute, CIBERESP, Madrid, Spain; ai Institute for Community Medicine, University Medicine Greifswald, Greifswald, Germany; aj Department of Public Health, Aarhus University, Aarhus, Denmark; ak Farr Institute of Health Informatics Research, UCL Institute of Health Informatics, University College London, London, UK; al German Institute of Human Nutrition, Potsdam–Rehbrüke, Germany; am School of Community Health, Portland State University, Portland, OR, USA; an St Vincent's Clinical School, University of New South Wales, Sydney, NSW, Australia; ao Hellenic Health Foundation, Athens, Greece; ap National and Kapodistrian University of Athens, Athens, Greece; aq Harvard TH Chan School of Public Health, Boston, MA, USA; ar Office of Public Health Studies, University of Hawaii, Honolulu, HI, USA; as Instituto de Salud Pública de Navarra, IdiSNA - Navarra Institute for Health Research, Pamplona, Spain; at Red de Investigación en Servicios de Salud en Enfermedades Crónicas (REDISSEC), Pamplona, Spain; au Columbia University Irving Medical Center, New York, NY, USA; av East Boston Neighborhood Health Center, Boston, MA, USA; aw Città della Salute e della Scienza di Torino Hospital, Turin, Italy; ax College of Public Health, The University of Iowa, Iowa City, IA, USA; ay Consejería de Sanidad del Principado de Asturias, Oviedo, Asturias, Spain; az Civic - M. Arezzo Hospital, ASP Ragusa, Italy; ba Duke Divinity School, Duke University, Durham, NC, USA; bb Department of Endocrinology and Metabolism, Hirosaki University, Hirosaki, Japan; bc Department of Clinical and Experimental Medicine, Federico II University, Naples, Italy; bd Department of Biology, Tuskegee University, AL, USA; be Department of Community Medicine, UiT The Arctic University of Norway, Tromsø, Norway; bf University of Helsinki and Helsinki University Hospital, Helsinki, Finland; bg Center for Life Course Health Research, University of Oulu, Oulu, Finland; bh Cancer Registry of Norway, Institute of Population-Based Cancer Research, Oslo, Norway; bi Department of Medical Epidemiology and Biostatistics, Karolinska Institutet, Stockholm, Sweden; bj Genetic Epidemiology Group, Folkhälsan Research Center, Faculty of Medicine, University of Helsinki, Helsinki, Finland; bk Medical University of South Carolina, Charleston, SC, USA; bl Cardiovascular Health Research Unit, Departments of Medicine, Epidemiology, and Health Services, University of Washington, Seattle, WA, USA; bm Kaiser Permanente Washington Health Research Institute, Seattle, WA, USA; bn Department of Agrotechnology and Food Sciences, University of Wageningen, Wageningen, Netherlands; bo Faculty of Medical Sciences, University of Groningen, Groningen, Netherlands; bp Escuela Andaluza de Salud Pública, Instituto de Investigación Biosanitaria ibs.GRANADA, Hospitales Universitarios de Granada/Universidad de Granada, Granada, Spain; bq CIBER de Epidemiología y Salud Pública (CIBERESP), Madrid, Spain; br School of Medicine, Yale University, New Haven, CT, USA; bs Epidemiology and Prevention Unit, Fondazione IRCCS Istituto Nazionale dei Tumori, Milan, Italy; bt Cancer Research and Prevention Institute (ISPO), Florence, Italy; bu Murcia Regional Health Council, IMIB-Arrixaca, Murcia, Spain; bv Usher Institute, University of Edinburgh, Edinburgh, UK; bw Department of Medical Sciences, Uppsala University, Uppsala, Sweden; bx Instituto BIO-Donostia, Basque Government, San Sebastian, Spain; by The University of Sydney and Royal Prince Alfred Hospital, Sydney, NSW, Australia; bz Department of Preventive Medicine and Public Health, Kyushu University, Fukuoka, Japan; ca School of Health Science and Education, Harokopio University, Athens, Greece; cb Department of Family Medicine and Public Health, University of California, San Diego, CA, USA; cc EMGO Institute for Health and Care Research, VU University Medical Center, Amsterdam, Netherlands; cd Aalborg University Hospital, Aalborg, Denmark; ce Institute of Public Health and Clinical Nutrition, University of Eastern Finland, Kuopio, Finland; cf Medical Research Council Epidemiology Unit, University of Cambridge, Cambridge, UK; cg Department of Kinesiology, Laval University, Quebec City, QC, Canada; ch Department of Medicine, University of Vermont, Burlington, VT, USA; ci Wake Forest University School of Medicine, Winston-Salem, NC, USA; cj Wake Forest Baptist Medical Center, Winston-Salem, NC, USA; ck Department of Social and Environmental Medicine, Kanazawa Medical University, Ishikawa, Japan; cl Baker IDI Heart and Diabetes Institute, Melbourne, VIC, Australia; cm Busselton Population Medical Research Institute, Busselton, WA, Australia; cn School of Population and Global Health, The University of Western Australia, Perth, WA, Australia; co Helmholtz Zentrum München German Research Center for Environmental Health, Germany; cp Danish Cancer Society Research Center, Copenhagen, Denmark; cq Division of Clinical Epidemiology and Aging Research, University of Heidelberg, Heidelberg, Germany; cr Istituto Superiore di Sanità, Rome, Italy; cs Institut Pasteur de Lille, Lille, France; ct Assmann-Stiftung für Prävention, Münster, Germany; cu The City College of New York, New York, NY, USA; cv Howard University Hospital, Washington DC, USA; cw Institute of Cardiovascular & Medical Sciences, University of Glasgow, Glasgow, UK; cx International Agency for Research on Cancer, Lyon, France; cy MRC Integrative Epidemiology Unit (IEU), University of Bristol, Bristol, UK; cz School of Population Health, The University of Auckland, Auckland, New Zealand

## Abstract

**Background:**

Low-risk limits recommended for alcohol consumption vary substantially across different national guidelines. To define thresholds associated with lowest risk for all-cause mortality and cardiovascular disease, we studied individual-participant data from 599 912 current drinkers without previous cardiovascular disease.

**Methods:**

We did a combined analysis of individual-participant data from three large-scale data sources in 19 high-income countries (the Emerging Risk Factors Collaboration, EPIC-CVD, and the UK Biobank). We characterised dose–response associations and calculated hazard ratios (HRs) per 100 g per week of alcohol (12·5 units per week) across 83 prospective studies, adjusting at least for study or centre, age, sex, smoking, and diabetes. To be eligible for the analysis, participants had to have information recorded about their alcohol consumption amount and status (ie, non-drinker *vs* current drinker), plus age, sex, history of diabetes and smoking status, at least 1 year of follow-up after baseline, and no baseline history of cardiovascular disease. The main analyses focused on current drinkers, whose baseline alcohol consumption was categorised into eight predefined groups according to the amount in grams consumed per week. We assessed alcohol consumption in relation to all-cause mortality, total cardiovascular disease, and several cardiovascular disease subtypes. We corrected HRs for estimated long-term variability in alcohol consumption using 152 640 serial alcohol assessments obtained some years apart (median interval 5·6 years [5th–95th percentile 1·04–13·5]) from 71 011 participants from 37 studies.

**Findings:**

In the 599 912 current drinkers included in the analysis, we recorded 40 310 deaths and 39 018 incident cardiovascular disease events during 5·4 million person-years of follow-up. For all-cause mortality, we recorded a positive and curvilinear association with the level of alcohol consumption, with the minimum mortality risk around or below 100 g per week. Alcohol consumption was roughly linearly associated with a higher risk of stroke (HR per 100 g per week higher consumption 1·14, 95% CI, 1·10–1·17), coronary disease excluding myocardial infarction (1·06, 1·00–1·11), heart failure (1·09, 1·03–1·15), fatal hypertensive disease (1·24, 1·15–1·33); and fatal aortic aneurysm (1·15, 1·03–1·28). By contrast, increased alcohol consumption was log-linearly associated with a lower risk of myocardial infarction (HR 0·94, 0·91–0·97). In comparison to those who reported drinking >0–≤100 g per week, those who reported drinking >100–≤200 g per week, >200–≤350 g per week, or >350 g per week had lower life expectancy at age 40 years of approximately 6 months, 1–2 years, or 4–5 years, respectively.

**Interpretation:**

In current drinkers of alcohol in high-income countries, the threshold for lowest risk of all-cause mortality was about 100 g/week. For cardiovascular disease subtypes other than myocardial infarction, there were no clear risk thresholds below which lower alcohol consumption stopped being associated with lower disease risk. These data support limits for alcohol consumption that are lower than those recommended in most current guidelines.

**Funding:**

UK Medical Research Council, British Heart Foundation, National Institute for Health Research, European Union Framework 7, and European Research Council.

## Introduction

Alcohol consumption guidelines vary substantially across the globe.^1^, ^2^ In the USA, for example, an upper limit of 196 g per week (about 11 standard UK glasses of wine or pints of beer per week) is recommended for men, and an upper limit of 98 g per week is recommended for women.^1^ Similar recommendations apply in Canada and Sweden.^2^ By contrast, guidelines in Italy, Portugal, and Spain recommend low-risk limits almost 50% higher than these.^1^, ^2^ At the other extreme, UK guidelines recommend low-risk limits for men almost half that recommended by US guidelines.^1^, ^2^

Such variation in policy might reflect ambiguity about drinking risk thresholds associated with the lowest risk of mortality,^3^, ^4^, ^5^, ^6^, ^7^, ^8^, ^9^, ^10^, ^11^, ^12^, ^13^, ^14^, ^15^ as well as uncertainty about the specific consequences of alcohol consumption, including those related to cardiovascular disease subtypes. For example, recent studies have challenged the concept that moderate alcohol consumption is universally associated with lower cardiovascular disease risk,^16^, ^17^ but the dose–response associations of alcohol consumption with cardiovascular disease subtypes remain poorly understood. Therefore, to help in the formulation of evidence-based alcohol policy, we analysed individual-participant data from 83 long-term prospective studies in 19 high-income countries. Our aim was to characterise risk thresholds for all-cause mortality and cardiovascular disease subtypes in current drinkers of alcohol.

## Methods

### Study design, data sources, and participants

We focused our study on current alcohol drinkers for three main reasons. First, alcohol guidelines provide recommendations about low-risk limits only for drinkers (we are unaware of any guidelines that encourage non-drinkers to consume alcohol). Second, a focus on current drinkers should limit potential biases that are difficult to control in observational studies (eg, reverse causality, residual confounding, and unmeasured effect modification) because ex-drinkers include people who might have abstained from alcohol owing to poor health itself,^18^, ^19^, ^20^ as well as those who have changed their habits to achieve a healthier lifestyle. Third, never-drinkers might differ systematically from drinkers in ways that are difficult to measure, but which might be relevant to disease causation.^21^

We did a combined analysis of individual-participant data from three large-scale data sources available to our consortium, each constituting purpose-designed prospective cohort studies with quantitative information about alcohol consumption (appendix p 21). First, the Emerging Risk Factors Collaboration (ERFC) is a collaboration of prospective cohort studies with information about a variety of risk factors, cardiovascular disease outcomes, and mortality.^22^ Of the 102 studies in the ERFC with information about alcohol status, 81 contained information about the quantity of consumption. Second, EPIC-CVD, a ten-country case-cohort study nested in the European Prospective Investigation into Cancer and Nutrition (EPIC) prospective cohort study, had quantitative alcohol information from 22 of its 23 contributing centres.^23^ Third, UK Biobank—a single large prospective study—had cohort-wide data about quantitative alcohol consumption.^24^ Therefore, our combined analysis included information from a total of 83 prospective studies that each used broadly similar methods to quantify alcohol consumption, record risk factors, and ascertain cause-specific death and cardiovascular disease events. We harmonised records of alcohol consumption across the contributing studies using a conversion of 1 unit=8 g of pure alcohol to a standard scale of grams per week (appendix pp 1–2), enabling a common analytical approach despite variation in the methods used (eg, self-administered *vs* interview-led questionnaires; food frequency questionnaires *vs* dietary recall surveys), and in consumption scales over different periods of ascertainment. Details of contributing studies are in appendix pp 3–4, 10–11.

**Research in context**

**Evidence before this study**

We searched for prospective epidemiological studies of alcohol consumption investigating disease risk thresholds published in any language up until March 1, 2017 (with no specified earliest date), in PubMed, Scientific Citation Index Expanded, and Embase using relevant terms (“alcohol”, “mortality”, “survival”, “cardiovascular disease”, “cohort”, and “prospective”). We found many primary reports and literature-based reviews. However, no study had combined the following key features required to achieve reliable estimates of dose–response associations: availability of individual-participant data; quantitative assessment of alcohol consumption levels using validated instruments; periodic re-surveys of alcohol consumption levels; recording of large numbers of deaths (eg, >20 000 deaths); and sufficient detail and power to disaggregate incident cardiovascular disease outcomes into subtypes (eg, >20 000 incident total cardiovascular disease outcomes).

**Added value of this study**

The current study combined all the key study design features mentioned above, and afforded several additional advantages. First, it reduced the potentially distorting effects of reverse causality by focusing on current drinkers without previous cardiovascular disease who survived at least 12 months of follow-up. Second, it enhanced generalisability by including individual-participant data from 83 prospective studies in 19 different high-income countries. Third, it used a variety of established and emerging risk factors, enabling investigation of potential confounders and mediators.

**Implications of all the available evidence**

The chief implication of this study for public policy is to support reductions of alcohol consumption limits in existing guidelines, suggesting that the threshold for lowest risk for all-cause mortality is about 100 g per week (about 5–6 standard UK glasses of wine or pints of beer per week). The chief implication for scientific understanding is the strengthening of evidence that the association between alcohol consumption and total cardiovascular disease risk is actually comprised of several distinct and opposite dose–response curves rather than a single J-shaped association.

To be eligible for the analysis, participants had to have information recorded about their alcohol consumption amount and status (ie, non-drinker *vs* current drinker), plus age, sex, history of diabetes and smoking status, at least 1 year of follow-up after baseline, and no known baseline history of cardiovascular disease (defined as coronary heart disease, other heart disease, stroke, transient ischaemic attack, peripheral arterial disease, or cardiovascular surgery); appendix p 21. The main analyses focused on current drinkers, whose baseline alcohol consumption was categorised into eight predefined groups according to the amount in grams consumed per week: >0–≤25, >25–≤50, >50–≤75, >75–≤100, >100–≤150, >150–≤250, >250–≤350, and >350 g per week. We assessed alcohol consumption in relation to all-cause mortality, total cardiovascular disease, and the following cardiovascular disease subtypes (defined in appendix p 5): fatal and non-fatal myocardial infarction; fatal and non-fatal coronary disease excluding myocardial infarction; fatal and non-fatal stroke (including ischaemic, haemorrhagic, subarachnoid, and unclassified subtypes of stroke); fatal and non-fatal heart failure; and mortality from other cardiovascular causes, including cardiac dysrhythmia, hypertensive disease, sudden death, and aortic aneurysm.^7^, ^17^, ^25^ In analyses of cardiovascular disease subtypes, participants contributed follow-up time until the first outcome recorded (ie, cardiovascular deaths preceded by non-fatal outcomes were not included). Event times were censored at the end of follow-up or death from non-cardiovascular causes.

### Statistical analysis

Hazard ratios (HRs) for alcohol consumption were calculated separately within each study using Cox regression models, stratified by sex and with adjustment for known confounders: age, smoking status (current *vs* non-current) and history of diabetes. To account for EPIC-CVD's case-cohort design (which was used because lipids and other cardiovascular disease biomarkers were measured only in the case-cohort subset and not the full EPIC cohort), the Cox models for cardiovascular disease events were adapted using Prentice weights and stratified by centre.^26^ For the four case-control studies nested within prospective cohorts of the ERFC, odds ratios were calculated using, as appropriate, conditional or unconditional logistic regression models, taking into account relevant matching factors. Study-specific estimates were then pooled across studies by random-effects meta-analysis.^27^ We tested for violation of the proportional hazards assumption by including time interactions with alcohol consumption. To avoid model overfitting, studies with fewer than five incident cases of a particular outcome were excluded from analyses of that particular outcome.

To correct for measurement error and within-person variability in alcohol consumption over time, we estimated long-term average (henceforth, “usual”) alcohol consumption using multi-level regression calibration and information from 152 640 serial assessments in 71 011 individuals from 37 studies. This calculation was achieved either by regressing re-survey measurements (for the repeat alcohol assessments available in the ERFC studies and UK Biobank) or lifetime alcohol consumption measurements (for calculated lifetime alcohol consumption measurements available in EPIC-CVD) on baseline alcohol consumption, adjusted for duration of follow-up and baseline age, sex, smoking status, history of diabetes, other relevant covariate(s), and with random effects for study and re-survey.^28^, ^29^ The regression dilution ratio (ie, the calibration slope), which measures the extent of within-person variability,^28^ was extracted from the calibration model. HRs in this paper relate to usual alcohol consumption levels unless specified otherwise.

We assessed the shapes of associations for all-cause mortality and cardiovascular disease outcomes by calculating study-specific HRs within the predefined groups of baseline alcohol consumption, pooled them by multivariate random-effects meta-analysis, and plotted them against mean usual (and baseline) alcohol consumption within each group. We estimated 95% CIs for each group (including the reference group) that corresponded to the amount of information underlying each group.^30^, ^31^ For each major outcome, we determined the best fitting first or second order fractional polynomial^32^ to describe the association with baseline alcohol consumption (using a 1% significance level as evidence for a second order fractional polynomial over a first order fractional polynomial) using Cox regression models stratified by sex, study, and centre. Further analyses assumed a linear association with alcohol consumption, expressing results per 100 g per week (12·5 units/week) in usual alcohol consumption. To assess the effect of excluding known current drinkers with missing alcohol consumption data, we did a sensitivity analysis using multiple imputation within studies, before combining the data in a meta-analysis. We investigated associations with alcohol type (wine, beer, and spirits), consumption frequency (dichotomised as drinkers who consumed alcohol on ≤2 days per week or those who consumed alcohol on >2 days per week) and episodic heavy drinking (dichotomised as binge drinkers who consumed ≥100 g per drinking occasion or non-binge drinkers who consumed <100 g per drinking occasion).

We used regression calibration methods similar to those described above to estimate and adjust for long-term levels of potential confounding factors or mediators in individuals with available information. HRs were adjusted for usual levels of available potential confounders or mediators, including body-mass index (BMI), systolic blood pressure, high-density-lipoprotein cholesterol (HDL-C), low-density-lipoprotein cholesterol (LDL-C), total cholesterol, fibrinogen, and baseline measures for smoking amount (in pack-years), level of education reached (no schooling or primary education only *vs* secondary education *vs* university), occupation (not working *vs* manual *vs* office *vs* other), self-reported physical activity level (inactive *vs* moderately inactive *vs* moderately active *vs* active), self-reported general health (scaled 0–1 where low scores indicate poorer health), self-reported red meat consumption, and self-reported use of anti-hypertensive drugs. We investigated effect modification with formal tests for interaction, using a 0·1% significance threshold to make some allowance for multiple testing. Heterogeneity was investigated by grouping studies according to recorded characteristics and through meta-regression, assessed by the *I*^2^ statistic.^33^ Evidence of small study effects was assessed visually with funnel plots and by Begg and Mazumdar's test^34^ and Egger's test.^35^

Methods we used to estimate reductions in life expectancy (years of life lost) are described in the appendix (pp 6–7). Briefly, estimates of cumulative survival from 40 years of age onwards in different categories of baseline alcohol consumption were calculated by applying estimated HRs (specific to age-at-risk) for cause-specific mortality to the detailed mortality component of the US Centers for Disease Control and Prevention's WONDER database,^36^ which recorded 10 million deaths (from all causes) in more than 305 million individuals in the USA during 2007–10.^37^, ^38^ Results were modelled from age 40 years and enabled estimation of years of life lost between light drinkers (defined as those consuming >0–≤100 g/week of alcohol) and pre-defined groups of >100–≤200, >200–≤350, and >350 g per week. This method does not make use of the survival estimates from the modelled data; instead, it makes inferences by estimating age-at-risk specific HRs, which are then combined with external population age-specific mortality rates.^39^

Analyses used Stata (version 14.2 and 15.1). All p values presented are for 2-sided tests.

### Role of the funding source

The funders of the study did not have any role in the study design, data analysis, or reporting of this manuscript. AMW and SK had full access to the combined dataset, and, together with EDA and JD, had responsibility for the decision to submit the manuscript for publication.

## Results

Of the 786 787 participants with sufficient information for inclusion in this consortium, 186 875 (19%) reported not drinking at baseline, leaving 599 912 current drinkers without a history of cardiovascular disease at baseline who were eligible for the prespecified principal analysis. The current drinkers were derived from ERFC (247 504 participants), EPIC-CVD (26 036), and the UK Biobank (326 372; table 1). Baseline year of recruitment ranged from 1964 to 2010. The mean age of the participants was 57 years (SD 9). 265 910 (44%) of 599 912 participants were women, and 128 085 (21%) were current smokers (appendix p 12). About 50% reported drinking more than 100 g of alcohol per week, and 8·4% drank more than 350 g per week (table 1). During 5·4 million person-years (median 7·5 years of follow-up [5th–95th percentiles 5·0–18·4]), there were 40 310 deaths from all causes, (including 11 762 vascular and 15 150 neoplastic deaths), and 39 018 first incident cardiovascular disease outcomes, including 12 090 stroke events, 14 539 myocardial infarction events, 7990 coronary disease events excluding myocardial infarction, 2711 heart failure events, and 1121 deaths from other cardiovascular diseases (appendix p 13).

**Table 1:** Study-level and participant-level characteristics of the contributing data sources

		**ERFC**	**EPIC-CVD**	**UK Biobank**	**Participants with resurveys of alcohol consumption**
**Study level characteristics**					
Location		81 studies in 19 countries	22 centres in 10 European countries	England, Scotland, and Wales	37 studies in 15 countries
Years of recruitment		1964–2008	1990–2002	2006–10	1964–2010
Year of most recent endpoint follow-up		2013	2009	2016	2016
**Participant level characteristics**					
Total participants		356 819	30 702	358 833	89 499
Known current drinkers at baseline		247 504	26 036	326 372	71 011
Weekly baseline alcohol consumption in current drinkers					
	>0–≤25 g per week	53 418 (22%)	7906 (30%)	39 641 (12%)	12 301 (17% [11 g/week *vs* 36 g/week]‡)
	>25–≤50 g per week	33 953 (14%)	3704 (14%)	39 334 (12%)	8365 (12% [38 g/week *vs* 56 g/week]‡)
	>50–≤75 g per week	26 656 (11%)	2748 (11%)	42 907 (13%)	7322 (10% [63 g/week *vs* 80 g/week]‡)
	>75–≤100 g per week	16 557 (7%)	2446 (9%)	36 780 (11%)	6394 (9% [87 g/week *vs* 98 g/week]‡)
	>100–≤150 g per week	36 236 (15%)	2602 (10%)	55 815 (17%)	10 051 (14% [126 g/week *vs* 126 g/week]‡)
	>150–≤250 g per week	31 645 (13%)	3090 (12%)	60 025 (18%)	12 255 (17% [193 g/week *vs* 173 g/week]‡)
	>250–≤350 g per week	23 607 (10%)	1744 (7%)	26 669 (8%)	6927 (10% [303 g/week *vs* 248 g/week]‡)
	≥350 g per week	25 432 (10%)	1796 (7%)	25 201 (8%)	7396 (10% [515 g/week *vs* 354 g/week]‡)
**Baseline characteristics restricted to all current drinkers**					
Alcohol consumption (g/week), median (5th–95th percentiles)		87·7 (2·2–522·4)	61·9 (2·6–404·0)	103·9 (11·8–420·8)	105·2 (6·0–482·8)
Age (years) at baseline		57·1 (8·7)	55·0 (9·2)	56·5 (8·0)	55·3 (8·2)
Sex					
	Male	162 685 (66%)	13 508 (52%)	157 809 (48%)	44 360 (62%)
	Female	84 819 (34%)	12 528 (48%)	168 563 (52%)	26 651 (38%)
Smoking status					
	Not current	161 037 (65%)	17 608 (68%)	293 182 (90%)	50 930 (72%)
	Current	86 467 (35%)	8428 (32%)	33 190 (10%)	20 081 (28%)
History of diabetes					
	No	237 685 (96%)	24 875 (96%)	315 090 (97%)	68 159 (96%)
	Yes	9819 (4%)	1161 (4%)	11 282 (3%)	2852 (4%)
BMI, kg/m^2^		26·1 (3·8)	26·4 (4·1)	27·0 (4·4)	26·1 (3·8)
HDL-C, mmol/L		1·40 (0·41)	1·40 (0·42)	Not available*	1·41 (0·41)
Total cholesterol, mmol/L		5·80 (1·17)	6·11 (1·16)	Not available*	5·78 (1·08)
Systolic blood pressure, mm Hg		136·5 (19·0)	138·4 (21·3)	137·9 (18·5)	134·6 (18·4)
**Major outcomes restricted to current drinkers**					
All-cause mortality events		32 813	784†	6720	6912
All cardiovascular disease		18 791	12 758	7469	11 597

Data are n, n (%), or mean (SD), unless otherwise indicated. ERFC=Emerging Risk Factors Collaboration. EPIC-CVD=European Prospective Investigation into Cancer and Nutrition—Cardiovascular Disease. BMI=body-mass index. HDL-C=high-density-lipoprotein cholesterol.

* At the time of analysis, measurements of HDL-C and total cholesterol were not available in the UK Biobank.

† All-cause mortality events from EPIC derive only from the 13 670 participants in the random sub-cohort of EPIC-CVD, rather than from the entire EPIC prospective study.

‡ Mean consumption (g/week) at baseline *vs* resurvey.

Baseline alcohol consumption varied substantially across studies, was generally lower in more recent calendar periods of recruitment, and was positively skewed (median 96 g/week [5th–95th percentiles 6–448]; appendix p 22). It was weakly and positively correlated with male sex, smoking status and amount, systolic blood pressure, HDL-C level, fibrinogen, and lower socioeconomic status (appendix pp 23–24). 152 640 serial assessments of alcohol consumption were available for 71 011 participants from 37 studies (median interval between baseline and serial measurements 5·6 years [5th–95th percentiles 1·04–13·5]). Participants with serial measurements were younger, had slightly higher baseline alcohol consumption, and were more likely to be men than those without serial measurements (table 1, appendix p 14). The regression dilution ratio for alcohol consumption was 0·50 (95% CI 0·47–0·52), similar to that for systolic blood pressure (0·52, 0·50–0·55) but lower than that for HDL-C concentration (0·74, 0·72–0·76) in a common set of participants.

**Figure 1:**
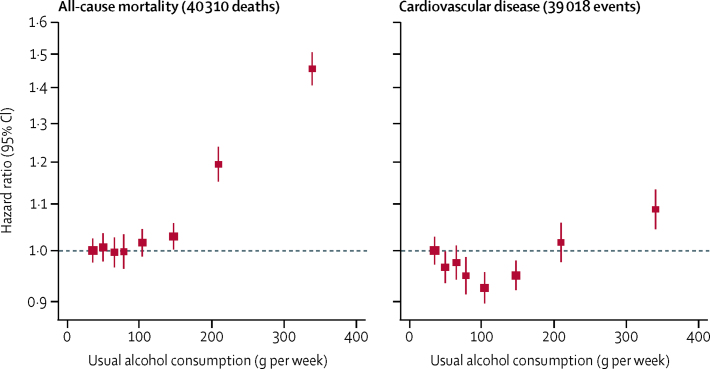
Associations of usual alcohol consumption with all-cause mortality and the aggregate of cardiovascular disease in current drinkers Cardiovascular disease was defined as an aggregate of myocardial infarction, coronary heart disease, and stroke. Hazard ratios are adjusted for age, smoking, and history of diabetes, and stratified by sex and EPIC centre. The reference category is the lowest baseline alcohol consumption category (between 0 and 25 g/week). HRs are plotted against the mean usual alcohol consumption in each category. Sizes of the boxes are proportional to the inverse of the variance of the log-transformed hazard ratios. Vertical lines represent 95% CIs.

**Figure 2:**
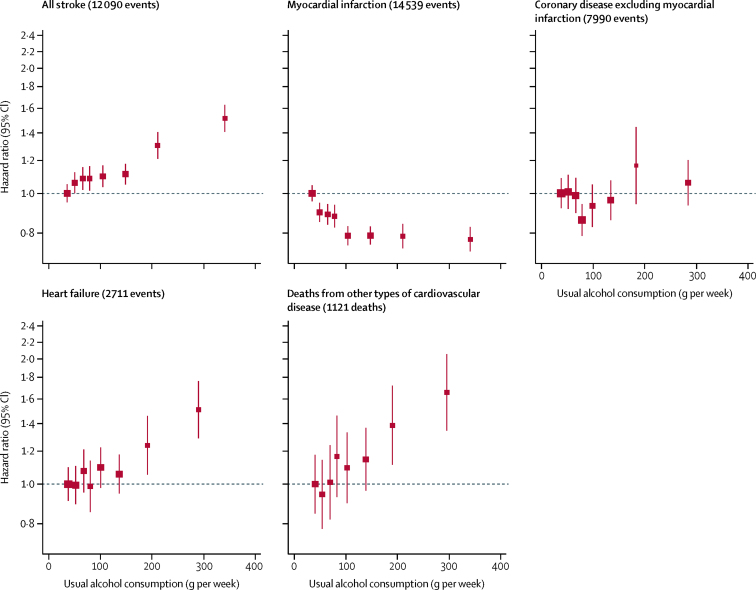
Associations of usual alcohol consumption with cardiovascular subtypes in alcohol drinkers Hazard ratios are adjusted for age, smoking, and history of diabetes, and stratified by sex and EPIC centre. The reference category is the lowest baseline alcohol consumption category (between 0 and 25g/week). Hazard ratios are plotted against the mean usual alcohol consumption in each category. Studies with fewer than five events of any outcome were excluded from the analysis of that outcome. Sizes of the boxes are proportional to the inverse of the variance of the log-transformed hazard ratios. Vertical lines represent 95% CIs. Deaths from other cardiovascular disease include the following outcomes: cardiac dysrhythmia, hypertensive disease, sudden death, and aortic aneurysm.

**Figure 3:**
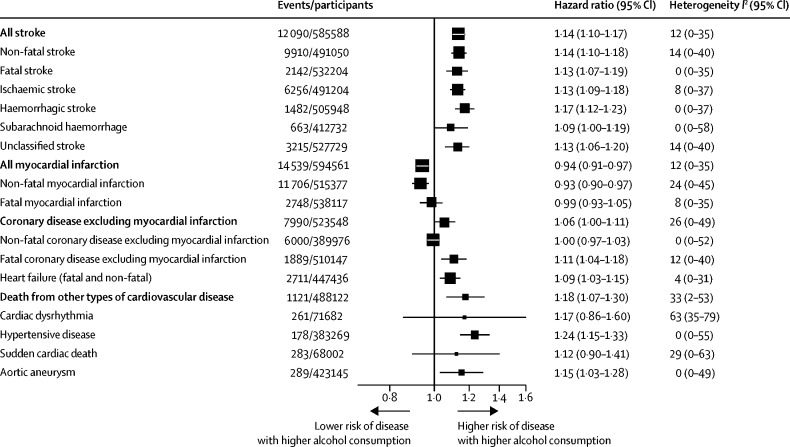
Hazard ratios for subtypes of cardiovascular outcomes in current drinkers, per 100 g per week higher usual alcohol consumption Hazard ratios are adjusted for age, smoking, and history of diabetes, and stratified by sex and centre. Studies with fewer than five events of any outcome were excluded from the analysis of that outcome.

For all-cause mortality, there was a positive and curvilinear association with alcohol consumption, with the lowest risk for those consuming below 100 g per week (figure 1, appendix p 25). Associations were similar for men and women (appendix p 26), but weaker at older ages (appendix p 27). There was a J-shaped association for the aggregate of cardiovascular disease outcomes (figure 1, appendix p 25). However, disaggregation showed two opposing sets of associations (figure 2). After adjustment for age, sex, smoking, and history of diabetes, the amount of alcohol consumed had positive and roughly linear associations with stroke (HR per 100 g/week higher consumption 1·14, 1·10–1·17), coronary disease excluding myocardial infarction (1·06, 1·00–1·11), heart failure (1·09, 1·03–1·15), fatal hypertensive disease (1·24, 1·15–1·33), and fatal aortic aneurysm (1·15, 1·03–1·28; Figure 2, Figure 3). By contrast, there was an inverse and approximately log-linear association with myocardial infarction (0·94, 0·91–0·97; Figure 2, Figure 3). Stroke associations were similar for fatal and non-fatal outcomes (appendix p 28) and across subtypes (appendix p 29). However, for coronary disease excluding myocardial infarction, associations were stronger for fatal than non-fatal outcomes (appendix p 28). For myocardial infarction, inverse associations were possibly more pronounced with non-fatal than fatal outcomes (figure 3, appendix p 28).

With the following notable exceptions, further adjustment for additional covariates did not substantially change HRs (table 2, appendix pp 15, 30). First, adjustment for HDL-C level weakened the inverse association between alcohol consumption and myocardial infarction, but strengthened the positive association between alcohol consumption and both coronary disease and heart failure. Second, adjustment for systolic blood pressure strengthened the inverse association between alcohol consumption and myocardial infarction, but weakened the positive associations between alcohol consumption and all other cardiovascular disease outcomes. Our analysis confirmed the established association of alcohol consumption with cancers of the digestive system, which did not change after additional adjustment for the factors listed above (appendix p 16). Furthermore, additional adjustment for smoking amount abolished the apparent association of alcohol consumption with lung cancer (appendix pp 16), in line with the accepted view that alcohol consumption does not cause lung cancer.^40^

**Table 2:** Hazard ratios for major cardiovascular outcomes in current drinkers, without and with adjustment for usual levels of systolic blood pressure, high-density-lipoprotein cholesterol, or body-mass index

	**All stroke**	**Myocardial infarction**	**Coronary disease excluding myocardial infarction**	**Heart failure**	**Deaths from other types of cardiovascular disease**
**Subset of participants with measurement of systolic blood pressure**					
Cohorts/events	70/11 297	73/13 519	46/7789	39/2668	44/1019
Basic adjustment*	1·16 (1·11–1·22)	0·95 (0·91–0·99)	1·06 (1·00–1·12)	1·11 (1·04–1·18)	1·16 (1·06–1·27)
Plus adjustment for systolic blood pressure	1·10 (1·06–1·14)	0·91 (0·87–0·94)	1·03 (0·97–1·10)	1·08 (1·02–1·15)	1·14 (1·03–1·25)
**Subset of participants with measurement of high-density-lipoprotein cholesterol**					
Cohorts/events	56/7982	61/9911	36/3608	29/1886	34/690
Basic adjustment*	1·16 (1·10–1·23)	0·93 (0·88–0·97)	1·07 (0·98–1·17)	1·09 (1·00–1·19)	1·22 (1·06–1·40)
Plus adjustment for high-density-lipoprotein cholesterol	1·17 (1·11–1·22)	1·00 (0·96–1·04)	1·13 (1·05–1·22)	1·14 (1·01–1·27)	1·22 (1·08–1·38)
**Subset of participants with measurement of body-mass index**					
Cohorts/events	68/11 733	71/14 217	43/7761	36/2566	42/1035
Basic adjustment*	1·15 (1·10–1·19)	0·95 (0·91–0·98)	1·06 (1·02–1·12)	1·12 (1·04–1·20)	1·16 (1·06–1·27)
Plus adjustment for body-mass index	1·14 (1·10–1·18)	0·94 (0·91–0·97)	1·06 (1·01–1·12)	1·10 (1·03–1·16)	1·16 (1·06–1·27)

Data are hazard ratio (95% CI) per 100 g per week higher usual alcohol consumption, unless otherwise indicated. Analyses were restricted to individuals with basic adjustment variables plus the additional variable. Studies with fewer than five events were excluded from the analysis of each outcome.

* Basic adjustment includes age, smoking, and history of diabetes, and stratification by sex and centre.

When including never-drinkers and ex-drinkers, we reproduced previously reported U-shaped associations of alcohol consumption with total cardiovascular disease and all-cause mortality (appendix p 31). However, we observed notable differences in baseline characteristics between never drinkers and current drinkers (eg, in relation to sex, ethnicity, smoking, and diabetes status; appendix p 12), supporting the validity of focusing on current drinkers in our main analysis. We recorded similar findings to those reported above in sensitivity analyses that involved the following approaches: used multiple imputation rather than complete-case analysis (appendix p 32); used fractional polynomials (appendix p 34); used a fixed-effect meta-analysis (appendix p 35); included studies that recorded fewer than five events for a particular outcome (appendix p 36); provided separate analyses of men and women (appendix p 17, appendix p 26); omitted outcomes recorded in the initial 5 years of follow-up (appendix p 18); excluded participants with diabetes or other known chronic diseases at baseline (appendix p 18); and restricted the analyses to studies that recorded both non-fatal and fatal endpoints (appendix p 37). Associations of baseline alcohol consumption with all-cause mortality were stronger in drinkers of beer or spirits than of wine, and in those drinking less frequently (when consuming the same weekly amount), including binge drinkers (appendix p 38). However, people showing these behaviours had higher baseline levels of smoking and other indicators of lower socioeconomic status, suggesting the potential for confounding effects (appendix pp 19–20). For cardiovascular disease subtypes, HRs tended to be higher in beer and spirit drinkers than in wine drinkers, but not significantly so in direct comparisons involving a common set of participants (appendix p 39).

We noted little heterogeneity in the studies contributing results for stroke (*I*^2^=12%), myocardial infarction (*I*^2^=12%), coronary disease excluding myocardial infarction (*I*^2^=26%), heart failure (*I*^2^=4%) or deaths from other types of cardiovascular disease (*I*^2^=33%; figure 3). HRs for the cardiovascular disease outcomes we studied were broadly similar for different geographical regions, decade of study enrolment, by data source (ie, ERFC, EPIC-CVD, and UK Biobank), and alcohol assessment method (appendix pp 40–42). HRs for the cardiovascular disease outcomes were generally higher at younger ages, but did not vary substantially by sex, history of diabetes, proatherogenic lipids, BMI, smoking status, or other individual-level characteristics (appendix pp 43–45). There was no evidence of small study effects (appendix p 46). Our data showed no evidence of violation of the proportional hazards assumption.

**Figure 4:**
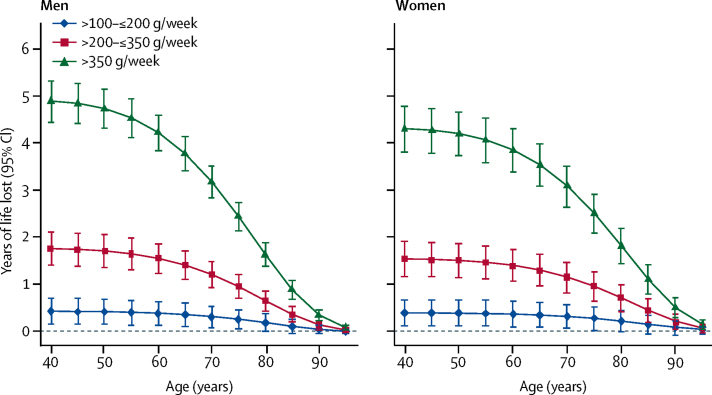
Estimated future years of life lost by extent of reported baseline alcohol consumption compared with those who reported consuming >0–≤100 g per week The estimates of cumulative survival from 40 years of age onwards in the alcohol-drinking groups were calculated by applying hazard ratios (specific to age at risk) for all-cause mortality associated with categorised baseline alcohol consumption to US death rates at the age of 40 years or older. Mean usual levels of alcohol consumption within each baseline alcohol consumption category were 56, 123, 208 and 367 g per week, respectively, for the groups >0–≤100 g per week, >100–≤200 g per week, >200–≤350 g per week, and >350 g per week.

In comparison to those who reported drinking >0–≤100 g (mean usual 56 g) alcohol per week, those who reported drinking >100–≤200 g (mean usual 123 g) per week, >200–≤350 g (mean usual 208 g) per week or >350 g (mean usual 367 g) per week had shorter life expectancy at age 40 years of approximately 6 months, 1–2 years, or 4–5 years respectively (figure 4). Similarly, men who reported consuming above the UK upper limit of 112 g per week had a shorter life expectancy at age 40 years of 1·6 years (95% CI 1·3–1·8), and men who reported drinking above the US upper limit of 196 g per week had a shorter life expectancy at age 40 years of 2·7 years (2·4–3·1) compared with men who reported drinking below these respective upper limits. Thus, men who reported drinking less than 100 g alcohol per week had about a 1–2 years longer life expectancy at age 40 years than those who reported drinking 196 g per week (appendix p 47). Women who reported drinking above either the UK threshold (112 g per week) or US threshold (98 g per week) had about 1·3 (1·1–1·5) years shorter life expectancy at age 40 years compared with women who reported drinking below these thresholds (appendix p 47). About 20% of the alcohol-related survival difference for men (and slightly less for women) was attributed to excess death from cardiovascular disease (appendix p 47). Similar findings to those for the US population were observed when modelling was based on EU mortality rates (data not shown).

## Discussion

The main finding of this analysis was that the threshold for lowest risk for all-cause mortality was about 100 g per week. For men, we estimated that long-term reduction of alcohol consumption from 196 g per week (the upper limit recommended in US guidelines) to 100 g per week or below was associated with about 1–2 years of longer life expectancy at age 40 years. Exploratory analyses suggested that drinkers of beer or spirits, as well as binge drinkers, had the highest risk for all-cause mortality.

Our study has highlighted the complex and diverse potential mechanisms by which alcohol consumption may exert cardiovascular effects.^41^, ^42^ It has shown that the association between alcohol consumption and total cardiovascular disease risk comprises several distinct and opposite dose–response curves, rather than a single J-shaped association. In particular, whereas higher alcohol consumption was roughly linearly associated with a higher risk of all stroke subtypes, coronary disease excluding myocardial infarction, heart failure, and several less common cardiovascular disease subtypes, it was approximately log-linearly associated with a lower risk of myocardial infarction. Our results are concordant with recent observational data and Mendelian randomisation studies.^16^, ^43^, ^44^, ^45^, ^46^

Our results contribute toward understanding of the basis for these directionally divergent cardiovascular disease associations. For example, our data have suggested that elevated systolic blood pressure could mediate alcohol consumption's positive association with stroke and coronary disease excluding myocardial infarction.^44^, ^47^, ^48^ By contrast, pathways related to HDL-C (but not necessarily HDL-C itself^49^, ^50^, ^51^, ^52^) could mediate alcohol consumption's inverse association with myocardial infarction. Both blood pressure and HDL-C are known to increase in response to alcohol consumption.^50^ They have contrasting associations with cardiovascular disease outcomes: the inverse association of HDL-C with cardiovascular disease is substantially stronger for coronary disease than stroke,^53^, ^54^ whereas the positive association of systolic blood with cardiovascular disease is considerably stronger for stroke than coronary disease.^55^ However, we did not find convincing evidence that other known risk factors were important mediators or confounders.

Our study's access to individual-participant data avoided limitations of previous literature-based reviews.^56^ To limit reverse causality, our study focused on current drinkers without baseline cardiovascular disease and omitted the initial period of follow-up. To limit confounding, our study adjusted for a variety of risk factors. To correct for misclassification in alcohol consumption and covariates, our study also used extensive information on serial assessments. Our results were robust to a variety of sensitivity analyses. Generalisability of the findings was enhanced by inclusion of data from 83 prospective studies based in many different high-income countries recruited between 1964 and 2010. Although alcohol consumption levels declined during this period, HRs were similar over calendar time.

Nevertheless, our study has some potential limitations. Self-reported alcohol consumption data are prone to bias and are challenging to harmonise across studies conducted over different time periods that used varying instruments and methods to record such data.^20^, ^57^ We did not, however, identify major differences in results across studies that used differing alcohol measurement instruments. Despite our study's access to extensive serial alcohol re-surveys from mid-life, our study could not investigate alcohol consumption during the entire life course. Misclassification in outcomes would have diluted dose-response associations, suggesting that true underlying associations of alcohol consumption with cardiovascular disease subtypes are stronger and more divergent than we observed. Because we did not generally have access to additional alcohol-related adverse outcomes (eg, non-fatal liver disease, injuries, or psychiatric comorbidities), we probably under-estimated potential benefits associated with lowering alcohol consumption. Because some individuals who reduced, but did not cease, alcohol consumption due to health complications were probably included in our analysis, we cannot exclude the effects of reverse causation (especially since some contributing studies did not record baseline chronic disease other than cardiovascular disease). Therefore, alternative study designs including randomised trials^58^ are needed, to control more completely for residual biases (including those related to studying ex-drinkers and never-drinkers).

In conclusion, our study shows that among current drinkers, the threshold for lowest risk of all-cause mortality was about 100 g per week. For cardiovascular disease subtypes other than myocardial infarction, there were no clear thresholds below which lower alcohol consumption stopped being associated with a lower disease risk. These data support adoption of lower limits of alcohol consumption than are recommended in most current guidelines.
