# Department of Error

**DOI:** 10.1016/S0140-6736(18)31168-1

**Published:** 2018-06-02

*Wood AM, Kaptoge S, Butterworth AS, et al. Risk thresholds for alcohol consumption: combined analysis of individual-participant data for 599 912 current drinkers in 83 prospective studies.* Lancet *2018; **391**: 1513–23—*In this Article, Makoto Daimon's affiliation was listed incorrectly and should have been: Global Center of Excellence Program Study Group, Yamagata University Faculty of Medicine, Yamagata, Japan. The online version has been corrected as of May 31, 2018.

