# Supplementary material for: Risk thresholds for alcohol consumption: combined analysis of individual-participant data for 599 912 current drinkers in 83 prospective studies
Source: Lancet. 2018 Apr 14;391(10129):1513–23. doi: 10.1016/S0140-6736(18)30134-X (PMC5899998; doi:10.1016/S0140-6736(18)30134-X)
Supplement: Supplementary appendix [file mmc1.pdf]

THE LANCET

Supplementary appendix

This appendix formed part of the original submission and has been peer reviewed. We post it as supplied by the authors.

Supplement to: Wood AM, Kaptoge S, Butterworth AS, et al. Risk thresholds for alcohol consumption: combined analysis of individual-participant data for 599 912 current drinkers in 83 prospective studies. *Lancet* 2018; **391:** 1513–23.

Annex 1: Harmonisation of drinking amount across the contributing studies

Emerging Risk Factors Collaboration

Data on alcohol were harmonised at the ERFC coordinating centre in consensus with the individual study collaborators. Studies used a variety of questionnaire-based approaches (eg, self-administered *vs* interview-led questionnaires; food frequency questionnaires *vs* dietary recall surveys) to provide information on alcohol consumption, which included semi-quantitative information (eg, amount in a given period, frequency of drinks in a given period, categories for amount or frequency) of different types of alcoholic drinks (ie, beer, wine, cider, spirits/liquor, alcopops, long drink, fortified wine, liqueur, sake, shochu, tharra, aperitif/digestif) (eTable 1). The available information was harmonised into variables denoting (in order of precedence): amount, status, duration, stop age, start age, years stopped, usage frequency. When information was provided as semi-quantitative categories of intake, alcohol amount was assigned based on the mid-points of bounded categories or the lower bound of an open-ended highest category. Alcohol status was categorised as “never”, “never/ex”, “ex”, “ex/current” and “current” drinkers. The alcohol status categories “never/ex” and “ex/current” included studies that did not definitively distinguish between never and ex drinkers, or between ex and current drinkers, respectively. Subsequently, drinking amount was set to missing for participants with “ex/current” drinking status as it was not possible to distinguish current drinking amount. Information on alcohol amount was converted to a UK standard scale of grams/week (1 unit=8 grams of ethanol). Alcohol status and amount were cross-referenced with each other to resolve ambiguous data and update missing information.

EPIC-CVD

Intake of alcoholic drinks at baseline was calculated from validated country-specific dietary questionnaires aimed to capture specificity of local dietary habits. The number of standard glasses of alcoholic drinks (beer, cider, wine, sweet liquor, distilled spirits or fortified wines) consumed per day/week during the 12 months prior to recruitment were reported by participants. In each country, intake was calculated based on the estimated ethanol content and usual glass volume for each type of alcoholic beverage[1]. To this purpose, information from highly standardized 24-hr dietary recalls from a subset of the cohort was used. Information on lifetime alcohol intake were collected with lifestyle questionnaires administered at baseline. Information on lifetime alcohol consumption was assessed as number of glasses of different drinks consumed at 20, 30, 40 and 50 years of age consumed per week, and then computed as a weighted usual and expressed as grams per week. Information on alcohol amount was then converted to a standard scale of grams/week (1 unit=8 grams of alcohol).

UK Biobank

Intake of alcoholic drinks at baseline was obtained from a touchscreen questionnaire which was used to extract information on status, intake frequency (per month) and beverage type (ie, red wine, white wine/champagne, beer, spirits, fortified wine). See https://biobank.ctsu.ox.ac.uk/crystal/docs/TouchscreenQuestionsMainFinal.pdf. Information on total alcohol amount was then calculated and converted to a standard scale of grams/week (1 unit=8 grams of alcohol).

[1]Bergmann MM, Rehm J, Klipstein-Grobusch K, Boeing H, Schütze M, Drogan De , et al. The association of pattern of lifetime alcohol use and cause of death in the European prospective investigation into cancer and nutrition (EPIC) study. Int J Epidemiol. 2013;42(6): 1772–1790.

Annex 2 ERFC Study Acronyms

ARIC, Atherosclerosis Risk in Communities Study
AFTCAPS, Air Force/Texas Coronary Atherosclerosis Prevention Study
ATENA, cohort of Progetto CUORE
ATTICA, ATTICA study
AUSDIAB, Australian Diabetes, Obesity and Lifestyle Study
BHS, Busselton Health Study
BRUN, Bruneck Study
BWHHS, British Women's Heart and Health Study
CAPS, Caerphilly Prospective Study
CASTEL, Cardiovascular Study in the Elderly
CHARL, Charleston Heart Study
CHS1, CHS2, Cardiovascular Health Study I and II
COPEN, Copenhagen City Heart Study
CONOR, COhorts of NORway (5 cohorts: FINNMARK, HUBRO, OPPHED, OSLO2, TROMS)
CUORE, Progetto CUORE (4 cohorts: ATENA, MATISS83, MATISS87, MATISS93)
DESIR, Data from an Epidemiological Study on the Insulin Resistance Syndrome
DRECE, Diet and Risk of Cardiovascular Disease in Spain
DUBBO, Dubbo Study of the Elderly
EAS, Edinburgh Artery Study
EPESEBOS, The Established Populations for the Epidemiologic Study of the Elderly Studies, Boston
EPESEIOW, The Established Populations for the Epidemiologic Study of the Elderly Studies, Iowa
EPESENCA, The Established Populations for the Epidemiologic Study of the Elderly Studies, North Carolina
EPESENHA, The Established Populations for the Epidemiologic Study of the Elderly Studies, New Haven
ESTHER, Epidemiologische Studie zu Chancen der Verhütung und optimierten Therapie chronischer Erkrankungen in der älteren Bevölkerung
FINMARK, cohort of CONOR
FINRISK92, Finrisk Cohort 1992
FINRISK97, Finrisk Cohort 1997
FLECTHER, Fletcher Challenge Blood Study
FUNAGATA, Funagata Study
GOLSTRUP, Golstrup Study
GREPCO, cohort of Risk Factors and Life Expectancy Pooling Project
HBS, Helsinki Businessmen Study
HCS, Hertfordshire Cohort Study
HIMS, Health in Men Study
HISAYAMA, Hisayama Study
HONOL, Honolulu Heart Program
HUBRO, cohort of CONOR
IKNS, Ikawa, Kyowa, and Noichi Study
KARELIA, North Karelia Project
KIHD, Kuopio Ischaemic Heart Disease Study
LASA, Longitudinal Aging Study Amsterdam
MATISS83/87/93, cohort of Progetto CUORE
MESA, Multi-Ethnic Study of Atherosclerosis
MCVDRFP, Monitoring of CVD Risk Factors Project
MICOL, cohort of Risk Factors and Life Expectancy Pooling Project
MONICA_KORA1, MONICA/KORA Augsburg Surveys S1
MONICA_KORA2, MONICA/KORA Augsburg Surveys S2
MONICA_KORA3, MONICA/KORA Augsburg Surveys S3
MORGEN, Monitoring Project on Chronic Disease Risk Factors
MRCOLD, MRC Study of Older People
MRFIT, Multiple Risk Factor Intervention Trial 1
NFR, cohort of Risk Factors and Life Expectancy Pooling Project
NHANES I, First National Health and Nutrition Examination Survey
NHANES III, Third National Health and Nutrition Examination Survey
NPHSII, Northwick Park Heart Study II
NSHS, Nova Scotia Health Survey
OPPHED, cohort of CONOR
OSAKA, Osaka Study
OSLO2, cohort of CONOR

PRHHP, Puerto Rico Heart Health Program
PRIME, Prospective Epidemiological Study of Myocardial Infarction
PROCAM, Prospective Cardiovascular Münster Study
PROSPER, Prospective Study of Pravastatin in the Elderly at Risk
QUEBEC, Quebec Cardiovascular Study
RANCHO, Rancho Bernardo Study
RS_I, The Rotterdam Study I
RS_II, The Rotterdam Study II
RS_III, The Rotterdam Study III
SHHEC, Scottish Heart Health Extended Cohort
SHIP, Study of Health in Pomerania
TOYAMA, Toyama Study
TROMS, cohort of CONOR
TROMSØ, Tromsø Study
ULSAM, Uppsala Longitudinal Study of Adult Men
WHITE I, Whitehall I Study
WHITE II, Whitehall II Study
WHIHABPS, Women's Health Initiative (Hormones and Biomarkers Predicting Stroke in Women)
WCWC, Württemberg Construction Workers Cohort
WOSCOPS, West of Scotland Coronary Prevention Study
ZUTE, Zutphen Elderly Study

Annex 3: Definitions of major incident outcomes considered

End point (includes both fatal and non-fatal)	ICD-10 codes
**All cardiovascular**	G45, I01, I03-I82, I87, I95-I99, F01, Q20-Q28, R96
**Myocardial infarction (MI)**	I21, I22, I23
**Coronary disease non-MI**	I24-I25
**All stroke**	F01, I60-I69
Ischaemic stroke	I63
Haemorrhagic stroke	I61
Subarachnoid haemorrhage	I60
Unclassified stroke†	I64
**Heart failure**	I50
**Other vascular deaths**	I47-I49, I10-I15, R96, I71, I50
Cardiac dysrhythmia	I47-I49
Hypertensive disease	I10-I15
Sudden death	R96
Aortic aneurysm	I71

† Unclassified stroke refers to ICD codes I64 (ICD-10), 436 (ICD-9) or earlier ICD equivalents, or strokes not specified as ischemic or haemorrhagic in study specific codes.

Corresponding ICD-6, 7 or 8 codes are used for ERFC studies that recorded outcomes using earlier ICD versions.

Annex 4. Statistical methods used for estimating years of life lost

We used three pieces of information to estimate reductions in life expectancy associated with alcohol consumption at baseline (henceforth “exposure groups” pre-defined as alcohol consumption >0-≤100, >100-≤200, >200-≤350 and >350 grams/week):

(i) age-at-risk specific hazard ratios for all-cause (and cause-specific) mortality in each exposure group versus the reference (derived from the ERFC and UK Biobank);

(ii) population all-cause (and cause-specific) mortality rates (derived from the detailed mortality component of the CDC WONDER database of the US Centers for Disease Control and Prevention); and

(iii) prevalence of exposure groups in the population (derived from the ERFC and UK Biobank).

We estimated population survival curves for each exposure group, utilising estimated age-at-risk specific hazard ratios for mortality by exposure groups in the ERFC, and UK Biobank and routine statistics on overall population mortality rates. We estimated reductions in life-expectancy as differences in areas under any two survival curves compared. To calculate an appropriate mortality rate for the reference group (i.e. defined as those drinking >0-≤100 grams/week), we used ERFC and UK Biobank data on exposure prevalence estimates, as described below.

Age-at-risk specific hazard ratios for mortality by exposure groups were estimated from ERFC and UK Biobank data separately for each sex. Specifically, a Cox regression model stratified by cohort and trial arm (where applicable) was fitted separately for each sex using a dataset in which participant ages-at-risk were deterministically updated by splitting the follow up times every 5-years and recalculating an age-at-risk variable at the beginning of each 5-year interval of follow up. Interactions between baseline exposure groups and linear and quadratic terms for the age-at-risk variable were included in the model to obtain smoothed hazard ratios. Thus, for participant i in stratum s with exposure group indicator variable $E_{si(j)}$ (i.e. dummy variable equal to 1 if in exposure group is j and zero otherwise) the log hazard rate at time t since baseline was modelled as:

$$\log\big(h_{si}(t)\big) = \log\big(h_{s0}(t)\big) + \sum_{j=1}^{3}\gamma_{0j}\,E_{si(j)} + \beta_1 agerisk_{si} + \beta_2 agerisk_{si}^2 + \sum_{j=1}^{3}\gamma_{1j}\,E_{si(j)} \times agerisk_{si} + \sum_{j=1}^{3}\gamma_{2j}\,E_{si(j)} \times agerisk_{si}^2 \qquad (1)$$

from which the age-at-risk specific hazard ratios (and 95% CIs) for mortality were obtained as linear combinations of the relevant estimated coefficients, with age-at-risk fixed at values corresponding to midpoints of 5-year age-groups from age 40 onwards.

Population all-cause (and cause-specific) mortality rates per 100,000 were obtained in 5-year age-groups for the US population during years 2007-2010 from the Center for Disease Control (CDC) WONDER online database (https://wonder.cdc.gov/ucd-icd10.html), as well as for 15 EU countries during year 2000 (http://ec.europa.eu/eurostat/data/database). Because the mortality rates were provided only up to age-group 80-84 years, but we desired to estimate the overall population survival curves, we used a Poisson regression model with linear and quadratic terms for the midpoints of 5-year age-groups to smooth and extrapolate the mortality rates. Next, assuming exponential survival (i.e. constant hazard) within each 5-year age group, we estimated the age-specific survival probability as $S_a = exp(-5 \times IR_a)$ and derived the overall population survival curves from age 35 onwards as the product of the relevant age-group specific survival probabilities.

$$p(survival|agerisk \geq 35) = \prod_{agerisk \geq 35} S_a \qquad (2)$$

In order to infer population mortality rates appropriate for the reference exposure group used in our estimation of age-specific hazard ratios (i.e. defined as those drinking >0-≤100 grams/week), we used logistic regression to model the age-specific prevalence of the alchol consumption categories in ERFC and UK Biobank cohorts by sex and decade of recruitment. We used the age-specific prevalence estimates for the decade commencing in the year 1990 to infer the age-specific mortality rates appropriate for our reference group IR_{a0} as:[1]

$$IR_{a0} = \frac{IR_a}{p_{a0} + \sum_{j=1}^{3} p_{aj} \times RR_{aj}} \qquad (3)$$

Where IR_a is the population mortality rate for age group a, p_{aj} is the age-specific prevalence of exposure group j, and RR_{aj} is the age-specific hazard ratio in comparison of exposure group j versus reference group ($j = 0$). The age-specific mortality rates in each of the non-reference exposure groups were then inferred in turn by multiplying the age-specific mortality rate for the reference group IR_{a0} by the age-specific hazard ratios RR_{aj} based on ERFC and UK Biobank data and equation (2) above used to infer the exposure group-specific population survival curves. Finally, reductions in life expectancy according to baseline exposure groups were estimated as difference in the areas under the survival curves for the reference group and each of the non-reference exposure groups in turn. The areas under curves were calculated by numerical integration.

Monte Carlo simulation was used to calculate confidence intervals for the estimated reductions in life expectancy, taking into account uncertainty in the age-at-risk specific hazard ratios calculated from equation (1) above. In particular, new parameter estimates were randomly drawn from the multivariate normal distribution defined by the fitted model mean and covariance matrix, 200 times, and the above procedure repeated for each draw to calculate reductions in life-expectancy for each index age of interest. Assuming asymptotic normality, the standard deviation of the 200 Monte Carlo estimates of reductions in life expectancy for each index age were used to calculate 95% confidence intervals around the originally estimated value. Histograms were inspected to judge that normality assumption was reasonable.

Appendix References

1 Woloshin S, Schwartz LM, Welch HG. The risk of death by age, sex, and smoking status in the United States: putting health risks in context. J Natl Cancer Inst 2008;100(12):845-53.

Annex 5. Supplementary Tables/Figures

eTable 1: Alcohol consumption ascertainment methods for 83 studies in the ERFC, EPIC-CVD and UK Biobank.

eTable 2: Summary of individual-level baseline characteristics, mortality and major cardiovascular outcomes by baseline alcohol consumption categories.

eTable 3: Summary of events for each study (83 studies), restricted to current drinkers.

eTable 4. Comparison of baseline characteristics of individuals used in main analysis versus individuals with repeat measures of alcohol consumption or measures of lifetime alcohol consumption from the contributing data sources.

eTable 5. Hazard ratios for cardiovascular outcomes amongst current drinkers, without and with adjustment for usual or baseline levels of potential confounders, mediators and proxies thereof.

eTable 6. Hazard ratios for death from lung cancer and digestive related cancer outcomes per 100 grams/wk higher usual alcohol consumption amongst current drinkers, without and with adjustment for usual or baseline levels of potential confounders, mediators and proxies thereof.

eTable 7: Sex-specific hazard ratios for major cardiovascular outcomes per 100 grams/week increase in usual alcohol consumption amongst current drinkers.

eTable 8: Sensitivity analyses: Hazard ratios for major cardiovascular outcomes per 100 grams/week increase in usual alcohol consumption amongst current drinkers.

eTable 9: Baseline characteristics by frequency of baseline alcohol consumption.

eTable 10: Baseline characteristics by type of baseline alcohol predominantly consumed.

eFigure 1: Flow diagram of study selection process in current analysis.

eFigure 2: Box plots of baseline alcohol consumption amongst current drinkers from 83 studies by decade of first baseline survey.

eFigure 3a: Cross-sectional associations between baseline alcohol consumption and continuous baseline characteristics.

eFigure 3b: Cross-sectional associations between baseline consumption and categorical baseline characteristics.

eFigure 4: Shape of association of baseline alcohol consumption with all-cause mortality and all cardiovascular disease amongst current drinkers.

eFigure 5. Shape of association of usual alcohol consumption with all-cause mortality for males and females.

eFigure 6. Shape of association of usual alcohol consumption with all-cause mortality by age-specific groups.

eFigure 7. Shapes of associations of usual alcohol consumption with fatal and non-fatal major cardiovascular causes.

eFigure 8. Shapes of associations of usual alcohol consumption with type of stroke.

eFigure 9a: Hazard ratios per 100 grams/week higher usual alcohol consumption for subtypes of cardiovascular outcomes amongst current drinkers, adjusted for body mass index.

eFigure 9b. Shape of association of usual alcohol consumption with all-cause mortality and all cardiovascular disease amongst current drinkers, adjusted for body mass index.

eFigure 10: Shape of association between baseline alcohol consumption, including ex- and non-drinkers, with all-cause mortality and cardiovascular disease.

eFigure 11: Hazard ratios per 100 grams/week higher baseline alcohol consumption for subtypes of cardiovascular outcomes amongst current drinkers with recorded baseline alcohol consumption (left) compared against all current drinkers using multiple imputation (right).

eFigure 12: Shapes of associations of baseline alcohol consumption with stroke and coronary outcomes amongst alcohol drinkers.

eFigure 13: Best fitting 2nd degree fractional polynomial for the modelled shape of association between baseline alcohol consumption with all-cause mortality.

eFigure 14: Hazard ratios per 100 grams/week higher usual alcohol consumption for subtypes of cardiovascular outcomes amongst current drinkers from a fixed-effect meta-analysis.

eFigure 15: Hazard ratios per 100 grams/week higher usual alcohol consumption for subtypes of cardiovascular outcomes amongst current drinkers, from fixed-effect analysis with inclusion of studies with fewer than 5 outcomes of a particular type.

eFigure 16: Shape of association between usual alcohol consumption with major vascular restricted to ERFC studies recording both coronary death and non-fatal MI endpoints.

eFigure 17: Shapes of associations of baseline alcohol consumption with all-cause mortality by (a) consumption frequency, (b) consumption type and (c) binge drinking status.

eFigure 18: Hazard ratios per 100 grams/week higher alcohol consumption for all-cause mortality and different cardiovascular outcomes amongst current drinkers and by alcohol type.

eFigure 19a-e: Hazard ratios per 100 gram/week increase in usual alcohol consumption for major vascular outcomes and all cause mortality amongst current drinkers by study/cohort-level characteristics.

eFigure 20a-e: Hazard ratios per 100 gram/week increase in usual alcohol consumption for major cardiovascular outcomes amongst current drinkers by individual-level characteristics.

eFigure 21. Funnel plots and assessment of small-study effects for study-specifc hazard ratios per 100 gram/week increase in usual alcohol consumption for major vascular outcomes amongst current drinkers.

eFigure 22. Estimated future years of life lost in individuals reporting drinking above a range of hypothetical alcohol consumption thresholds compared to those reporting drinking less than the hypothetical alcohol consumption thresholds.

eTable 1: Alcohol consumption ascertainment methods for 83 studies in the ERFC, EPIC-CVD and UK Biobank.

Study	Ascertainment method of alcohol consumption	Format of ascertainment	Calculated or Reported[1]
AFTCAPS	Questionnaire	Self administered	Calculated
ARIC	Dietary survey	Interview	Calculated
ATENA	FFQ	Self administered	Reported
ATTICA	FFQ	Self administered	Calculated
AUSDIAB	FFQ	Self administered	Calculated
BHS	Lifestyle questionnaire	Self administered	Reported
BRUN	Questionnaire<br>FFQ<br>Diet record	Interview<br>Interview<br>Self administered	Calculated
BWHHS	Questionnaire	Self administered	Calculated
CAPS	Questionnaire	Unknown	Calculated
CASTEL	Questionnaire	Self administered	Reported
CHARL	Dietary survey/ questionnaire	Interview / Self administered	Calculated
CHS1	Unknown	Unknown	Calculated
CHS2	Unknown	Unknown	Calculated
COPEN	Questionnaire	Self administered	Reported
DESIR	Questionnaire	Self administered	Unknown
DRECE	24hr recall / FFQ	Interview	Calculated
DUBBO	Questionnaire	Interview	Calculated
EAS	Questionnaire	Self administered	Calculated
EPESEBOS	Questionnaire	Interview	Reported
EPESEIOW	Questionnaire	Interview	Reported
EPESENCA	Questionnaire	Interview	Reported
EPESENHA	Questionnaire	Interview	Reported
EPIC-CVD	24hr recall / FFQ / 7-day diary	Interview / Self administered	Calculated
ESTHER	FFQ	Self administered	Calculated
FINNMARK	Questionnaire	Self administered	Calculated
FINRISK92	Questionnaire	Self administered	Reported
FINRISK97	Questionnaire	Self administered	Reported
FLETCHER	Questionnaire	Self administered	Calculated
FUNAGATA	Unknown	Unknown	Unknown
GLOSTRUP	Questionnaire	Self administered	Calculated
GREPCO	Questionnaire	Self administered	Reported
HBS	FFQ	Self administered	Calculated
HCS	Questionnaire	Self administered	Unknown
HIMS	Questionnaire	Self administered	Reported
HISAYAMA	FFQ	Self administered	Calculated
HONOL	Questionnaire	Interview	Calculated
HPFS	FFQ	Self administered	Calculated
HUBRO	Questionnaire	Self administered	Calculated
IKNS	Questionnaire	Interview	Calculated
KARELIA	Questionnaire	Self administered	Calculated
KIHD	Questionnaire	Self administered	Reported
LASA	Questionnaire	Interview	Calculated
MATISS83	FFQ / dietary recall	Self administered / Interview	Reported
MATISS87	FFQ / dietary recall	Self administered / Interview	Reported
MATISS93	FFQ / dietary recall	Self administered / Interview	Reported
MCVDRFP	Questionnaire	Self administered	Calculated
MESA	FFQ	Interview / Self administered	Calculated
MONICA_KORA1	Dietary survey	Interview	Calculated
MONICA_KORA2	Dietary survey	Interview	Calculated
MONICA_KORA3	Dietary survey	Interview	Calculated
MICOL	Questionnaire	Self administered	Reported
MRCOLD	Questionnaire	Interview	Calculated
MRFIT	Questionnaire	Self administered	Calculated

Continued over page

eTable 1 (continued): Alcohol consumption ascertainment methods for 83 studies in the ERFC, EPIC-CVD and UK Biobank.

Study	Ascertainment method of alcohol consumption	Format of ascertainment	Calculated or Reported[1]
NFR	Unknown	Self administered	Reported
NHANES I	Questionnaire	Interview	Calculated
NHANES III	Questionnaire	Interview	Calculated
NPHS II	Questionnaire	Self administered	Calculated
NSHS	FFQ	Self administered	Reported
OPPHED	Questionnaire	Self administered	Calculated
OSAKA	Questionnaire	Interview	Calculated
OSLO2	Questionnaire	Self administered	Calculated
PRHHP	24hr recall	Interview	Calculated
PRIME	Quantitative recall frequency questionnaire	Interview	Calculated
PROCAM	Questionnaire	Unknown	Calculated
PROSPER	Questionnaire	Unknown	Calculated
QUEBEC	Questionnaire	Self administered	Calculated
RANCHO	Questionnaire	Interview	Calculated
RS_I	Dietary interview	Interview	Calculated
RS_II	Dietary interview	Interview	Unknown
RS_III	Dietary interview	Interview	Unknown
SHHEC	7-day recall	Self administered	Calculated
SHIP	Unknown	Unknown	Unknown
TOYAMA	Questionnaire	Self administered	Unknown
TROMS	Questionnaire	Self administered	Calculated
TROMSØ	Questionnaire	Self administered	Calculated
ULSAM	FFQ	Self administered	Reported
UK Biobank	Questionnaire	Self administered	Reported
WCWC	Questionnaire	Interview	Unknown
WHIHABPS	FFQ	Self administered	Calculated
WHITE I	FFQ/ Dietary recall	Self administered	Calculated
WHITE II	FFQ	Self administered	Calculated
WOSCOPS	Dietary recall	Self administered	Reported
ZUTE	Cross-check dietary history	Interview	Calculated

[1]Calculated: alcohol amount is the product of the reported frequency (eg, more than once per day, more than once per month) and the individual reported intake per occasion (eg, 2 glasses on each occasion).
Reported: alcohol amount is provided within a specified time period (eg, number of glasses in the past week)
FFQ=food frequency questionnaire.

eTable 2: Summary of individual-level baseline characteristics, mortality and major cardiovascular outcomes by baseline alcohol consumption categories.

	Ex-drinkers at baseline		Never-drinkers at baseline		All current drinkers at baseline		>0-≤50g/wk		>50-≤100g/wk		>100-≤150g/wk		>150-≤250g/wk		>250-≤350g/wk		≥350g/wk	
**Characteristics**	**N**	**Mean (SD) / %**	**N**	**Mean (SD) / %**	**N**	**Mean (SD) / %**	**N**	**Mean (SD) / %**	**Ns**	**Mean (SD) / %**	**N**	**Mean (SD) / %**	**N**	**Mean (SD) / %**	**N**	**Mean (SD) / %**	**N**	**Mean (SD) / %**
Age at baseline (years)	29,726	60.0 (8.8)	53,851	58.0 (9.8)	599,912	57.2 (8.7)	177,956	57.3 (9.3)	128,094	57.0 (8.6)	94,653	57.4 (8.4)	94,760	57.2 (8.2)	52,020	56.6 (8.2)	52,429	56.4 (7.9)
Sex	29,726		53,851		599,912		177,956		128,094		94,653		94,760		52,020		52,429	
Male	14,542	48.9%	15,962	29.6%	334,002	55.7%	70,698	39.7%	59,458	46.4%	53,158	56.2%	64,253	67.8%	40,332	77.5%	46,103	87.9%
Female	15,184	51.1%	37,889	70.4%	265,910	44.3%	107,258	60.3%	68,636	53.6%	41,495	43.8%	30,507	32.2%	11,688	22.5%	6,326	12.1%
Ethnicity	21,577		37,730		453,102		118,519		97,754		75,412		76,561		42,894		41,962	
White	17,227	79.8%	19,685	52.2%	420,668	92.8%	106,584	89.9%	92,349	94.5%	71,898	95.3%	71,148	92.9%	39,600	92.3%	39,089	93.2%
Non-white	4,350	20.1%	18,045	47.8%	32,434	7.2%	11,935	10.1%	5,405	5.5%	3,514	4.7%	5,413	7.1%	3,294	7.7%	2,873	6.9%
Smoking status	29,726		53,851		599,912		177,956		128,094		94,653		94,760		52,020		52,429	
Not current	23,618	79.5%	45,991	85.4%	471,827	78.7%	144,698	81.3%	106,747	83.3%	76,480	80.8%	73,888	78.0%	37,061	71.2%	32,953	62.9%
Current	6,108	20.5%	7,860	14.6%	128,085	21.3%	33,258	18.7%	21,347	16.7%	18,173	19.2%	20,872	22.0%	14,959	28.8%	19,476	37.1%
Level of education	25,540		36,845		519,896		155,700		112,538		82,316		81,392		43,992		43,958	
No schooling/Primary	2,359	9.2%	6,863	18.6%	43,468	8.4%	11,555	7.4%	4,859	4.3%	7,569	9.2%	4,319	5.3%	7,043	16.0%	8,123	18.5%
Secondary	13,696	53.6%	17,140	46.5%	208,928	40.2%	68,795	44.2%	43,851	39.0%	30,336	36.9%	31,087	38.2%	16,944	38.5%	17,915	40.8%
Vocational/ University	9,485	37.1%	12,842	34.9%	267,500	51.4%	75,350	48.4%	63,828	56.7%	44,411	54.0%	45,986	56.5%	20,005	45.5%	17,920	40.8%
Occupation	21,821		38,723		456,400		125,046		101,556		71,196		78,116		40,431		40,055	
Not working	10,105	46.3%	17,732	45.8%	158,781	34.8%	46,712	37.4%	36,082	35.5%	24,915	35.0%	25,441	32.6%	12,911	31.9%	12,720	31.8%
Manual	2,292	10.5%	6,574	17.0%	54,701	12.0%	12,299	9.8%	8,729	8.6%	7,604	10.7%	9,910	12.7%	7,421	18.4%	8,738	21.8%
Office	6,389	29.3%	8,951	23.1%	189,885	41.6%	47,646	38.1%	45,163	44.5%	31,592	44.4%	35,221	45.1%	15,556	38.5%	14,707	36.7%
Other	3,035	13.9%	5,466	14.1%	53,033	11.6%	18,389	14.7%	11,582	11.4%	7,085	10.0%	7,544	9.7%	4,543	11.2%	3,890	9.7%
Total physical activity	1,253		1,962		23,796		9,756		4,926		2,539		3,051		1,734		1,790	
Inactive	136	10.9%	102	5.2%	4,426	18.6%	1,335	13.7%	946	19.2%	586	23.1%	703	23.0%	453	26.1%	403	22.5%
Moderately inactive	329	26.3%	372	19.0%	7,484	31.5%	3,014	30.9%	1,532	31.1%	839	33.0%	964	31.6%	541	31.2%	594	33.2%
Moderately active	662	52.8%	1,279	65.2%	9,728	40.9%	4,483	46.0%	2,009	40.8%	904	35.6%	1,114	36.5%	583	33.6%	635	35.5%
Active	126	10.1%	209	10.7%	2,158	9.1%	924	9.5%	439	8.9%	210	8.3%	270	8.9%	157	9.1%	158	8.8%
History of diabetes	29,726		53,851		599,912		177,956		128,094		94,653		94,760		52,020		52,429	
No	26,932	90.6%	50,042	92.9%	577,650	96.3%	170,595	95.9%	124,004	96.8%	91,413	96.6%	91,479	96.5%	49,965	96.1%	50,194	95.7%
Yes	2,794	9.4%	3,809	7.1%	22,262	3.7%	7,361	4.1%	4,090	3.2%	3,240	3.4%	3,281	3.5%	2,055	4.0%	2,235	4.3%
SBP (mmHg)	28,561	137 (20)	52,205	137 (20)	588,675	136 (19)	173,510	135 (19)	126,769	135 (19)	93,401	137 (19)	93,153	137 (18)	51,216	137.9 (19)	51,432	140 (19)
HDL-C (mmol/l)	13,208	1.31 (0.37)	26,611	1.38 (0.36)	221,727	1.38 (0.39)	79,285	1.34 (0.38)	38,518	1.38 (0.39)	32,916	1.40 (0.40)	27,485	1.40 (0.39)	20,895	1.43 (0.39)	22,628	1.44 (0.40)
BMI (kg/m^2)	28,862	26.2 (5.1)	52,735	26.5 (4.8)	589,621	26.1 (4.2)	173,729	26.0 (4.5)	126,769	25.8 (4.1)	92,837	25.9 (4.0)	93,807	26.0 (3.9)	51,072	26.2 (3.9)	51,407	26.4 (4.0)
Total cholesterol (mmol/l)	14,075	5.69 (1.10)	34,030	5.83 (1.10)	250,332	5.81 (1.11)	88,335	5.77 (1.10)	43,479	5.79 (1.09)	36,149	5.81 (1.12)	32,083	5.83 (1.07)	24,083	5.89 (1.10)	26,203	5.90 (1.16)
Fibrinogen (μmol/l)	6,129	9.21 (2.20)	17,726	8.99 (1.87)	89,957	9.01 (2.07)	28,845	9.20 (2.05)	16,048	9.01 (2.01)	12,011	8.94 (2.02)	15,207	8.98 (2.06)	8,411	8.90 (2.13)	9,435	8.80 (2.21)
Smoking amount (pack years)	13,447	18.6 (15.1)	41,553	6.74 (10.5)	252,036	17.0 (11.8)	81,518	13.1 (10.2)	55,050	16.3 (9.5)	39,147	18.1 (9.9)	34,339	20.0 (12.4)	21,812	21.5 (14.5)	20,170	25.8 (17.4)
Self-reported general health (0-1)	17,704	0.59 (0.27)	22,366	0.60 (0.26)	382,490	0.64 (0.22)	109,540	0.64 (0.23)	90,195	0.66 (0.22)	62,584	0.67 (0.22)	64,568	0.65 (0.22)	28,343	0.62 (0.23)	27,260	0.60 (0.24)
All-cause mortality	3,777	12.9%	5,714	10.7%	40,317	6.9%	14,036	8.1%	7,479	6.0%	5,574	6.0%	5,475	5.9%	3,431	6.7%	4,322	8.4%
All cardiovascular disease	2,436	8.6%	3,763	7.3%	26,260	4.5%	8,665	5.2%	5,111	4.2%	3,682	4.0%	3,905	4.3%	2,347	4.7%	2,550	5.0%
All stroke	813	2.7%	1,473	2.7%	12,098	2.0%	4,516	2.5%	2,412	1.9%	1,485	1.6%	1,582	1.7%	1,005	1.9%	1,098	2.1%
Myocardial infarction	1,020	3.4%	1,378	2.6%	14,545	2.4%	5,458	3.1%	2,865	2.2%	1,809	1.9%	1,970	2.1%	1,172	2.3%	1,271	2.4%
Coronary disease non-MI	484	1.6%	531	1.0%	8,039	1.3%	2,686	1.5%	1,639	1.3%	1,016	11%	1,270	1.3%	695	1.3%	733	1.4%
Heart failure	461	1.6%	755	1.5%	2,748	0.5%	1,034	0.6%	492	0.4%	472	0.5%	351	0.4%	181	0.4%	218	0.4%
Death from other type of cardiovascular diease	106	0.4%	151	0.3%	1,160	0.2%	370	0.2%	192	0.2%	163	0.2%	157	0.2%	133	0.3%	145	0.3%

eTable 3: Summary of events for 83 studies, restricted to current drinkers.

Cohort abbreviation	Total participants	All-cause mortality	All cardiovascular	All stroke	Fatal stroke	Non-fatal stroke	Ischaemic stroke	Haemorrhagic stroke	Subarachnoid haemorrhages	Unclassified stroke	MI	Non-fatal MI	Fatal MI	Coronary disease excluding MI	Non-fatal coronary disease excluding MI	Fatal coronary disease excluding MI	Heart failure	Fatal cardiac dysrhythmia	Fatal hypertensive disease	Sudden death	Fatal aortic aneurysm
Case-cohort studie																					
EPIC-CVD	26036	784	12758	5507	581	4926	3293	686	353	1146	5919	4963	896	2045	1675	370	-	-	-	-	-
Nested case-control studies																					
FLETCHER	572	-	85	-	-	-	-	-	-	-	-	-	-	85	-	-	-	-	-	-	-
GLOSTRUP	313	14	63	-	-	-	-	-	-	-	61	47	14	2	2	-	-	-	-	-	-
HPFS	575	69	181	6	6	-	2	2	-	1	140	130	10	14	-	14	-	-	-	18	2
WHIHABPS	108	108	84	71	2	69	71	-	-	-	9	9	-	3	-	3	-	-	-	-	-
SUBTOTAL	1568	191	413	77	8	69	73	2	-	1	210	186	24	104	2	17	-	-	-	18	2
Clinical trials																					
AFTCAPS	2566	46	117	14	-	14	5	-	-	9	51	50	1	38	38	-	7	-	-	5	-
MRFIT	3453	239	218	18	4	14	1	-	1	15	170	142	28	12	-	12	4	5	1	-	2
PROSPER	1710	104	181	45	2	43	-	-	-	45	82	82	-	16	-	16	33	-	-	-	-
WOSCOPS	5070	149	293	50	-	50	-	-	-	50	188	188	-	47	-	47	-	-	-	-	-
SUBTOTAL	12799	538	809	127	6	121	6	-	1	119	491	462	29	113	38	75	44	5	1	5	2
Prospective cohort studies																					
UKBIOBANK	326372	6720	7469	1616	108	1508	997	214	202	181	1953	1787	166	3404	3126	278	255	4	34	-	65
ARIC	5987	1664	1365	352	30	322	273	37	18	15	361	314	47	44	-	44	542	10	25	-	7
ATENA	3483	27	21	3	-	3	1	1	1	-	12	11	1	-	-	-	-	2	2	-	1
ATTICA	1053	22	13	-	-	-	-	-	-	-	-	-	-	-	-	-	-	-	-	-	-
AUSDIAB	2996	202	36	10	5	5	2	1	1	5	16	14	2	9	3	6	-	-	-	-	1
BHS	3052	647	276	70	70	-	5	6	-	51	94	-	94	68	-	68	10	4	4	-	10
BRUN	404	142	73	29	11	18	21	8	-	-	25	14	11	6	-	6	3	-	-	-	4
BWHHS	1561	395	132	63	20	43	1	3	2	51	35	26	9	19	6	13	2	2	1	-	2
CAPS	1878	307	224	15	15	-	3	-	-	11	161	107	54	31	-	31	-	-	-	-	-
CASTEL	2443	1072	514	101	101	-	-	-	-	101	92	-	92	-	-	-	221	-	-	72	-
CHARL	142	100	24	5	-	5	-	-	-	5	12	7	5	-	-	-	6	-	-	-	-
CHS1	2286	1139	691	204	1	203	163	30	-	11	251	177	74	-	-	-	222	-	-	-	-
CHS2	209	79	52	17	-	17	15	1	-	1	17	12	5	-	-	-	17	-	-	-	-
COPEN	6552	2656	1613	470	41	429	295	56	13	94	342	342	-	615	615	-	43	4	16	9	11
DESIR	3229	63	29	12	-	12	7	3	-	2	17	17	-	-	-	-	-	-	-	-	-
DRECE	1824	107	24	5	5	-	-	2	-	3	6	-	6	7	-	7	1	-	1	-	-
DUBBO	1299	463	309	104	2	102	44	11	2	45	126	126	-	38	-	38	15	4	-	-	2
EAS	697	314	133	59	28	31	1	5	2	43	41	22	19	14	-	14	7	1	5	-	1
EPESEBOS	701	128	166	37	-	37	27	6	2	2	37	32	5	35	31	4	35	16	-	-	1
EPESEIOW	650	587	144	43	4	39	19	5	-	18	27	21	6	30	23	7	30	9	-	-	1
EPESENCA	389	241	81	27	3	24	15	3	-	9	21	19	2	14	9	5	15	4	-	-	-
EPESENHA	497	102	131	25	1	24	18	3	-	4	26	25	1	20	20	-	22	25	1	-	-
ESTHER	4531	111	285	56	-	56	-	-	-	56	33	32	1	-	-	-	196	-	-	-	-
FINNMARK	2837	113	29	9	9	-	3	1	2	3	12	-	12	4	-	4	-	1	-	-	2
FINRISK92	3444	148	321	63	7	56	37	23	1	1	51	46	5	4	-	4	193	-	1	1	1
FINRISK97	4256	118	325	48	2	46	36	10	-	2	45	40	5	4	-	4	219	-	1	-	-
FUNAGATA	214	8	15	12	1	11	8	3	-	1	3	3	-	-	-	-	-	-	-	-	-
GREPCO	500	4	-	-	-	-	-	-	-	-	-	-	-	-	-	-	-	-	-	-	-
HBS	46	30	5	1	1	-	-	-	-	1	-	-	-	4	-	4	-	-	-	-	-
HCS	2328	214	47	5	5	-	-	1	1	3	10	-	10	11	-	11	2	-	2	-	9
HIMS	5250	2017	938	288	32	256	140	47	3	88	308	235	73	169	132	37	133	6	9	-	10
HISAYAMA	864	190	123	75	3	72	50	18	6	-	25	23	2	1	-	1	-	1	1	-	3
HONOL	883	185	91	43	15	28	2	16	1	23	34	29	5	6	-	6	-	2	1	-	3
HUBRO	11498	539	124	42	42	-	6	9	2	13	28	-	28	8	-	8	7	6	6	-	11
IKNS	2701	358	188	131	12	119	69	24	5	33	30	14	16	4	-	4	18	1	-	-	2
KARELIA	41	31	28	5	1	4	1	-	-	4	13	10	3	2	-	2	8	-	-	-	-
KIHD	1805	512	535	126	14	112	86	33	2	3	319	315	4	72	69	3	2	-	2	-	5
LASA	1458	396	60	10	-	10	-	-	-	10	26	26	-	-	-	-	24	-	-	-	-
MATISS83	2004	364	251	71	6	65	20	7	1	40	60	38	22	8	2	6	38	54	7	-	-
MATISS87	1401	182	122	37	-	37	7	3	1	26	30	14	16	2	-	2	18	27	3	1	-
MATISS93	648	18	25	5	-	5	1	1	1	2	11	9	2	1	1	-	3	4	1	-	-
MCVDRFP	14655	1106	274	56	56	-	4	20	12	18	92	-	92	26	-	26	15	11	3	6	14
MESA	2388	161	85	39	-	39	33	5	-	1	30	30	-	13	-	13	-	-	-	-	-
MICOL	15563	382	116	23	23	-	4	2	-	15	53	-	53	32	-	32	-	-	-	-	1
MONICA_KORA1	757	124	85	5	5	-	-	2	-	2	55	38	17	4	-	4	9	-	-	1	2
MONICA_KORA2	2655	177	83	3	3	-	-	-	1	2	63	41	22	7	-	7	6	-	1	1	-
MONICA_KORA3	3022	378	177	30	30	-	8	8	-	13	104	81	23	21	-	21	4	2	-	7	-
MRCOLD	4689	2736	1111	340	340	-	22	27	4	200	221	-	221	281	-	281	67	29	14	-	37
NFR	2768	287	103	24	24	-	2	7	1	10	49	-	49	25	-	25	-	-	-	-	3
NHANESI	6828	1482	915	191	62	129	54	24	9	98	301	162	139	228	121	107	79	22	22	-	11
NHANESIII	3677	753	225	51	51	-	-	-	-	51	33	-	33	64	-	64	8	-	11	-	3
NPHSII	2314	325	197	53	7	46	29	5	5	14	124	113	11	1	-	1	-	-	3	10	5
NSHS	708	46	46	13	1	12	-	1	-	12	3	-	3	30	30	-	-	-	-	-	-
OPPHED	5793	225	53	16	16	-	2	5	-	9	21	-	21	4	-	4	2	5	1	-	-
OSAKA	7521	290	108	61	8	53	21	14	4	22	20	16	4	1	-	1	21	1	1	1	2
OSLO2	3824	701	164	45	45	-	6	16	3	16	42	-	42	18	-	18	13	9	6	-	8
PRHHP	1439	188	80	10	7	3	5	4	-	-	39	29	10	13	7	6	-	-	6	7	3
PRIME	7946	141	126	25	-	25	18	5	-	2	84	78	6	4	-	4	-	-	-	12	-
PROCAM	10089	423	311	37	13	24	27	6	-	4	180	162	18	30	4	26	4	1	-	37	5
QUEBEC	2113	543	414	89	4	85	-	-	-	89	253	229	24	14	-	14	6	-	-	46	-
RANCHO	1353	558	354	132	7	125	-	1	-	125	149	148	1	-	-	-	7	8	11	-	5
RS_I	3145	820	440	144	70	74	20	14	2	102	141	120	21	-	-	-	38	-	-	35	12
RS_II	1119	117	80	17	7	10	2	2	-	13	45	45	-	-	-	-	3	-	-	9	1
RS_III	2258	28	6	1	1	-	-	-	-	-	-	-	-	1	-	1	-	-	-	1	1
SHHEC	7919	417	410	88	5	83	21	11	12	41	208	168	40	100	86	14	1	1	1	2	4
SHIP	1746	3	48	23	-	23	-	-	-	23	25	25	-	-	-	-	-	-	-	-	-
TOYAMA	2480	68	57	30	-	30	12	13	5	-	21	21	-	-	-	-	3	-	-	-	-
TROMS	1134	26	9	-	-	-	-	-	-	-	5	-	5	1	-	1	-	1	1	-	1
TROMSØ	10024	862	592	244	9	235	178	29	23	11	301	272	29	12	-	12	3	2	4	10	6
ULSAM	703	326	258	79	6	73	56	11	3	7	73	64	9	27	12	15	64	-	3	-	4
WCWC	2310	222	12	-	-	-	-	-	-	-	12	12	-	-	-	-	-	-	-	-	-
WHITEI	3099	1606	599	181	181	-	19	11	3	96	104	-	104	140	-	140	36	19	6	-	51
WHITEII	8776	426	370	7	7	-	1	1	1	4	323	297	26	24	-	24	-	-	1	-	3
ZUTE	281	142	98	36	-	36	-	-	-	36	41	40	1	2	-	2	8	1	-	1	7
SUBTOTAL	559509	38804	25038	6387	1583	4804	2917	835	357	1997	7925	6098	1827	5777	4297	1480	2704	299	218	269	341
TOTAL	599912	40317	39018	12098	2178	9920	6289	1523	711	3263	14545	11709	2776	8039	6012	1942	2748	304	219	292	345
TOTAL events / participants (excluding studies with fewer than 5 events for that particular outcome)		40310 / *584728	39018 / 599412	12090 / 585588	2142 / 532204	9910 / 491050	6256 / 491204	1482 / 505948	663 / 412732	3215 / 527729	14539 / 594561	11706 / 515377	2748 / 538117	7990 / 523548	6000 / 389976	1889 / 510147	2711 / 447436	261 / 71682	178 / 383269	283 / 68002	289 / 423145

*All-cause mortality events derived only from the 13,670 participants in the random sub-cohort of EPIC-CVD, rather than from the much larger number of participants in the full prospective EPIC study

eTable 4. Comparison of baseline characteristics of individuals used in main analysis versus individuals with repeat measures of alcohol consumption or measures of lifetime alcohol consumption from the contributing data sources.

	**ERFC**				**EPIC-CVD**				**UK Biobank**			
	**All participants**		**Participants with repeat measures of alcohol consumption**		**All participants**		**Participants with measured lifetime alcohol consumption**		**All participants**		**Participants with repeat measures of alcohol consumption**	
Number of studies/centres	81 studies		35 studies		22 centres		17 centres		1 study		1 study	
Current drinkers at baseline	247,504		38,472		26,036		18,779		326,372		13,760	
Alcohol consumption categories at baseline	n (%)	Mean baseline alcohol consumption g/wk	n (%)	Mean baseline / resurvey alcohol consumption g/wk	n (%)	Mean baseline alcohol consumption g/wk	n (%)	Mean baseline / lifetime alcohol consumption g/wk	n (%)	Mean baseline alcohol consumption g/wk	n (%)	Mean baseline / resurvey alcohol consumption g/wk
>0-≤25g/wk,	53,418 (21.6%)	10	5,734 (14.9%)	11 / 38	7,906 (30.4%)	10	5,247 (27.9%)	10 / 33	39,641 (12.2%)	14	1,320 (9.6%)	15 / 23
>25-≤50g/wk,	33,953 (13.7%)	36	4,335 (11.3%)	37 / 58	3,704 (14.2%)	37	2,367 (12.6%)	37 / 58	39,334 (12.1%)	40	1,663 (12.1%)	40 / 43
>50-≤75g/wk,	26,656 (10.8%)	62	3,591 (9.3%)	62 / 83	2,748 (10.6%)	62	1,867 (9.9%)	62 / 80	42,907 (13.2%)	64	1,864 (13.6%)	64 / 64
>75-≤100g/wk,	16,557 (6.7%)	86	2,936 (7.6%)	86 / 103	2,446 (9.4%)	86	1,813 (9.7%)	86 / 91	36,780 (11.3%)	87	1,645 (12.0%)	87 / 82
>100-≤150g/wk	36,236 (14.6%)	124	5,617 (14.6%)	127 / 129	2,602 (10.0%)	123	1,883 (10.0%)	123 / 127	55,815 (17.1%)	124	2,551 (18.5%)	124 / 112
>150-≤250g/wk	31,645 (12.8%)	195	7,175 (18.7%)	191 / 172	3,090 (11.9%)	193	2,447 (13.0%)	193 / 182	60,025 (18.4%)	194	2,633 (19.1%)	194 / 171
>250-≤350g/wk	23,607 (9.5%)	308	4,289 (11.2%)	309 / 249	1,744 (6.7%)	293	1,507 (8.0%)	294 / 249	26,669 (8.2%)	292	1,131 (8.2%)	292 / 245
≥350g/wk	25,432 (10.3%)	568	4,795 (12.5%)	521 / 345	1,796 (6.9%)	505	1,648 (8.8%)	507 / 403	25,201 (7.7%)	515	953 (6.9%)	499 / 388
Age in years at baseline, mean (SD)	57.1 (8.7)		55.3 (8.3)		55.0 (9.2)		54.9 (8.7)		56.5 (8.0)		57.3 (7.3)	
Sex, n (%)												
Male	162,685 (65.7%)		27,701 (72.0%)		13,508 (51.9%)		9,559 (51.1%)		157,809 (48.4%)		7,060 (51.3)	
Female	84,819 (34.3%)		10,771 (28.0%)		12,528 (48.1%)		9,180 (48.9%)		168,563 (51.6%)		6,700 (48.7)	
Smoking status, n (%)												
Not current	161,037 (65.1%)		25,319 (65.8%)		17,608 (67.6%)		12,693 (67.6%)		293,182 (89.8%)		12,918 (93.9%)	
Current	86,467 (34.9%)		13,153 (34.2%)		8,428 (32.4%)		6,086 (32.4%)		33,190 (10.2%)		842 (6.1%)	
History of diabetes, n(%)												
No	237,685 (96.0%)		36,936 (96.0%)		24,875 (95.5%)		17,889 (95.3%)		315,090 (96.5%)		13,334 (96.9%)	
Yes	9,819 (4.0%)		1,536 (4.0%)		1,161 (4.5%)		890 (4.7%)		11,282 (3.5%)		426 (3.1%)	
BMI in kg/m^2, mean (SD)	26.1 (3.8)		26.0 (3.5)		26.4 (4.1)		26.7 (4.2)		27.0 (4.4)		26.6 (4.2)	
HDL-C in mmol/l, mean (SD)	1.40 (0.41)		1.41 (0.40)		1.40 (0.42)		1.41 (0.43)		not available at time of analysis		not available at time of analysis	
Total cholesterol in mmol/l, mean (SD)	5.80 (1.17)		5.77 (1.05)		6.11 (1.16)		0.12 (1.16)		not available at time of analysis		not available at time of analysis	
Systolic blood pressure in mmHg, mean (SD)	136.5 (19.0)		134.4 (17.5)		138.4 (21.3)		137.9 (21.1)		137.9 (18.5)		137.5 (17.8)	

SD = standard deviation, BMI = body-mass index, HDL-C = high density lipoprotein cholesterol.

eTable 5. Hazard ratios for cardiovascular outcomes amongst current drinkers, without and with adjustment for usual or baseline levels of potential confounders, mediators and proxies thereof.

**Level of adjustment**	**HR (95% CI) per 100 grams/week higher usual alcohol consumption**				
	All stroke	Myocardial infarction	Coronary disease excluding myocardial infarction	Heart failure	Deaths from other types of cardiovascular disease
No. of cohorts / events	50 / 6939	54 / 9,183	32 / 3,399	24 / 1,782	29 / 521
Basic adjustment*	1.16 (1.10, 1.22)	0.95 (0.89, 1.00)	1.06 (0.97, 1.16)	1.08 (1.00, 1.16)	1.20 (1.10, 1.31)
+ usual LDL cholesterol	1.17 (1.11, 1.23)	0.96 (0.92, 1.01)	1.07 (0.98, 1.17)	1.09 (1.00, 1.18)	1.21 (1.09, 1.33)
No. of cohorts / events	61 / 7,891	66 / 10,755	39 / 3,885	32 / 2,090	38 / 826
Basic adjustment*	1.16 (1.10, 1.21)	0.95 (0.91, 1.00)	1.07 (1.00, 1.14)	1.12 (1.01, 1.23)	1.15 (1.02, 1.27)
+ usual total cholesterol	1.15 (1.10, 1.20)	0.93 (0.90, 0.97)	1.06 (0.99, 1.14)	1.13 (1.03, 1.24)	1.15 (1.04, 1.28)
No. of cohorts / events	31 / 2,236	34 / 3,007	22 / 1,236	16 / 1,099	18 / 303
Basic adjustment*	1.14 (1.07, 1.21)	0.94 (0.89, 1.00)	1.12 (0.99, 1.26)	1.16 (0.99, 1.35)	1.20 (1.10, 1.32)
+ usual fibrinogen	1.13 (1.08, 1.18)	0.97 (0.92, 1.03)	1.12 (1.01, 1.25)	1.25 (1.07, 1.46)	1.24 (1.10, 1.40)
No. of cohorts / events	53 / 2,649	59 / 3,241	29 / 1,809	26 / 1,211	30 / 453
Basic adjustment*	1.10 (1.05, 1.15)	0.93 (0.88, 0.97)	1.05 (0.98, 1.12)	1.05 (1.00, 1.11)	1.20 (1.10, 1.31)
+ baseline smoking amount	1.09 (1.04, 1.15)	0.92 (0.88, 0.97)	1.03 (0.95, 1.10)	1.02 (0.97, 1.08)	1.19 (1.09, 1.30)
No. of cohorts / events	30 / 8,055	32 / 9,238	21 / 5,795	18 / 1,570	18 / 420
Basic adjustment*	1.13 (1.09, 1.17)	0.92 (0.86, 0.99)	1.05 (0.93, 1.20)	1.08 (0.99, 1.17)	1.22 (1.07, 1.38)
+ baseline education level and occupation	1.13 (1.09, 1.18)	0.92 (0.87, 0.99)	1.05 (0.92, 1.19)	1.07 (0.98, 1.17)	1.22 (1.07, 1.40)
No. of cohorts / events	1 / 4,916	1 / 5,291	1 / 2,006	-	-
Basic adjustment*	1.17 (1.11, 1.21)	0.89 (0.85, 0.93)	0.98 (0.90, 1.07)		
+ baseline physical activity	1.16 (1.11, 1.21)	0.89 (0.85, 0.93)	0.98 (0.90, 1.07)		
No. of cohorts / events	24 / 2,717	24 / 3,006	24 / 4,427	24 / 1,071	24 / 296
Basic adjustment*	1.13 (1.10, 1.16)	0.95 (0.91, 0.98)	1.01 (0.98, 1.04)	1.14 (1.08, 1.20)	1.16 (1.08, 1.24)
+ baseline self-reported general heath	1.12 (1.09, 1.16)	0.94 (0.91, 0.98)	1.00 (0.97, 1.03)	1.13 (1.06, 1.19)	1.15 (1.07, 1.24)
No. of cohorts / events	1 / 1,608	1 / 1,945	1 / 3,370	1 / 254	1 / 103
Basic adjustment*	1.11 (1.07, 1.15)	0.94 (0.90, 0.98)	1.00 (0.97, 1.04)	1.07 (0.97, 1.19)	1.17 (1.09, 1.26)
+ baseline red meat consumption[1]	1.11 (1.07, 1.15)	0.93 (0.89, 0.97)	1.00 (0.97, 1.03)	1.05 (0.95, 1.16)	1.17 (1.08, 1.27)
No. of cohorts / events	57 / 4,114	57 / 4,717	35 / 2,175	33 / 1,680	37 / 842
Basic adjustment*	1.17 (1.11, 1.23)	0.92 (0.87, 0.97)	1.09 (1.03, 1.15)	1.13 (1.06, 1.21)	1.17 (1.01, 1.37)
+ baseline anti-hypertensive drug use[2]	1.17 (1.11, 1.23)	0.92 (0.87, 0.97)	1.08 (1.02, 1.15)	1.14 (1.06, 1.22)	1.17 (1.00, 1.36)

Analyses restricted to individuals with basic adjustment variables plus the additional variable. Studies with fewer than five events were excluded from the analysis of each outcome. *Basic adjustment includes age, smoking and history of diabetes, and stratified by sex and EPIC centre. [1]Adjustment includes separate variables for pork, beef and lamb consumption. [2]Adjustment includes systolic blood pressure, anti-hypertinsive drug use and their interaction.

eTable 6. Hazard ratios for death from lung cancer and digestive related cancer outcomes per 100 grams/wk higher usual alcohol consumption amongst current drinkers, without and with adjustment for usual or baseline levels of potential confounders, mediators and proxies thereof.

Level of adjustment	Deaths from lung cancer		Death from digestive related cancer	
	No. of cohorts / events	HR (95% CI)	No. of cohorts / events	HR (95% CI)
Basic adjustment*	49 / 2,530	1.18 (1.10, 1.27)	55 / 3,747	1.17 (1.12, 1.24)
+ usual systolic blood pressure		1.18 (1.10, 1.26)		1.17 (1.11, 1.22)
Basic adjustment*	39 / 1,356	1.17 (1.07, 1.29)	45 / 1,768	1.19 (1.11, 1.26)
+ usual HDL cholesterol		1.20 (1.08, 1.33)		1.18 (1.12, 1.25)
Basic adjustment*	49 / 2,490	1.18 (1.10, 1.26)	53 / 3,686	1.16 (1.10, 1.24)
+ usual body mass index		1.18 (1.10, 1.26)		1.16 (1.10, 1.24)
Basic adjustment*	45 / 1,561	1.17 (1.08, 1.27)	49 / 2,056	1.17 (1.10, 1.24)
+ usual total cholesterol		1.17 (1.07, 1.27)		1.17 (1.10, 1.24)
Basic adjustment*	19 / 1,926	1.13 (1.02, 1.24)	19 / 1,922	1.17 (1.09, 1.26)
+ baseline education and occupation		1.11 (1.01, 1.12)		1.15 (1.08, 1.22)
Basic adjustment*	24 / 838	1.09 (1.04, 1.15)	43 / 1,517	1.15 (1.08, 1.22)
+ baseline smoking amount		1.04 (0.98, 1.09)		1.14 (1.07, 1.21)

Analyses restricted to individuals with basic adjustment variables plus the additional variable. Studies with fewer than five events were excluded from the analysis of each outcome. *Basic adjustment includes age, smoking status and history of diabetes, and stratified by sex and EPIC centre. Digestive cancers were defined as tumours of the liver, colorectum, stomach, pancreas and oesophagus.

eTable 7: Sex-specific hazard ratios for major cardiovascular outcomes per 100 grams/week increase in usual alcohol consumption amongst current drinkers.

Description of sensitivity analyses	Outcome	No. of events	Hazard Ratio (95% CI) per 100 grams/week increase	I^2 (95% CI)
**Restricted to men**	All stroke	7,280	1.15 (1.10, 1.19)	17% (0%, 39%)
	Myocardial infarction	11,068	0.95 (0.93, 0.98)	5% (0%, 29%)
	Coronary disease excluding myocardial infarction	5,591	1.05 (1.00, 1.11)	23% (0%, 47%)
	Heart failure	1,663	1.10 (1.05, 1.15)	1% (0%, 39%)
	Deaths from other types of cardiovascular disease	795	1.17 (1.06, 1.29)	30% (9%, 53%)
**Restricted to women**	All stroke	4,704	1.09 (1.01, 1.18)	3% (0%, 29%)
	Myocardial infarction	3,407	0.87 (0.75, 1.01)	28% (0%, 52%)
	Coronary disease excluding myocardial infarction	2,349	1.07 (0.86, 1.33)	54% (23%, 72%)
	Heart failure	1,010	0.94 (0.82, 1.08)	0% (0%, 45%)
	Deaths from other types of cardiovascular disease	287	1.45 (1.10, 1.92)	19% (0%, 53%)

Studies with fewer than five events were excluded from the analysis of each outcome. *Adjusted for age, smoking and history of diabetes, and stratified by sex and EPIC centre.

eTable 8: Sensitivity analyses: Hazard ratios for major cardiovascular outcomes per 100 grams/week increase in usual alcohol consumption amongst current drinkers.

Description of sensitivity analyses	Outcome	No. of events	Hazard Ratio (95% CI) per 100 grams/week increase	I^2 (95% CI)
**Principal analysis on all individuals**	All stroke	12,090	1.14 (1.10, 1.17)	12% (0%, 35%)
	Myocardial infarction	14,539	0.94 (0.91, 0.97)	12% (0%, 35%)
	Coronary disease excluding MI	7,990	1.06 (1.00, 1.11)	26% (0%, 49%)
	Heart failure	2,711	1.09 (1.03, 1.15)	4% (0%, 31%)
	Deaths from other types of cardiovascular disease	1,121	1.18 (1.07, 1.30)	33% (2%, 53%)
**Excluding first five years of follow-up**	All stroke	8,005	1.14 (1.10, 1.18)	6% (0%, 32%)
	Myocardial infarction	8,880	0.94 (0.91, 0.97)	0% (0%, 29%)
	Coronary disease excluding MI	3,989	1.06 (1.02, 1.10)	0% (0%, 37%)
	Heart failure	1,821	1.09 (1.04, 1.14)	0% (0%, 38%)
	Deaths from other types of cardiovascular disease	808	1.17 (1.07, 1.28)	6% (0%, 36%)
**Excluding current smokers**	All stroke	8,185	1.15 (1.12, 1.18)	0% (0%, 30%)
	Myocardial infarction	8,880	0.95 (0.93, 0.98)	0% (0%, 28%)
	Coronary disease excluding MI	5,994	1.07 (0.98, 1.17)	40% (12%, 59%)
	Heart failure	1,926	1.14 (1.06, 1.23)	14% (0%, 44%)
	Deaths from other types of cardiovascular disease	679	1.20 (1.09, 1.32)	6% (0%, 35%)
**Excluding people with a history of diabetes**	All stroke	11,089	1.13 (1.10, 1.17)	8% (0%, 33%)
	Myocardial infarction	13,418	0.95 (0.91, 0.98)	25% (0%, 44%)
	Coronary disease excluding MI	7,365	1.06 (1.01, 1.11)	23% (0%, 47%)
	Heart failure	2,351	1.13 (1.05, 1.21)	15% (0%, 44%)
	Deaths from other types of cardiovascular disease	1,022	1.17 (1.06, 1.30)	36% (7%, 56%)
**Excluding people with a history of cancer**	All stroke	6,528	1.10 (1.07, 1.12)	0% (0%, 50%)
	Myocardial infarction	7,306	0.94 (0.90, 0.98)	11% (0%, 48%)
	Coronary disease excluding MI	4,744	1.10 (0.98, 1.24)	64% (37%, 79%)
	Heart failure	1,145	1.05 (1.01, 1.10)	0% (0%, 57%)
	Deaths from other types of cardiovascular disease	379	1.21 (1.07, 1.36)	31% (0%, 64%)

Studies with fewer than five events were excluded from the analysis of each outcome. *Adjusted for age, smoking and history of diabetes, and stratified by sex and EPIC centre. MI: Myocardial infarction.

eTable 9: Baseline characteristics by frequency of baseline alcohol consumption

Baseline characteristic	Drinks ≤ 2 days per week		Drinks >2 days per week	
	n	Mean (SD) or %	n	Mean (SD) or %
Age in years	194,346	57.0 (8.9)	244,903	58.0 (8.2)
Sex	194,346		244,903	
Male	89,157	45.9%	143,471	58.6%
Female	105,189	54.1%	101,432	41.4%
Ethnicity	161,710		207,898	
White	152,516	94.3%	201,651	97.0%
Non-white	9,194	5.7%	6,247	3.0%
Smoking status	194,346		244,903	
Not current	164,285	84.5%	204,092	83.3%
Current	30,061	15.5%	40,811	16.7%
Level of education	184,511		223,938	
No schooling/Primary	4,789	2.6%	4,355	1.9%
Secondary	81,783	44.3%	79,879	35.7%
Vocational/University	97,939	53.1%	139,704	62.4%
Occupation	163,956		214,731	
Not working	58,453	35.7%	80,291	37.4%
Manual	20,372	12.4%	22,457	10.5%
Office	71,846	43.8%	97,588	45.5%
Other	13,285	8.1%	14,395	6.7%
History of diabetes	194,346		244,903	
No history	186,451	95.9%	237,473	97.0%
Definite diabetic	7,895	4.1%	7,430	3.0%
Usual total household income before tax	118,863		164,772	
Less than £18,000	25,335	21.3%	23,749	14.4%
£18,000 to £30,999	30,965	26.0%	38,241	23.2%
£31,000 to £51,999	32,899	27.7%	46,141	28.0%
£52,000 to £100,000	24,416	20.5%	42,983	26.1%
Greater than £100,000	5,248	4.4%	13,658	8.3%
Townsend deprivation index	139,416	-1.36 (3.0)	186,555	-1.71 (2.8)
Systolic blood pressure (mmHg)	192,672	135.5 (18.6)	243,256	138.0 (18.6)
HDL-C (mmol/l)	45,830	1.33 (0.37)	46,369	1.42 (0.39)
BMI (kg/m^2)	190,908	26.4 (4.6)	242,299	26.2 (4.0)
Total cholesterol (mmol/l)	50,430	5.80 (1.11)	51,966	5.88 (1.12)
Fibrinogen (μmol/l)	13,162	9.14 (2.10)	18,627	8.80 (2.23)
Smoking amount	85,184	14.3 (6.3)	85,179	20.2 (8.5)
Self-reported general health (0-1)	170,928	0.64 (0.23)	204,404	0.67 (0.22)
Alcohol consumption (g/wk)	194,346	49.1 (59.4)	244,903	181.1 (156.6)
Wine consumption (g/wk)	157,209	23.8 (33.0)	202,332	104.4 (95.9)
Beer consumption (g/wk)	157,032	26.3 (54.1)	202,777	94.2 (135.9)
Spirits consumption (g/wk)	154,814	16.8 (25.6)	201,022	52.4 (56.2)

SD = standard deviation, BMI = body mass index, HDL-C = high density lipoprotein cholesterol.

eTable 10: Baseline characteristics by type of baseline alcohol predominantly consumed*

**Baseline characteristic**	**Predominantly wine drinkers**		**Predominantly beer drinkers**		**Predominantly spirit drinkers**	
	n	Mean (SD) or %	n	Mean (SD) or %	n	Mean (SD) or %
Age in years	203,900	58.0 (8.1)	106,464	56.0 (8.3)	120,069	57.0 (8.2)
Sex	203,900		106,464		120,069	
Male	78,360	38.4%	86,039	80.8%	55,924	46.6%
Female	125,540	61.6%	20,425	19.2%	64,145	53.4%
Ethnicity	189,411		92,549		102,642	
White	183,828	97.1%	89,832	97.1%	98,383	95.9%
Non-white	5,583	3.0%	2,717	2.9%	4,259	4.1%
Smoking status	203,900		106,464		120,069	
Not current	180,169	88.4%	85,087	79.9%	94,955	79.1%
Current	23,731	11.6%	21,377	20.1%	25,114	20.9%
Level of education	195,833		100,048		112,894	
No schooling/Primary	15,820	8.1%	5,749	5.8%	10,483	9.3%
Secondary	59,631	30.5%	40,335	40.3%	45,623	40.4%
Vocational/University	120,382	61.5%	53,964	53.9%	56,788	50.3%
Occupation	182,414		96,134		102,561	
Not working	69,651	38.2%	30,092	31.3%	41,597	40.6%
Manual	9,440	5.2%	17,132	17.8%	9,687	9.5%
Office	84,116	46.1%	38,525	40.1%	39,127	38.2%
Other	19,207	10.5%	10,385	10.8%	12,150	11.9%
History of diabetes	203,900		106,464		120,069	
No history	197,875	97.0%	102,097	95.9%	115,272	96.0%
Definite diabetic	6,025	3.0%	4,367	4.1%	4,797	4.0%
Usual total household income before tax	141,379		71,216		69,144	
Less than £18,000	19,309	13.7%	14,677	20.6%	14,392	20.8%
£18,000 to £30,999	32,276	22.8%	17,912	25.2%	18,451	26.7%
£31,000 to £51,999	39,569	28.0%	20,330	28.6%	18,735	27.1%
£52,000 to £100,000	37,990	26.9%	15,431	21.7%	13,803	20.0%
Greater than £100,000	12,235	8.7%	2,866	4.0%	3,763	5.4%
Townsend deprivation index	161,484	-1.83 (2.75)	80,645	-1.23 (3.06)	81,049	-1.42 (3.00)
Systolic blood pressure (mmHg)	201,083	133.5 (18.9)	105,227	134.4 (17.9)	118,057	135.6 (18.8)
HDL-C (mmol/l)	36,838	1.41 (0.39)	20,238	1.32 (0.36)	32,713	1.38 (0.39)
BMI (kg/m^2)	200,656	26.3 (4.2)	105,454	26.1 (4.2)	117,864	26.4 (4.4)
Total cholesterol (mmol/l)	40,035	5.70 (1.16)	23,720	5.62 (1.13)	36,126	5.79 (1.15)
Fibrinogen (µmol/l)	4,314	9.35 (1.94)	4,664	9.61 (2.11)	7,298	9.48 (1.94)
Smoking amount	90,512	11.4 (7.8)	39,733	17.9 (8.4)	46,883	18.3 (11.3)
Self-reported general health (0-1)	165,686	0.63 (0.23)	84,686	0.62 (0.23)	86,272	0.64 (0.23)
Alcohol consumption (g/wk)	203,900	138 (132)	106,464	153 (171)	120,069	191 (161)

SD = standard deviation, BMI = body mass index, HDL-C = high density lipoprotein cholesterol.
* Type of alcohol predominantly consumed was determined from the maximum baseline consumption grams/week for each alcohol type.

eFigure 1: Flow diagram of study selection process in current analysis

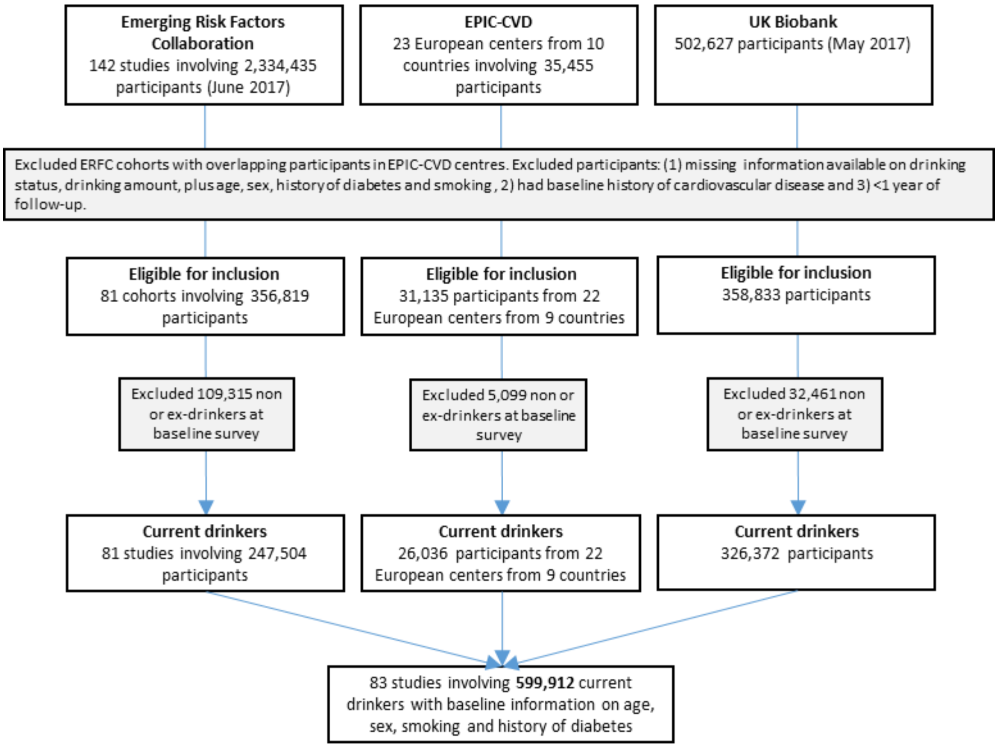

eFigure 2: Box plots of baseline alcohol consumption amongst 599,912 current drinkers from 83 studies by decade of first baseline survey.

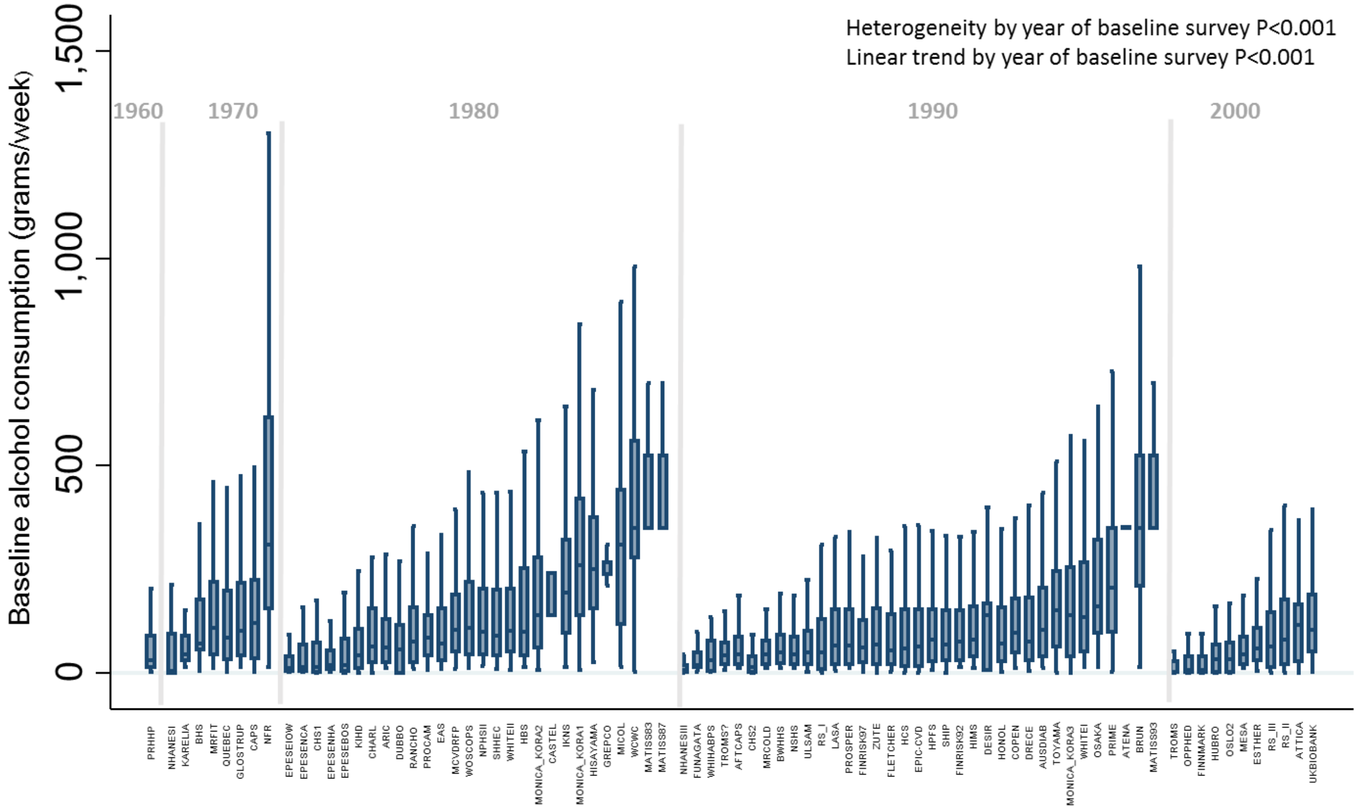

eFigure 3a: Cross-sectional associations between baseline alcohol consumption and continuous baseline characteristics.

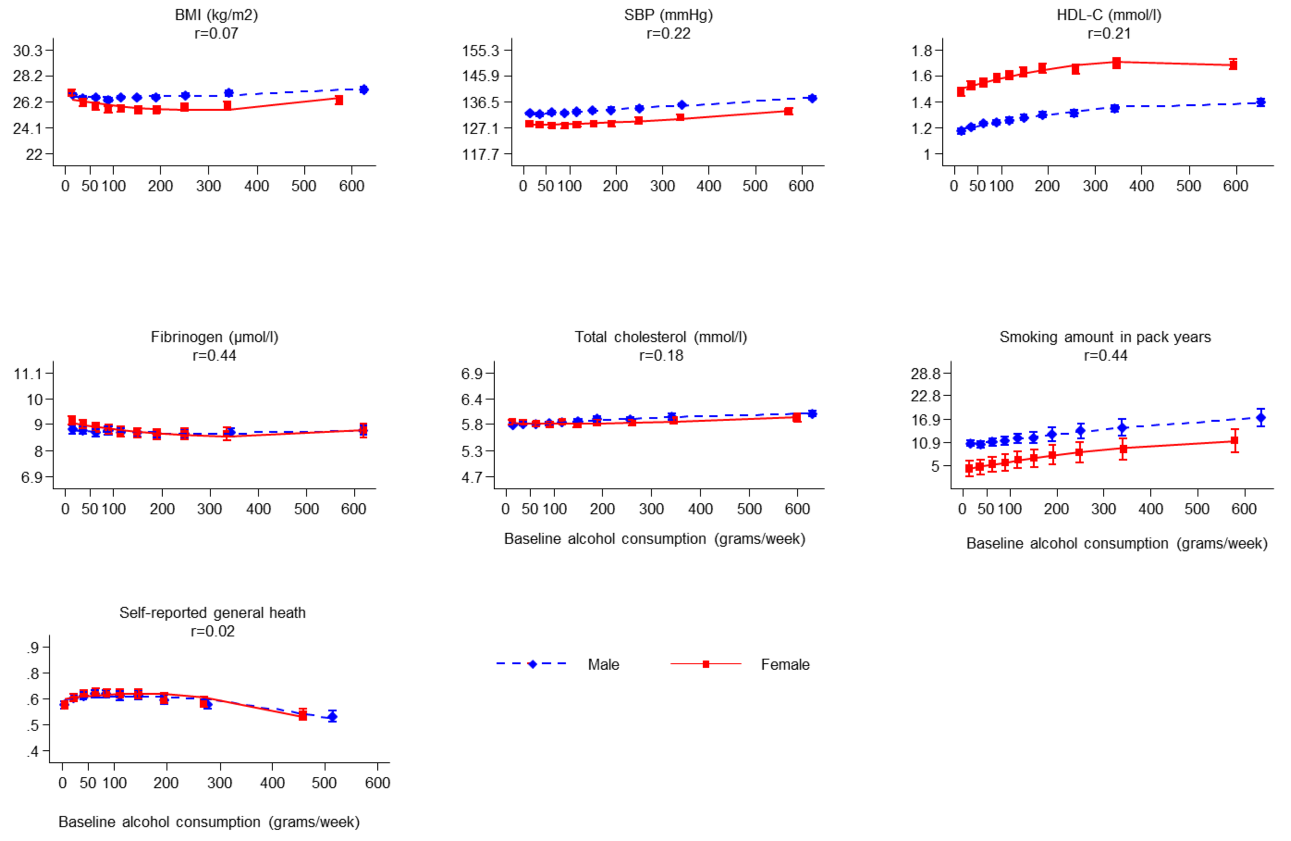

Response means are adjusted to age 50 year and plotted at deciles of baseline alcohol consumption. Red squares and solid lines represent associatons for females; blue squares and dashed lines represent associatons for males. The r values represent the age and sex adjusted partial correlation coefficient between continuous baseline characteristics and alcohol consumption in males and females combined. The Y-axis is labelled at the mean and +/- two standard deviations of the baseline characteristic of interest. BMI: Body-mass index, SBP: systolic blood pressure, HDL-C: high density lipoprotein cholesterol. Vertical lines represent 95% CIs.

eFigure 3b: Cross-sectional associations between baseline consumption and categorical baseline characteristics.

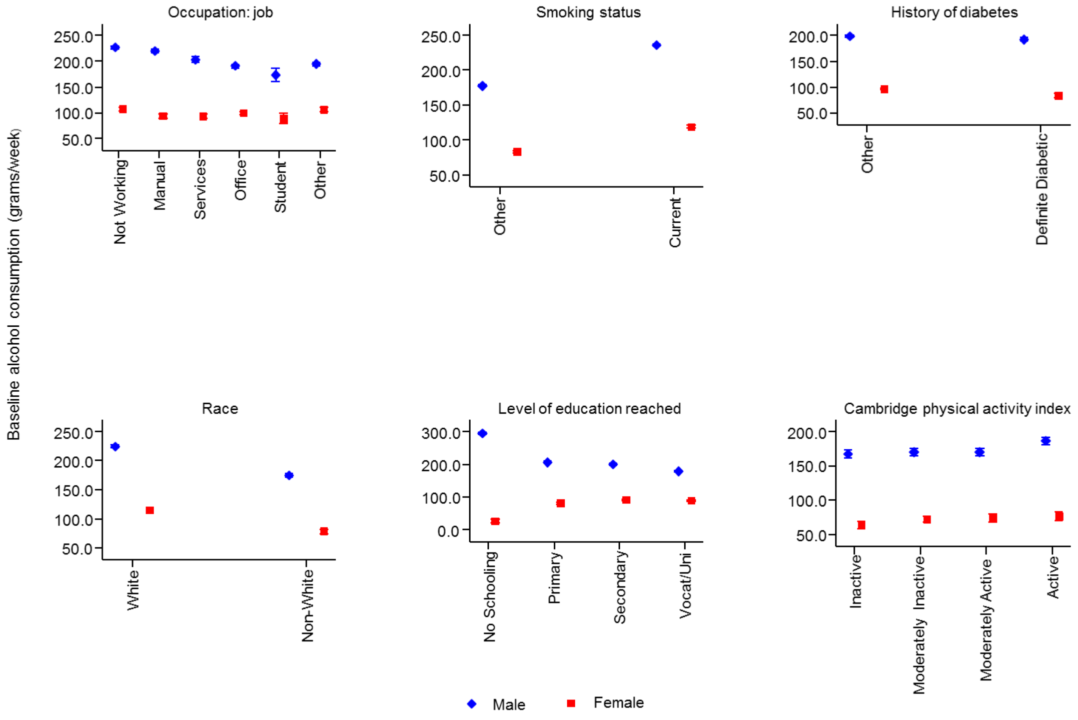

Response means are adjusted to age 50 years. Red squares represent associatons for females; blue squares represent associatons for males. Vertical lines represent 95% CIs.

eFigure 4: Shape of association of baseline alcohol consumption with all-cause mortality and all cardiovascular disease amongst current drinkers.

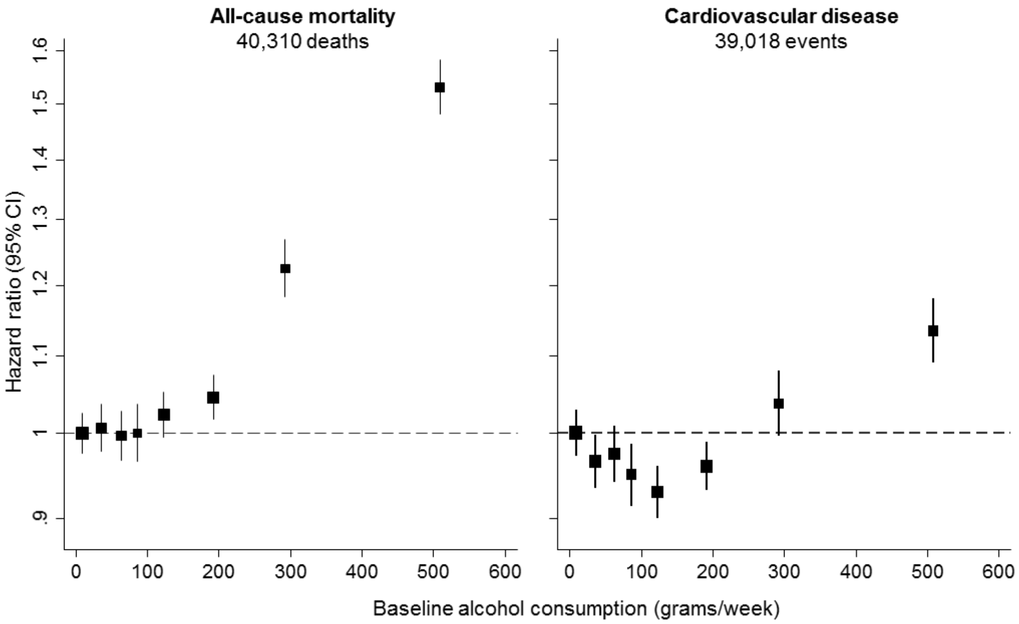

Adjusted for age, smoking and history of diabetes, and stratified by sex and EPIC centre. Studies with fewer than five events of any outcome were excluded from the analysis of that outcome. The sizes of the boxes are proportional to the inverse of the variance of the log-transformed hazard ratios. The reference category is the lowest alcohol consumption category (baseline consumption >0 and ≤25g/week). HRs are plotted against the mean baseline alcohol consumption in each category. Vertical lines represent 95% CIs. The best-fitting fractional polynomial Cox models on the log scale were: all-cause mortality, non-linear (ie, powers 0·5 and 1); and cardiovascular disease, non-linear (ie, powers 0 and 0).

eFigure 5. Shape of association of usual alcohol consumption with all-cause mortality for females and males.

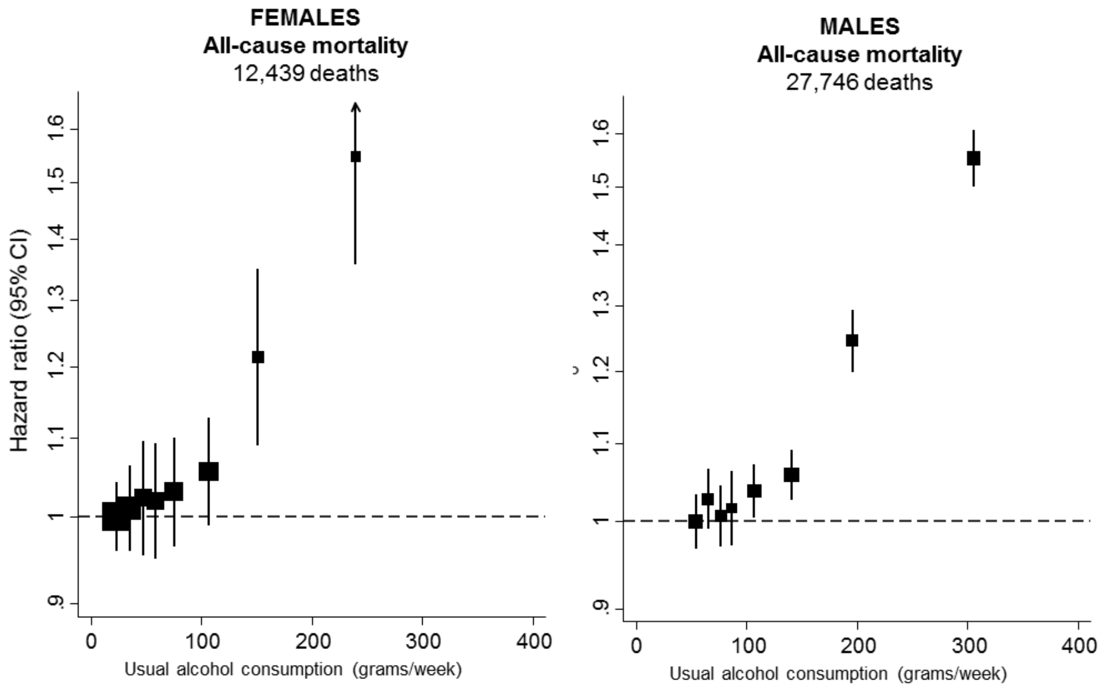

Adjusted for age, smoking and history of diabetes, and stratified by sex and EPIC centre. The reference category is the lowest alcohol consumption category (baseline consumption >0 and ≤25g/week). HRs are plotted against the mean usual alcohol consumption in each category. The sizes of the boxes are proportional to the inverse of the variance of the log-transformed hazard ratios. Vertical lines represent 95% CIs.

eFigure 6. Shape of association of usual alcohol consumption with all-cause mortality by age-specific groups.

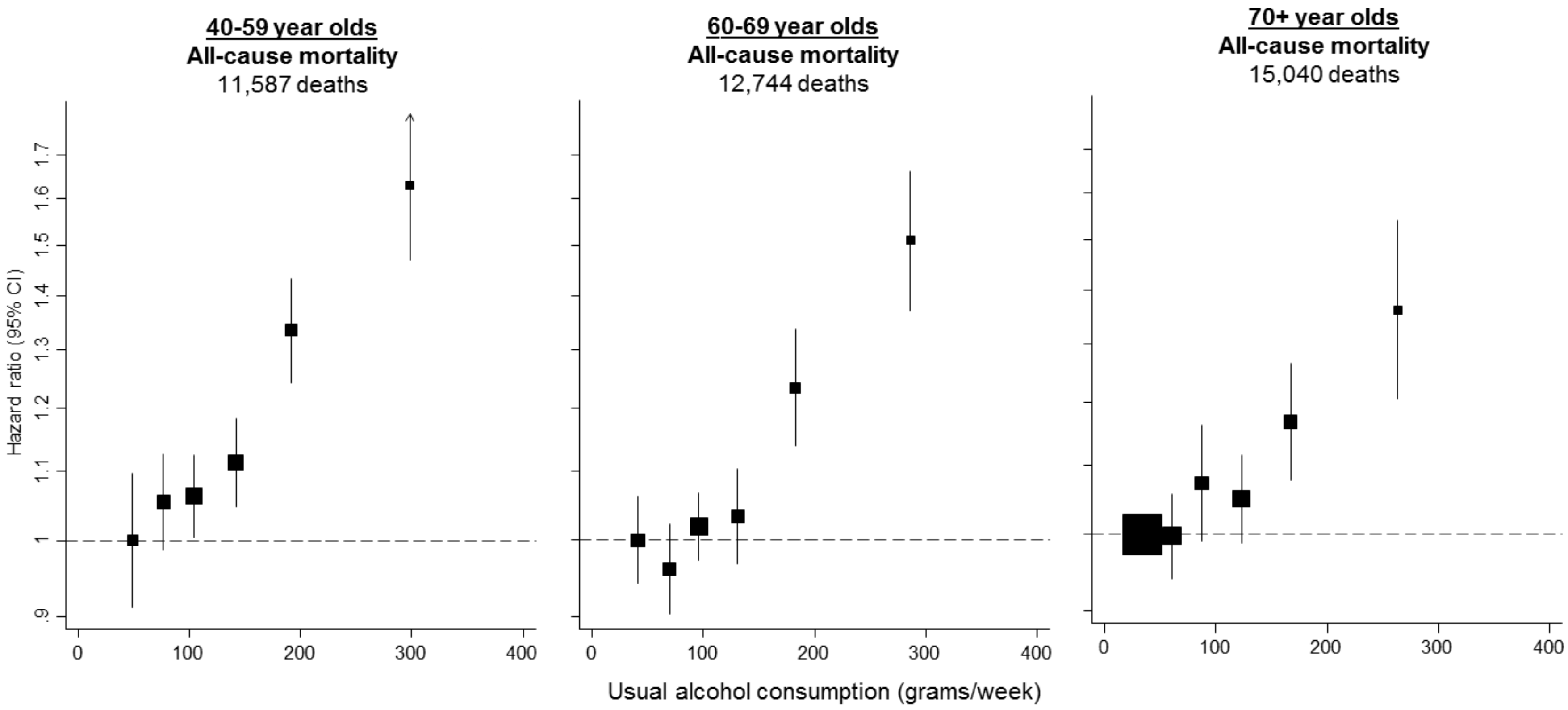

Adjusted for age, smoking and history of diabetes, and stratified by sex and EPIC centre. Baseline alcohol consumption categories amongst current drinkers were >0-≤50 grams/week, >50-≤100 grams/week, >100-≤150 grams/week, >150-≤250 grams/week, >250-≤350 grams/week and >350 grams/week. The reference category is the lowest baseline alcohol consumption category (>0 and ≤50g/week). HRs are plotted against the mean usual alcohol consumption in each category.The sizes of the boxes are proportional to the inverse of the variance of the log-transformed hazard ratios. Vertical lines represent 95% CIs.

eFigure 7. Shapes of associations of usual alcohol consumption with fatal and non-fatal major cardiovascular causes.

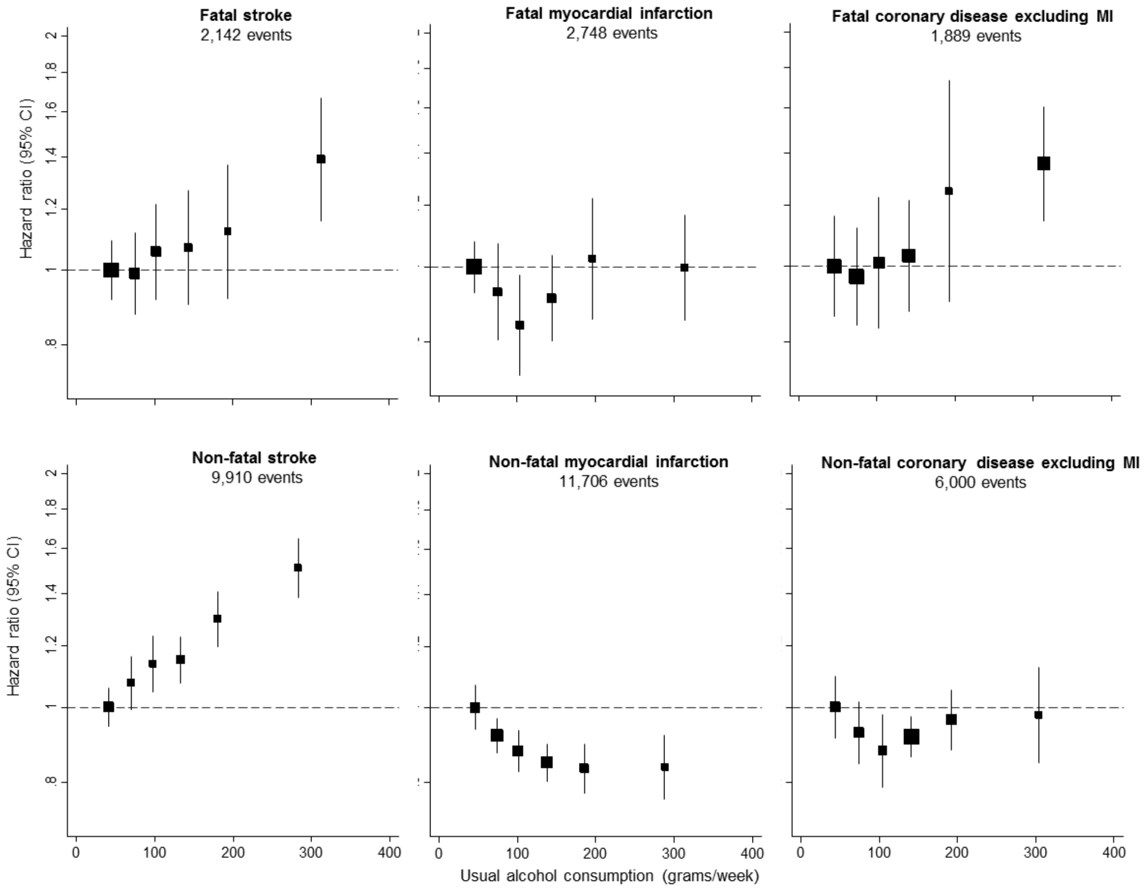

Adjusted for age, smoking and history of diabetes, and stratified by sex and EPIC centre. Alcohol consumption categories amongst current drinkers were >0-≤50 grams/week, >50-≤100 grams/week, >100-≤150 grams/week, >150-≤250 grams/week, >250-≤350 grams/week and >350 grams/week. The reference category is the lowest baseline alcohol consumption category (>0 and ≤50g/week). HRs are plotted against the mean usual alcohol consumption in each category. Studies with fewer than five events of any outcome were excluded from the analysis of that outcome. The sizes of the boxes are proportional to the inverse of the variance of the log-transformed hazard ratios. Vertical lines represent 95% CIs.

eFigure 8. Shapes of associations of usual alcohol consumption with type of stroke.

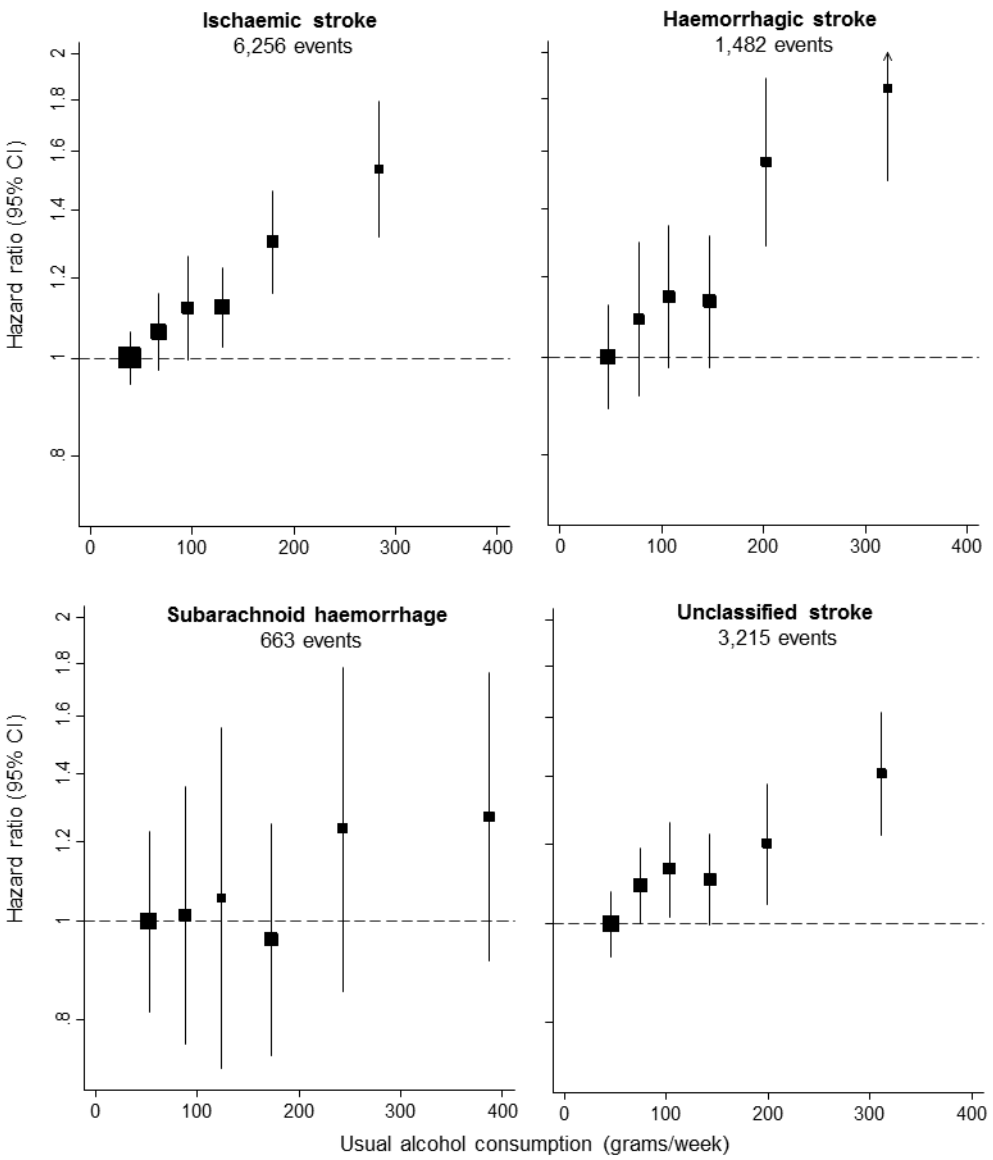

Adjusted for age, smoking and history of diabetes, and stratified by sex and EPIC centre. Baseline alcohol consumption categories amongst current drinkers were >0-≤50 grams/week, >50-≤100 grams/week, >100-≤150 grams/week, >150-≤250 grams/week, >250-≤350 grams/week and >350 grams/week. The reference category is the lowest baseline alcohol consumption category (>0 and ≤50g/week). HRs are plotted against the mean usual alcohol consumption in each category. Studies with fewer than five events of any outcome were excluded from the analysis of that outcome. The sizes of the boxes are proportional to the inverse of the variance of the log-transformed hazard ratios. Vertical lines represent 95% CIs.

eFigure 9a: Hazard ratios per 100 grams/week higher usual alcohol consumption for subtypes of cardiovascular outcomes amongst current drinkers, additionally adjusted for body-mass index.

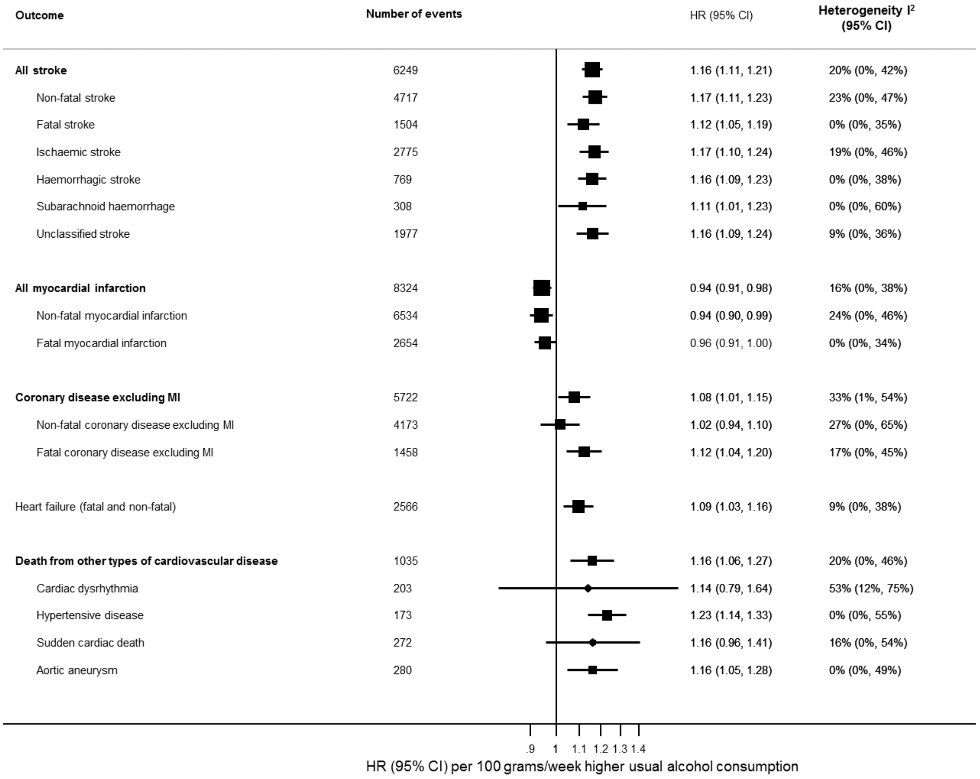

eFigure 9b. Shape of association of usual alcohol consumption with all-cause mortality and all cardiovascular disease amongst current drinkers, additionally adjusted for body-mass index.

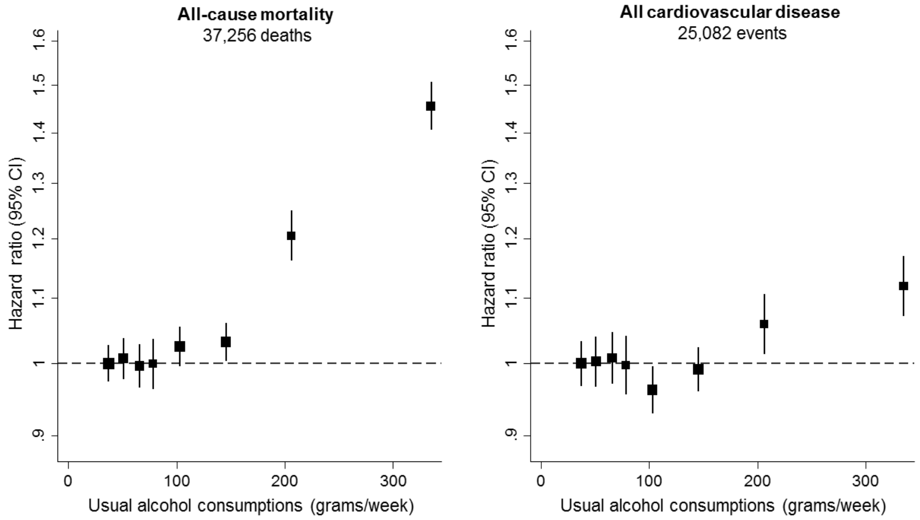

Adjusted for BMI, age, smoking and history of diabetes, and stratified by sex and EPIC centre. Alcohol consumption categories amongst current drinkers were >0-≤50 grams/week, >50-≤100 grams/week, >100-≤150 grams/week, >150-≤250 grams/week, >250-≤350 grams/week and >350 grams/week. The reference category is the lowest baseline alcohol consumption category (>0 and ≤50g/week). HRs are plotted against the mean usual alcohol consumption in each category. Studies with fewer than five events of any outcome were excluded from the analysis of that outcome. The sizes of the boxes are proportional to the inverse of the variance of the log-transformed hazard ratios. Vertical lines represent 95% CIs.

eFigure 10: Shape of association between baseline alcohol consumption, including ex- and non-drinkers, with all cardiovascular disease and all-cause mortality.

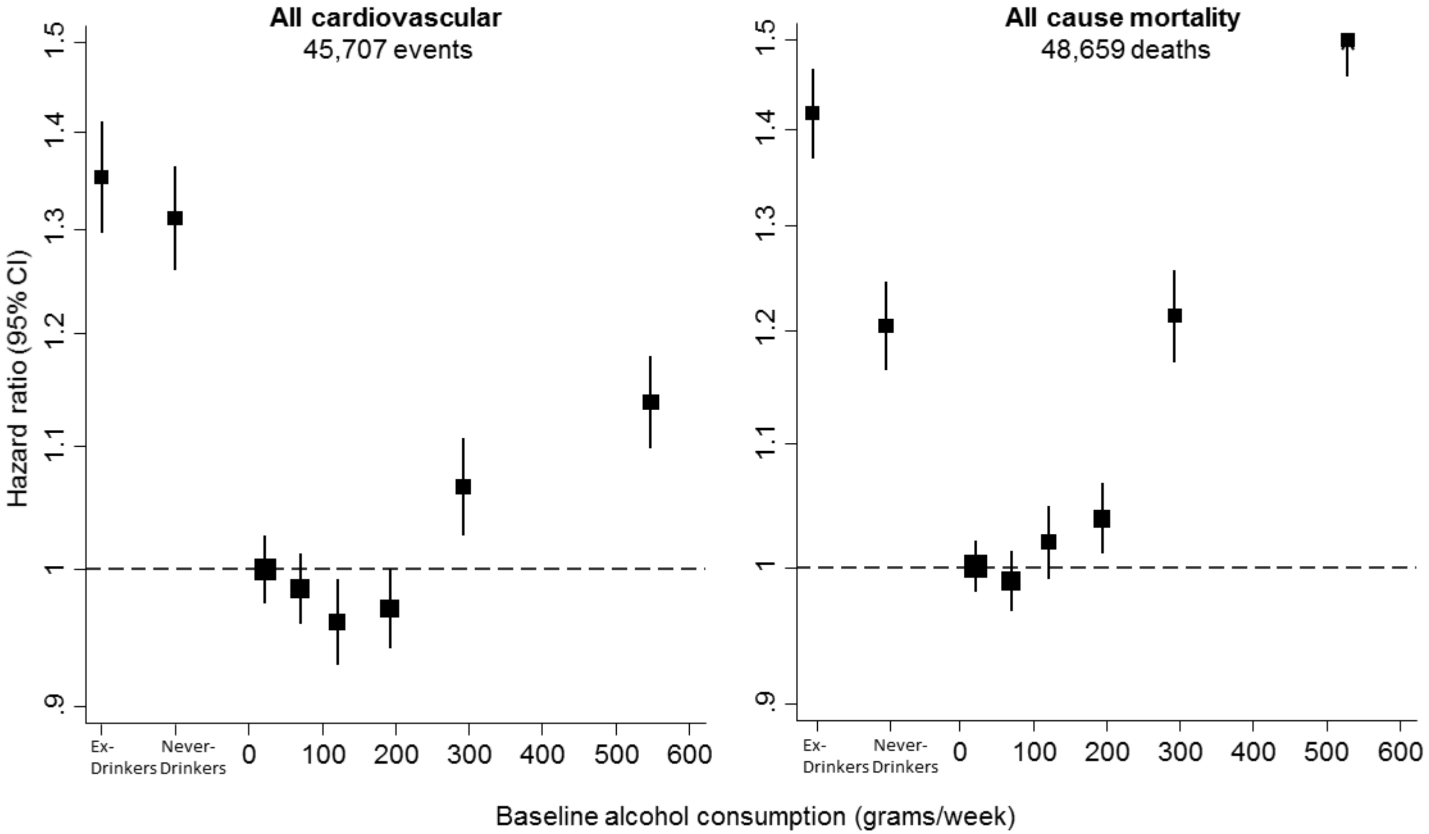

Adjusted for age, smoking and history of diabetes, and stratified by sex and EPIC centre. Alcohol consumption categories amongst current drinkers were >0-≤50 grams/week, >50-≤100 grams/week, >100-≤150 grams/week, >150-≤250 grams/week, >250-≤350 grams/week and >350 grams/week. The reference category is the lowest baseline alcohol consumption category (>0 and ≤50g/week). Studies with fewer than five events of any outcome were excluded from the analysis of that outcome. The sizes of the boxes are proportional to the inverse of the variance of the log-transformed hazard ratios. Vertical lines represent 95% CIs. Individuals for whom we were unable to distinguish as ex- or never- drinkers were excluded from the analysis.

eFigure 11: Hazard ratios per 100 grams/week higher baseline alcohol consumption for subtypes of cardiovascular outcomes amongst current drinkers with recorded baseline alcohol consumption (left) compared against all current drinkers using multiple imputation (right).

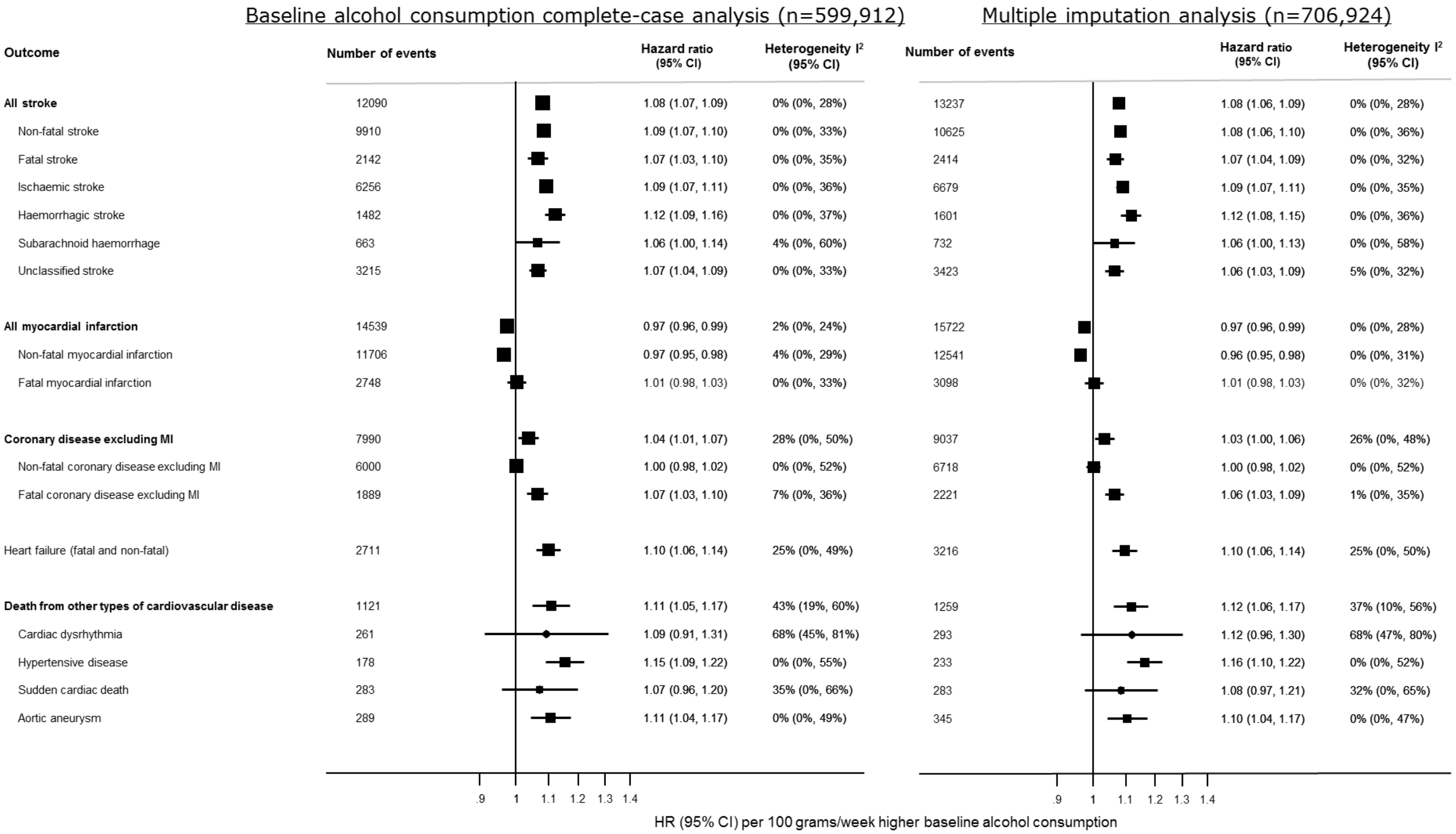

Missing alcohol consumption (log transformed) for known current drinkers was imputed using standard multiple imputation methods separately within each study, using known predictors for age, gender, smoking status, history of diabetes, indicators for all CVD disease categories listed in table above and their corresponding Nelson-Aalen estimators, weighted appropriately for the sampling fraction in EPIC-CVD (see White, I. R., Royston, P. and Wood, A. M. (2011), Multiple imputation using chained equations: Issues and guidance for practice. Statist. Med., 30: 377–399. doi:10.1002/sim.4067). Twenty imputed datasets were created for each study. The analysis was then performed separately by study, pooling imputation-specific estimates using Rubin's rules. This was followed by a random-effects meta-analysis.

eFigure 12: Shapes of associations of baseline alcohol consumption with stroke and coronary outcomes amongst alcohol drinkers

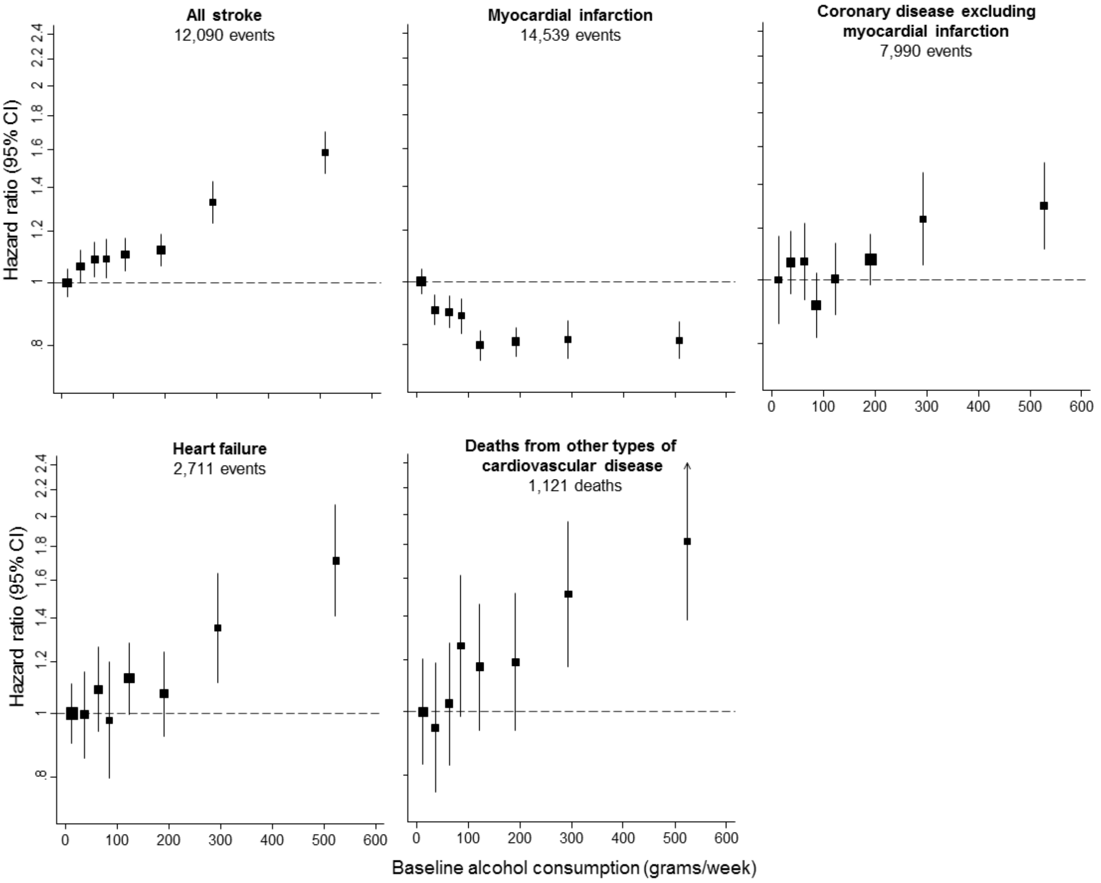

Adjusted for age, smoking and history of diabetes, and stratified by sex and EPIC centre. The reference category is the lowest baseline alcohol consumption category (>0 and ≤25g/week). HRs are plotted against the mean baseline alcohol consumption in each category. Studies with fewer than five events of any outcome were excluded from the analysis of that outcome. The sizes of the boxes are proportional to the inverse of the variance of the log-transformed hazard ratios. Vertical lines represent 95% CIs. The best-fitting fractional polynomial Cox models on the log scale were: all stroke, linear (ie, powers 1); myocardial infarction, log-linear (powers 0); coronary disease excluding myocardial infarction, linear (ie, powers 1); heart failure, linear (ie, powers 1); and deaths from other types of cardiovascular disease, linear (ie, powers 1).

eFigure 13: Best fitting second degree fractional polynomial for the modelled shape of association between baseline alcohol consumption with all-cause mortality.

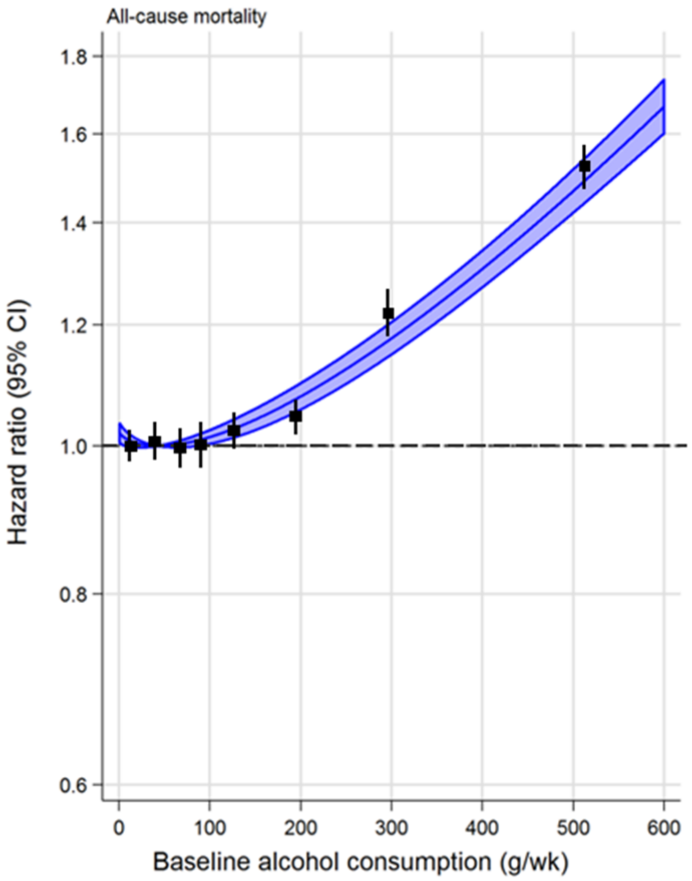

To estimate the alcohol consumption level at which mortality risk was lowest, we conducted nonlinear modelling by fitting a Cox regression model stratified by cohort, sex and trial arm (where applicable), to determine a best fitting second degree fractional polynomial model (FP2) for baseline alcohol consumption.

eFigure 14: Hazard ratios per 100 grams/week higher usual alcohol consumption for subtypes of cardiovascular outcomes amongst current drinkers from a fixed-effect meta-analysis.

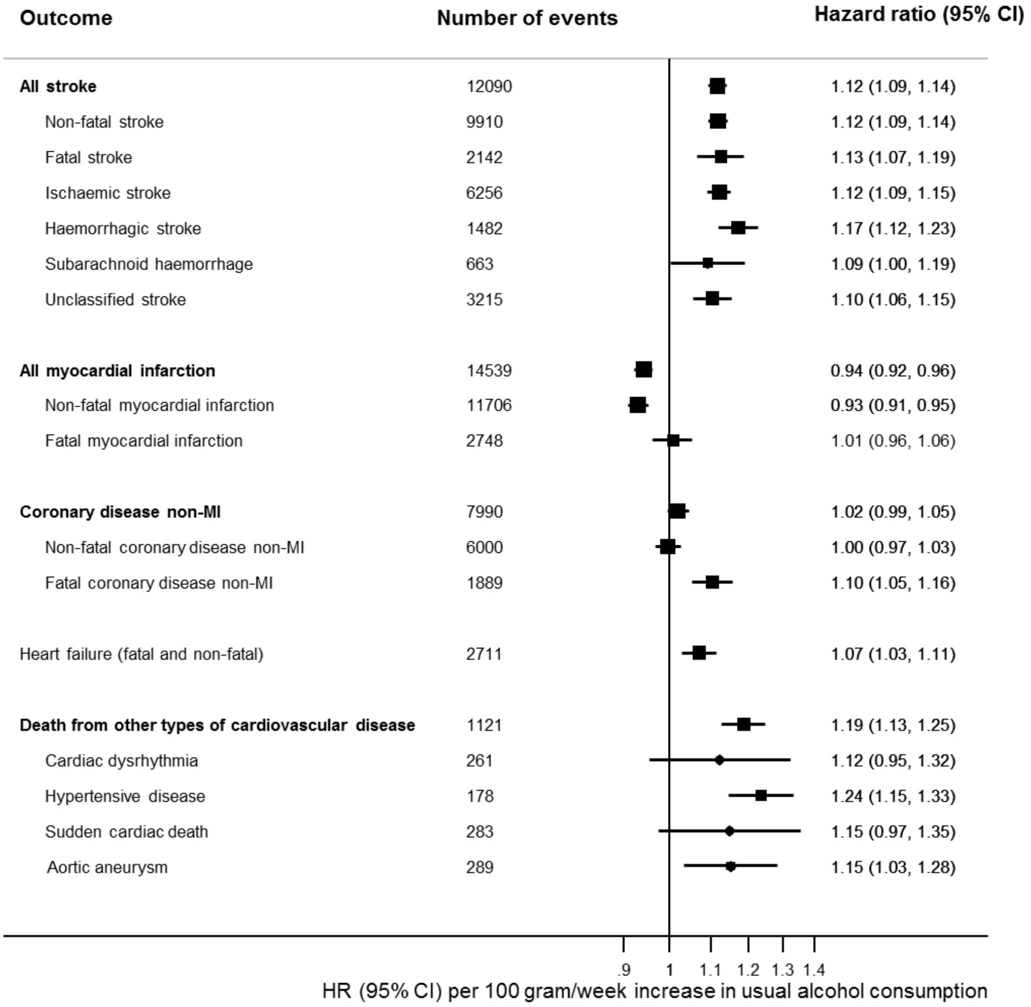

Adjusted for age, smoking and history of diabetes.
Studies of the same design (ie, prospective, case-cohort and nested case-control studies) were analysed together in a single model, stratified by cohort, sex and EPIC centre. Results from each study design were then combined in a fixed-effect meta-analysis. Studies with fewer than five events of any outcome were excluded from the analysis of that outcome.

eFigure 15: Hazard ratios per 100 grams/week higher usual alcohol consumption for subtypes of cardiovascular outcomes amongst current drinkers, from fixed-effect analysis with inclusion of studies with fewer than 5 outcomes of a particular type.

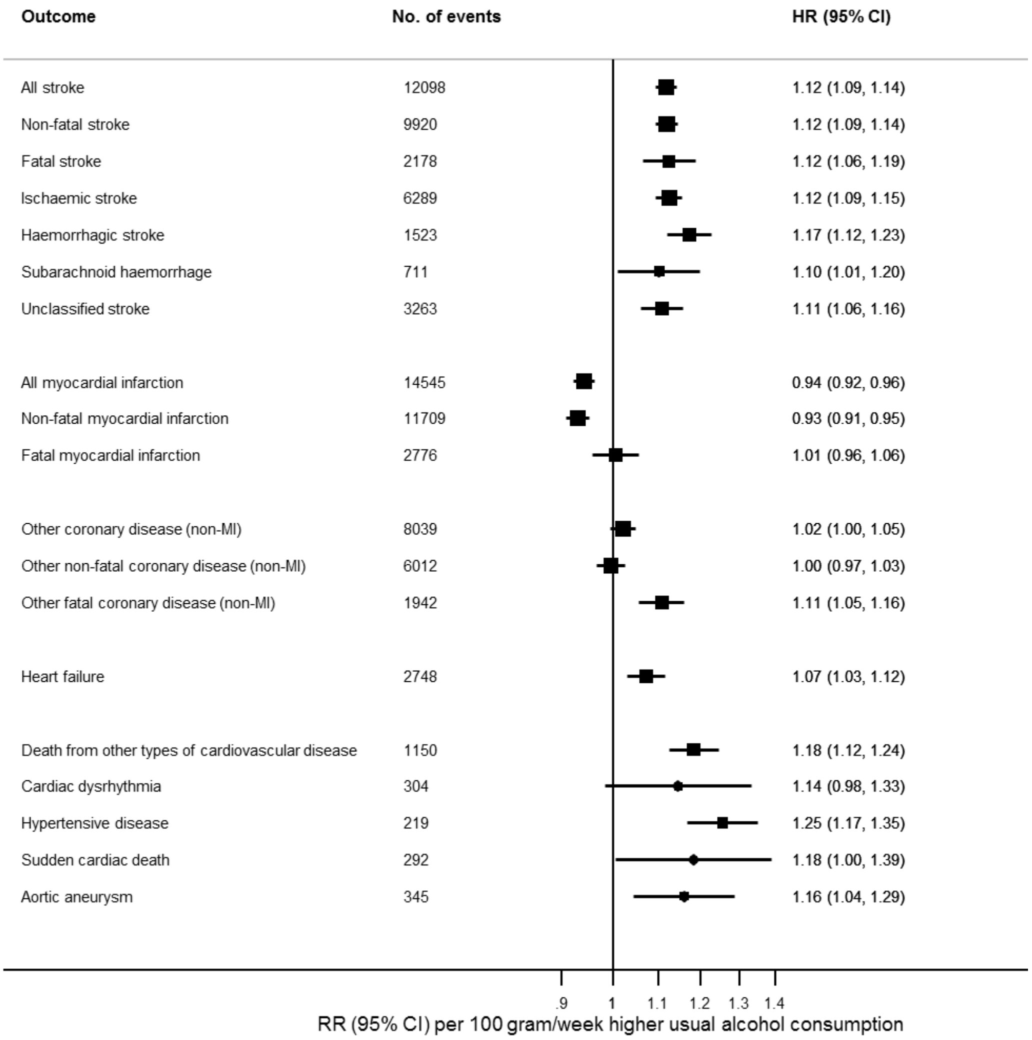

Adjusted for age, smoking and history of diabetes.
Studies of the same design (ie, prospective, case-cohort and nested case-control studies) were analysed together in a single model, stratified by cohort, sex and EPIC centre. Results from each study design were then combined in a fixed-effect meta-analysis. This analysis included all studies.

eFigure 16: Shapes of associations of usual alcohol consumption with stroke and coronary outcomes amongst current alcohol drinkers restricted to studies recording both fatal and non-fatal endpoints.

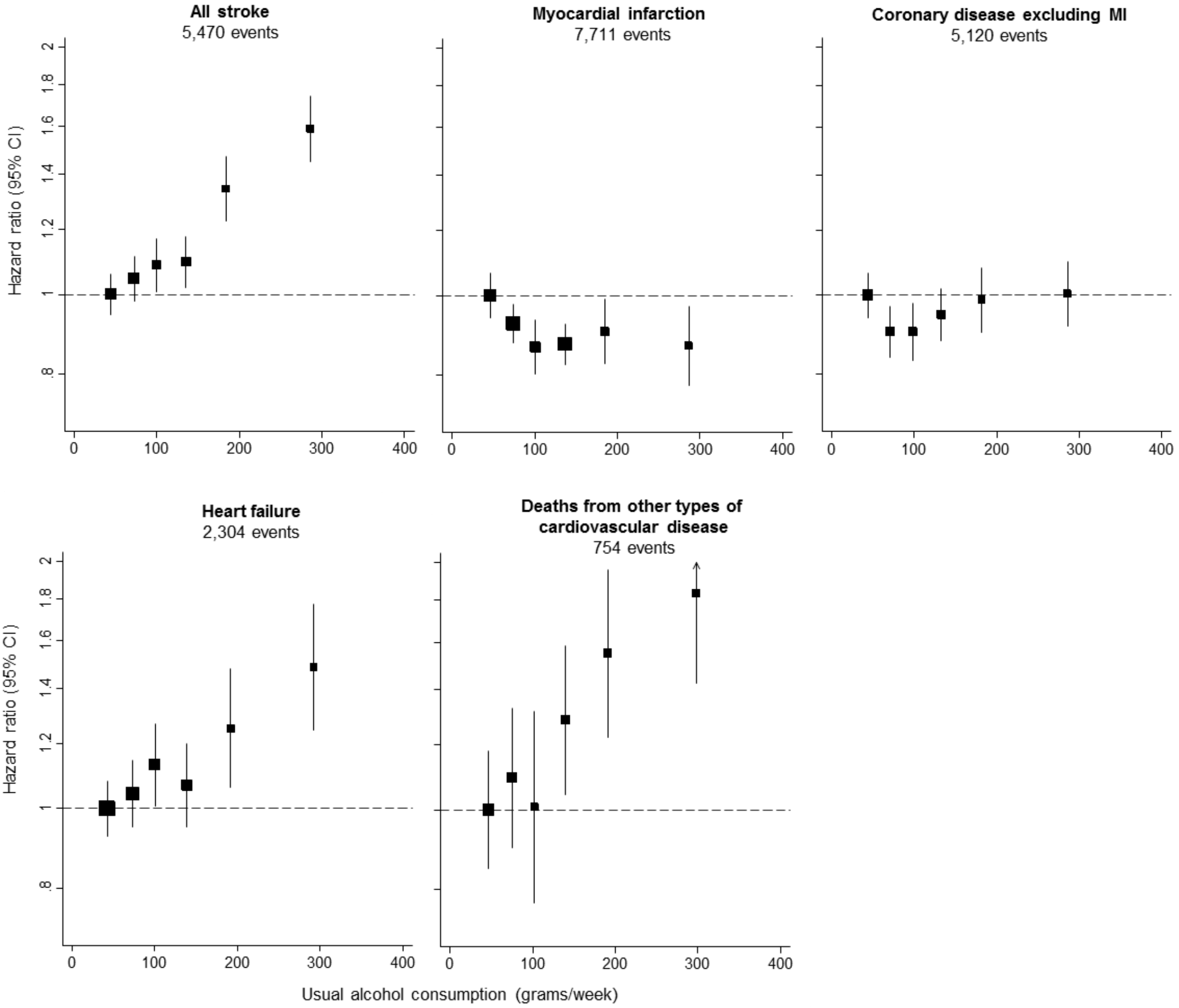

Analysis restricted to studies recording fatal and non-fatal cardiovascular diseases. Adjusted for age, smoking and history of diabetes, and stratified by sex and EPIC centre. Alcohol consumption categories amongst current drinkers were >0-≤50 grams/week, >50-≤100 grams/week, >100-≤150 grams/week, >150-≤250 grams/week, >250-≤350 grams/week and >350 grams/week. The reference category is the lowest baseline alcohol consumption category (>0 and ≤50g/week). HRs are plotted against the mean usual alcohol consumption in each category. Studies with fewer than five events of any outcome were excluded from the analysis of that outcome. The sizes of the boxes are proportional to the inverse of the variance of the log-transformed hazard ratios. Vertical lines represent 95% CIs.

eFigure 17: Shapes of associations of baseline alcohol consumption with all-cause mortality by (a) consumption frequency, (b) consumption type* and (c) binge drinking status.

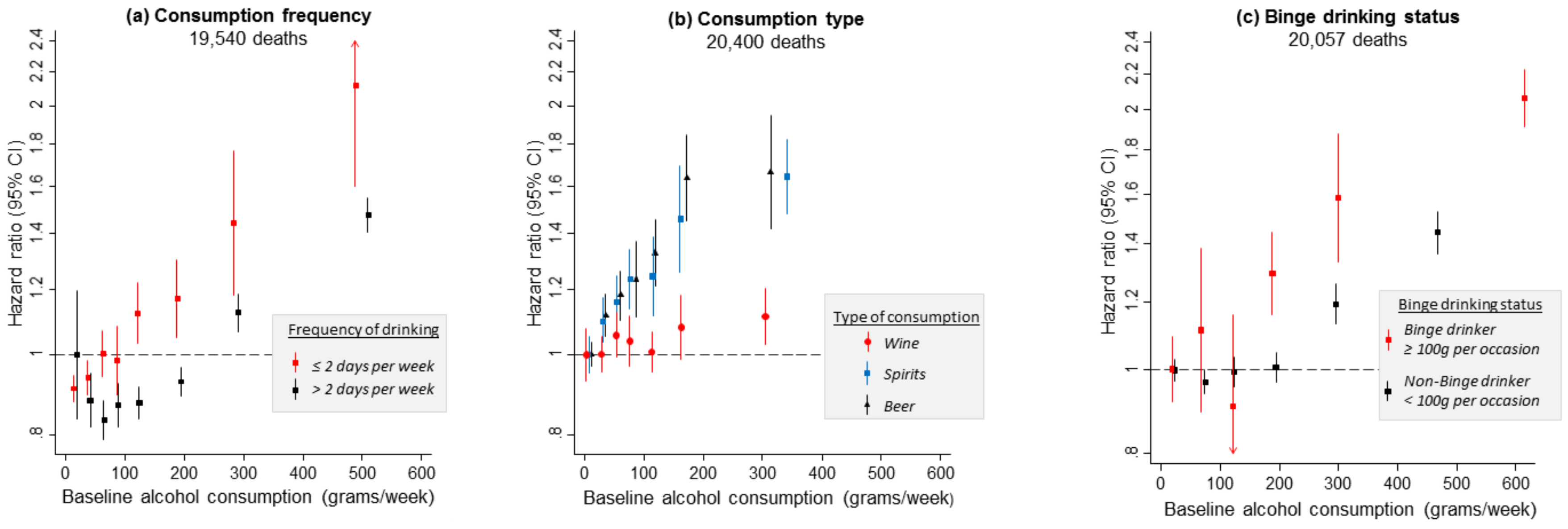

Adjusted for age, smoking and history of diabetes, and stratified by sex and EPIC centre.

*Analysis was performed separately for each alcohol consumption type (351,342 wine drinkers; 227,469 beer drinkers; 171,770 spirits drinkers). Individuals drinking more than one type of alcohol were included in each separate analysis.

eFigure 18. Hazard ratios per 100 grams/week higher baseline alcohol consumption for major cardiovascular outcomes amongst current drinkers and by alcohol type.

Outcome	Type of Alcohol consumption	No. of events	Hazard ratio (95% CI)	P-value[1]	P-value[2]	Heterogeneity I^2 (95% CI)
**All stroke**	All alcohol	8,269	1.08 (1.06, 1.09)			0% (0%, 41%)
	Wine		1.01 (0.95, 1.07)			20% (0%, 49%)
	Beer		1.11 (1.06, 1.16)	0.12		15% (0%, 46%)
	Spirits		1.22 (1.18, 1.26)	0.012	0.63	0% (0%, 41%)
**All myocardial infarction**	All alcohol	10,038	0.97 (0.96, 0.99)			0% (0%, 40%)
	Wine		0.93 (0.88, 0.98)			25% (0%, 52%)
	Beer		0.99 (0.97, 1.02)	0.15		0% (0%, 40%)
	Spirits		1.12 (1.07, 1.17)	<0.001	0.26	3% (0%, 31%)
**Coronary disease excluding MI**	All alcohol	5,791	1.00 (0.99, 1.02)			0% (0%, 48%)
	Wine		0.97 (0.92, 1.03)			16% (0%, 51%)
	Beer		1.02 (0.99, 1.04)	0.54		0% (0%, 48%)
	Spirits		1.15 (1.10, 1.20)	0.04	0.27	0% (0%, 48%)
**Heart failure**	All alcohol	1,241	1.09 (1.06, 1.13)			0% (0%, 51%)
	Wine		0.98 (0.85, 1.13)			34% (0%, 63%)
	Beer		1.13 (1.08, 1.18)	0.02		0% (0%, 51%)
	Spirits		1.16 (1.07, 1.25)	0.16	0.07	21% (0%, 56%)

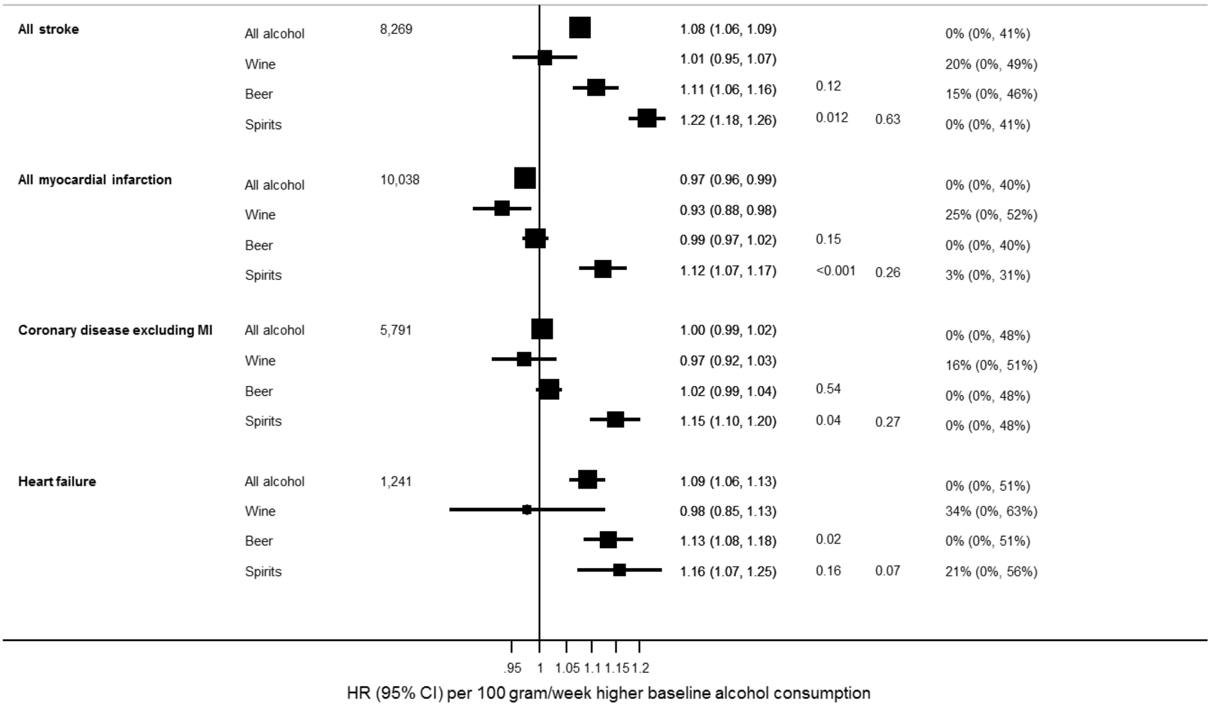

Analyses were restricted to 430,433 individuals with known alcohol type (351,342 wine drinkers; 227,469 beer drinkers; 171,770 spirits drinkers). MI: Myocardial infarction.

Adjusted for age, smoking and history of diabetes, and stratified by sex and EPIC centre. Indicator variables for consumption-type were also included in the models.
Studies with fewer than five events of any outcome were excluded from the analysis of that outcome.
P-value[1] for difference in hazard ratios for beer versus wine and spirits versus wine. Comparisons were restricted to beer and wine drinkers and spirits and wine drinkers respectively.
P-value[2] for difference in hazard ratios for beer versus spirits. Comparison was restricted to beer and spirits drinkers.

eFigure 19a-e: Hazard ratios per 100 gram/week increase in usual alcohol consumption for major vascular outcomes amongst current drinkers by study/cohort-level characteristics.

a. Stroke

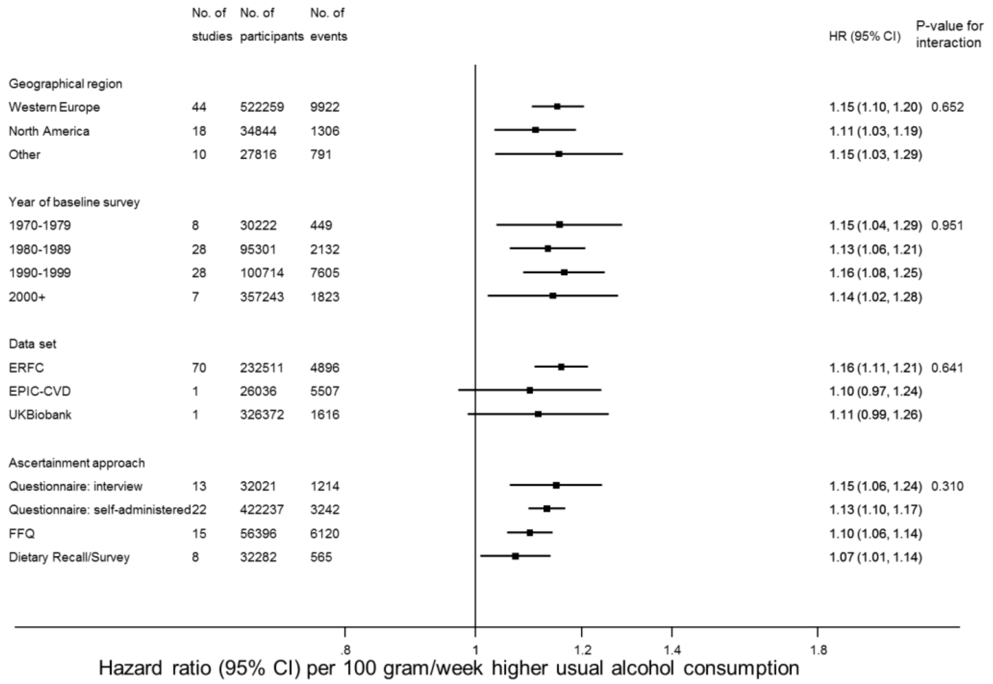

b. Myocardial infarction

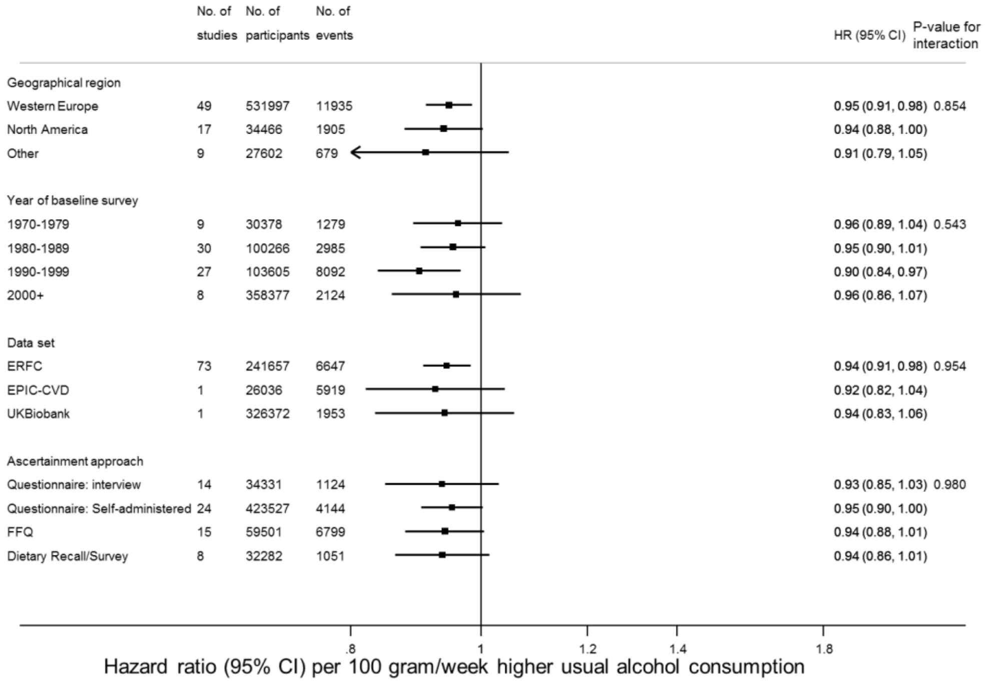

c. Coronary disease excluding myocardial infarction

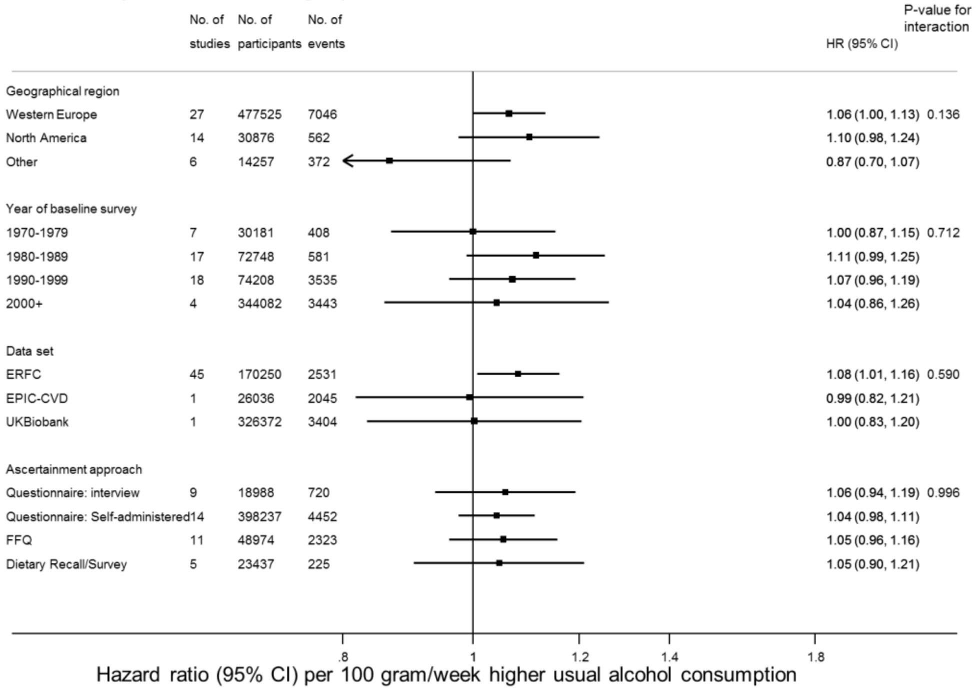

d. Heart failure

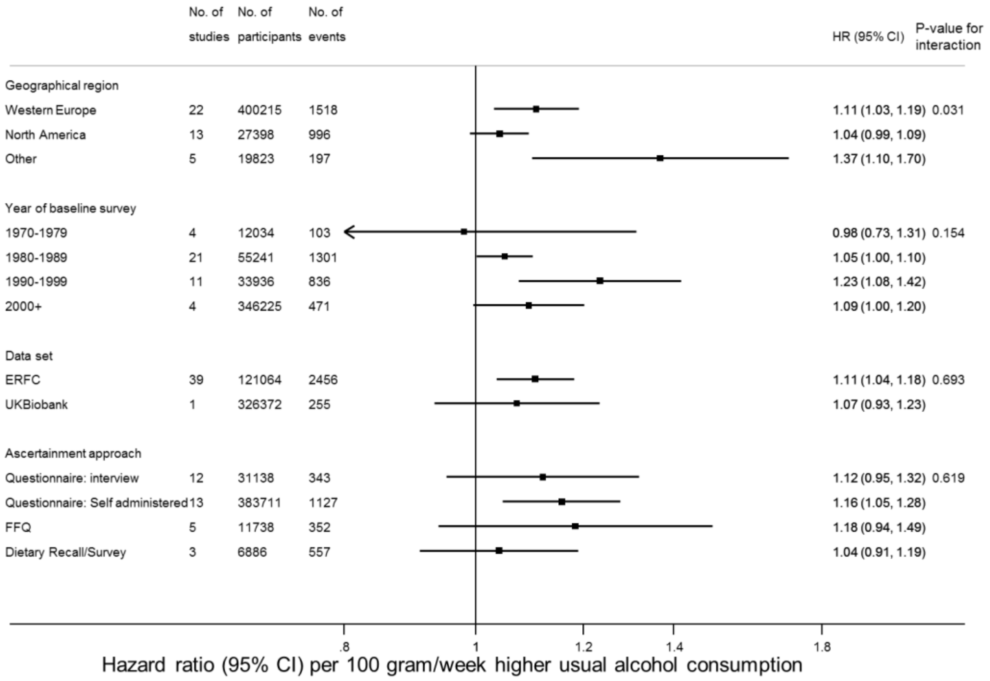

e. Deaths from other types of cardiovascular disease

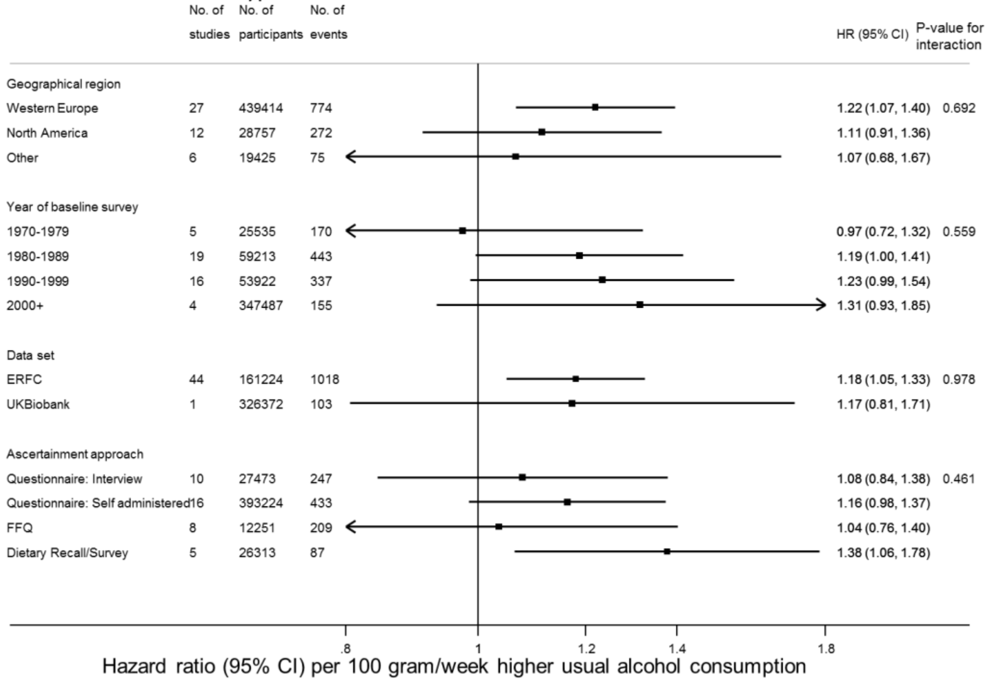

Adjusted for age, smoking and history of diabetes, and stratified by sex and EPIC centre. Studies with fewer than five events of any outcome were excluded from the analysis of that outcome. The sizes of the boxes are proportional to the inverse of the variance of the log-transformed hazard ratios.
Geographical region “other” included studies in Australia and New Zealand. Studies from Japan were exlcuded. The studies included in this analysis recruited participants over different calendar periods (ERFC: 1964-2008; EPIC-CVD: 1990-2002; UK Biobank: 2005-2014)

eFigure 20a-e: Hazard ratios per 100 gram/week higher usual alcohol consumption for major cardiovascular outcomes amongst current drinkers by individual-level characteristics.

a. Stroke

Characteristic	No. of studies	No. of events	HR (95% CI)	P-value for interaction
Sex				
Male	54	6194	1.16 (1.11, 1.21)	0.947
Female	54	4663	1.16 (1.09, 1.23)	
Age at survey (yrs)				
40-59	32	1084	1.24 (1.15, 1.33)	0.078
60-69	40	2193	1.21 (1.14, 1.29)	
70+	41	4256	1.13 (1.05, 1.22)	
Ethnicity				
White	17	2569	1.12 (1.07, 1.17)	0.979
Non-white	17	246	1.12 (1.02, 1.24)	
Smoking status				
Other	80	8206	1.15 (1.12, 1.18)	0.176
Current	79	3813	1.12 (1.09, 1.16)	
History of diabetes				
Other	70	11092	1.16 (1.12, 1.19)	0.585
Definite diabetic	70	910	1.18 (1.10, 1.27)	
BMI (kg/m2)				
Bottom tertile	68	3788	1.21 (1.15, 1.28)	0.031
Middle tertile	68	3926	1.14 (1.09, 1.19)	
Top tertile	68	4019	1.15 (1.09, 1.20)	
SBP (mmHg)				
Bottom tertile	70	2029	1.12 (1.07, 1.18)	0.012
Middle tertile	70	3165	1.06 (1.02, 1.11)	
Top tertile	70	6103	1.16 (1.12, 1.21)	
HDL-C (mmol/l)				
Bottom tertile	55	2746	1.20 (1.13, 1.28)	0.431
Middle tertile	55	2709	1.25 (1.17, 1.33)	
Top tertile	54	2522	1.20 (1.11, 1.30)	
Total physical activity index				
Inactive/ moderately inactive	1	2627	1.14 (1.08, 1.20)	0.503
Moderately active	1	1904	1.15 (1.07, 1.23)	
Active	1	385	1.23 (1.09, 1.39)	
Self-reported general health				
Bottom half	20	2252	1.19 (1.10, 1.29)	0.913
Top half	20	465	1.18 (1.07, 1.31)	

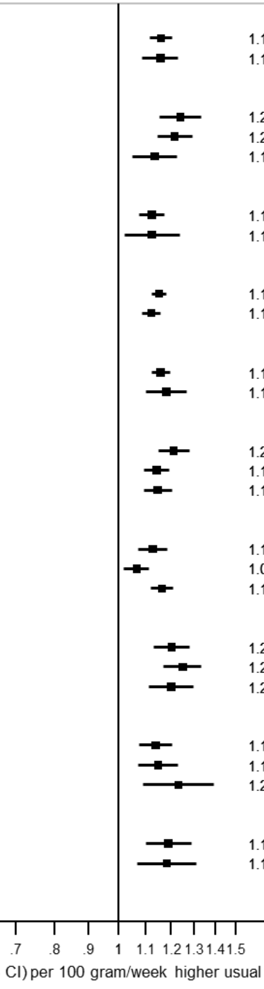

b. Myocardial infarction

Characteristic	No. of studies	No. of events	HR (95% CI)	P-value for interaction
Sex				
Male	54	8837	0.94 (0.90, 0.98)	0.944
Female	54	3384	0.95 (0.82, 1.09)	
Age at survey (yrs)				
40-59	33	1685	1.01 (0.93, 1.10)	0.064
60-70	41	3111	0.99 (0.91, 1.09)	
70+	41	3945	0.90 (0.82, 1.00)	
Ethnicity				
White	16	3718	0.95 (0.90, 1.00)	0.794
Non-white	16	226	0.93 (0.82, 1.06)	
Smoking status				
Other	83	8894	0.95 (0.92, 0.99)	0.160
Current	83	5625	0.92 (0.89, 0.96)	
History of diabetes				
Other	73	13367	0.96 (0.92, 1.00)	0.001
Definite diabetic	73	1081	0.83 (0.76, 0.90)	
BMI (kg/m2)				
Bottom tertile	71	3732	0.95 (0.90, 1.00)	0.704
Middle tertile	71	5018	0.97 (0.92, 1.03)	
Top tertile	71	5467	0.94 (0.89, 1.00)	
SBP (mmHg)				
Bottom tertile	73	2726	0.94 (0.88, 1.01)	0.661
Middle tertile	73	4256	0.93 (0.88, 0.99)	
Top tertile	73	6537	0.92 (0.86, 0.97)	
HDL-C (mmol/l)				
Bottom tertile	61	4487	0.99 (0.93, 1.04)	0.295
Middle tertile	61	3377	1.01 (0.93, 1.11)	
Top tertile	60	2047	1.04 (0.97, 1.12)	
Total physical activity index				
Inactive/ moderately inactive	1	2929	0.88 (0.83, 0.93)	0.460
Moderately active	1	1965	0.84 (0.78, 0.90)	
Active	1	397	0.90 (0.79, 1.03)	
Self-reported general health				
Bottom half	20	2520	0.95 (0.84, 1.07)	0.964
Top half	20	482	0.94 (0.83, 1.08)	

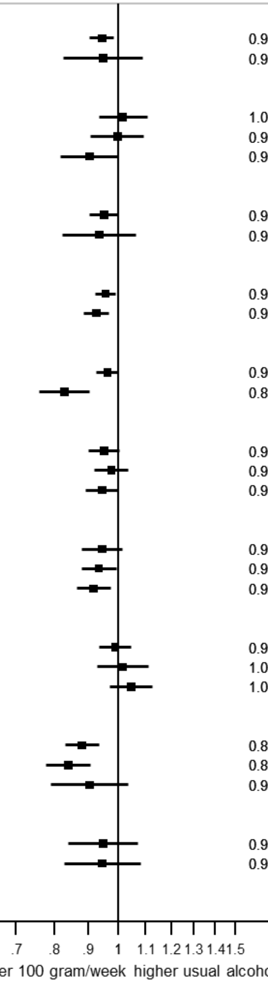

c. Coronary disease excluding myocardial infarction

Characteristic	No. of studies	No. of events	HR (95% CI)	P-value for interaction
Sex				
Male	32	4944	1.06 (0.99, 1.13)	0.285
Female	32	2354	1.18 (0.99, 1.41)	
Age at survey (yrs)				
40-59	23	876	1.15 (1.07, 1.24)	0.002
60-69	28	2176	1.05 (0.97, 1.15)	
70+	28	3799	1.00 (0.91, 1.10)	
Ethnicity				
White	14	3761	1.01 (0.95, 1.07)	0.009
Non-white	14	220	1.19 (1.06, 1.33)	
Smoking status				
Other	52	6016	1.07 (0.99, 1.16)	0.516
Current	52	1949	1.10 (1.03, 1.18)	
History of diabetes				
Other	47	7373	1.06 (1.00, 1.12)	0.716
Definite diabetic	47	607	1.04 (0.92, 1.17)	
BMI (kg/m2)				
Bottom tertile	42	1879	1.14 (1.06, 1.23)	0.010
Middle tertile	42	2668	1.05 (0.98, 1.12)	
Top tertile	42	3208	1.15 (1.07, 1.24)	
SBP (mmHg)				
Bottom tertile	46	1380	1.07 (0.97, 1.18)	0.727
Middle tertile	46	2420	1.06 (0.97, 1.16)	
Top tertile	46	3989	1.09 (0.97, 1.22)	
HDL-C (mmol/l)				
Bottom tertile	36	1385	1.14 (1.01, 1.27)	0.046
Middle tertile	36	1291	1.30 (1.13, 1.49)	
Top tertile	35	932	1.17 (1.06, 1.29)	
Total physical activity index				
Inactive/Moderately active	1	876	1.06 (0.95, 1.17)	0.014
Moderately active	1	963	0.86 (0.75, 0.98)	
Active	1	167	1.14 (0.91, 1.41)	
Self-reported general health				
Bottom half	13	3775	1.05 (0.96, 1.15)	0.479
Top half	13	620	1.08 (0.97, 1.21)	

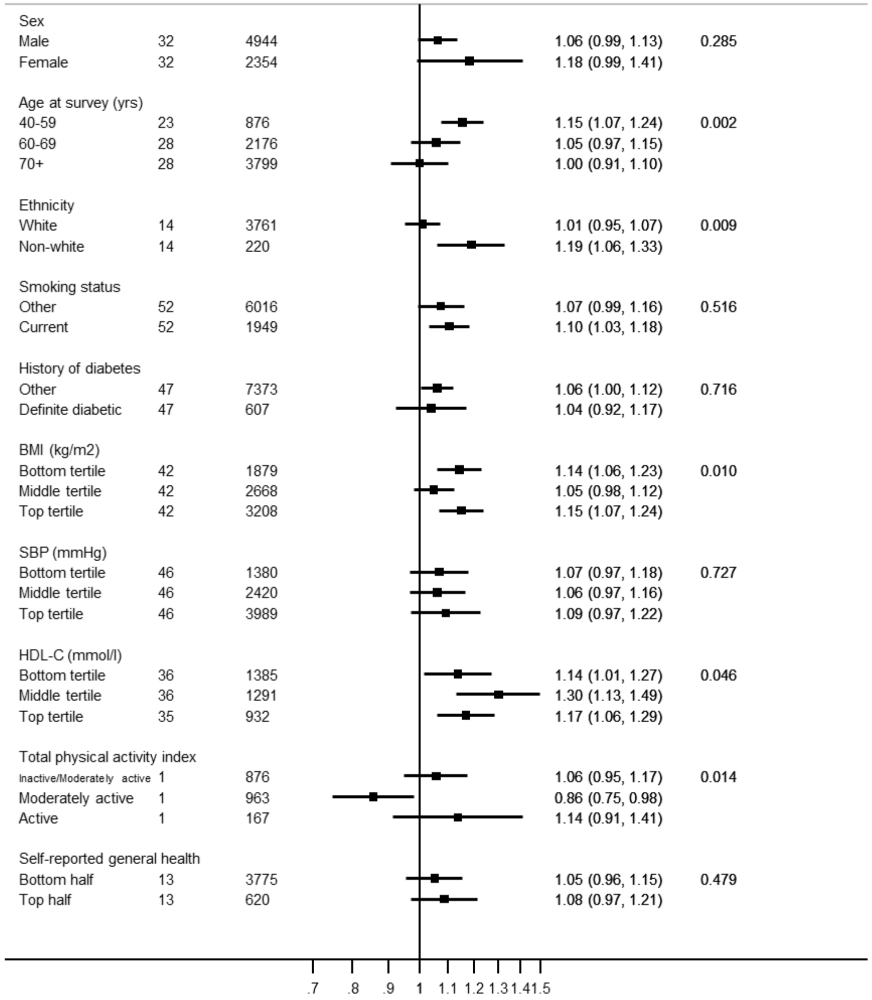

d. Heart failure

Characteristic	No. of studies	No. of events	HR (95% CI)	P-value for interaction
Sex				
Male	33	1411	1.13 (1.06, 1.21)	0.401
Female	33	1031	1.07 (0.94, 1.22)	
Age at survey (yrs)				
40-59	15	171	1.17 (1.02, 1.35)	0.011
60-69	20	504	1.30 (1.10, 1.54)	
70+	20	845	1.11 (0.95, 1.29)	
Ethnicity				
White	10	1028	1.04 (0.96, 1.13)	0.268
Non-white	10	166	1.10 (0.99, 1.22)	
Smoking status				
Other	40	1937	1.18 (1.08, 1.29)	0.766
Current	40	774	1.20 (1.09, 1.31)	
History of diabetes				
Other	39	2357	1.13 (1.06, 1.21)	0.572
Definite diabetic	39	346	1.18 (1.01, 1.37)	
BMI (kg/m2)				
Bottom tertile	36	666	1.17 (1.07, 1.28)	0.355
Middle tertile	36	741	1.15 (1.04, 1.27)	
Top tertile	36	1159	1.10 (1.00, 1.20)	
SBP (mmHg)				
Bottom tertile	38	617	1.07 (0.97, 1.19)	0.587
Middle tertile	38	761	1.11 (1.01, 1.21)	
Top tertile	38	1282	1.15 (1.06, 1.25)	
HDL-C (mmol/l)				
Bottom tertile	29	682	1.18 (1.03, 1.36)	0.430
Middle tertile	29	609	1.23 (1.08, 1.41)	
Top tertile	28	595	1.15 (0.99, 1.33)	
Self-reported general health				
Bottom half	15	820	1.15 (1.04, 1.28)	0.254
Top half	15	249	1.27 (1.06, 1.52)	

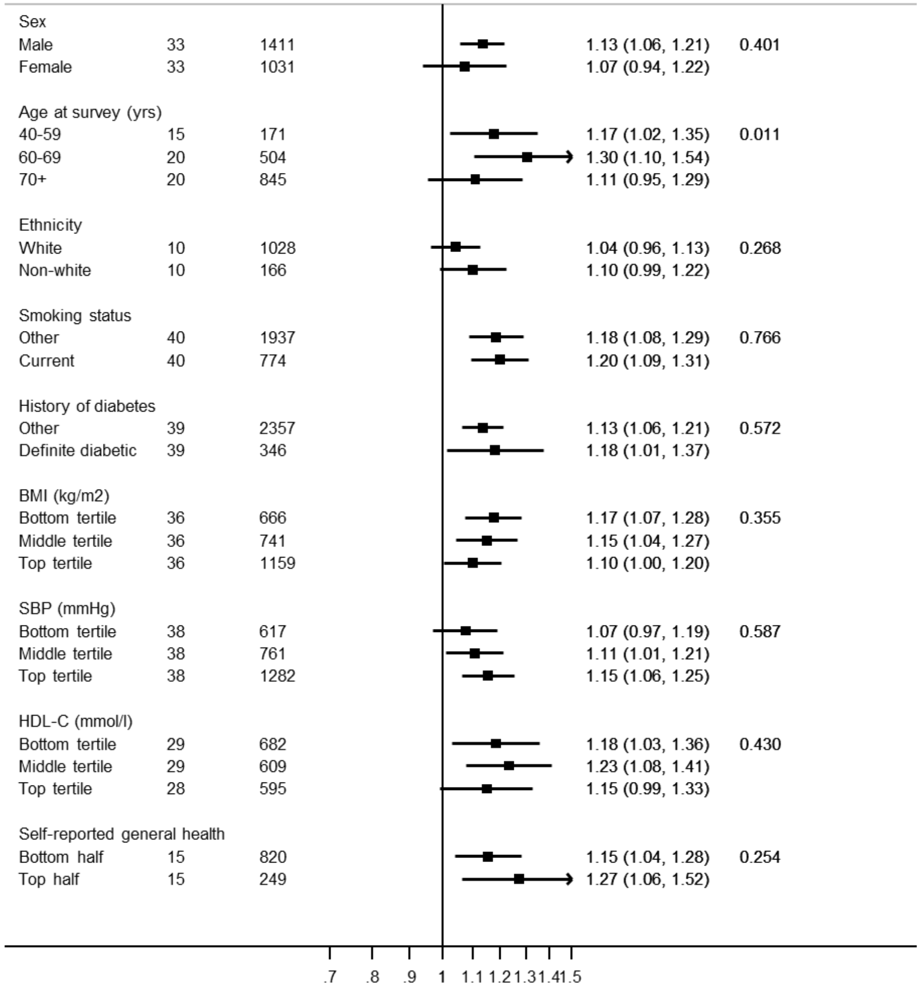

e. Deaths from other types of cardiovascular disease

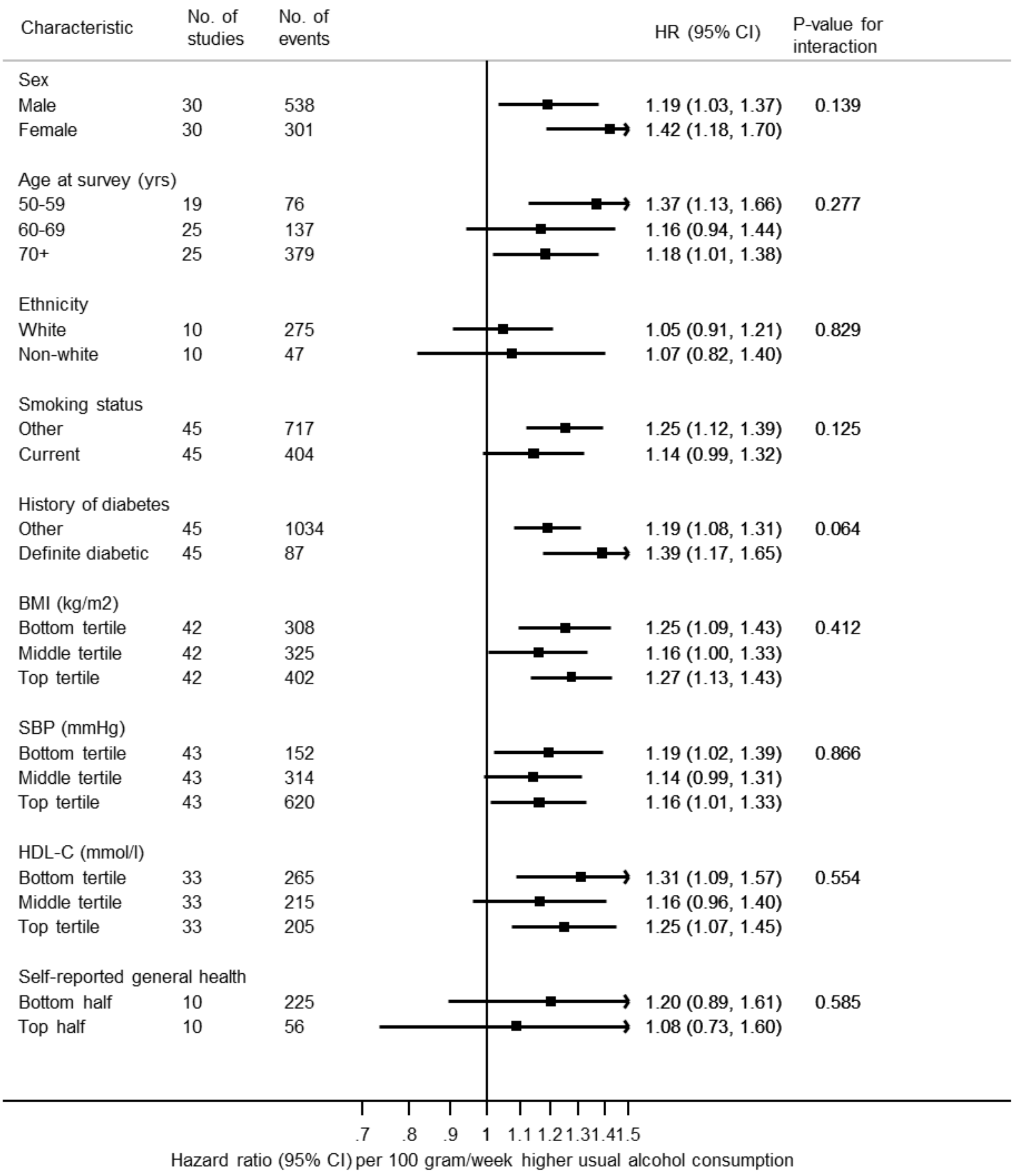

HRs were adjusted for age, smoking and history of diabetes and stratified by EPIC centre. BMI = body mass index; bottom third <24.10 kg/m^2, middle third 24.10-27.18 kg/m^2, top third >27.18kg/m^2. SBP = systolic blood pressure; bottom third <123 mmHg, middle third 123-141mmHg, top third>141mmHg. HDL-c bottom third <1.10 mmol/l, middle third 1.19-1.51mmol/l and top third >1.51 mmol/l. Self-reported general health[0-1] bottom half <0.67, top half>=0.67.

eFigure 21. Funnel plots and assessment of small-study effects for study-specfic hazard ratios per 100 gram/week increase in usual alcohol consumption for major vascular outcomes amongst current drinkers.

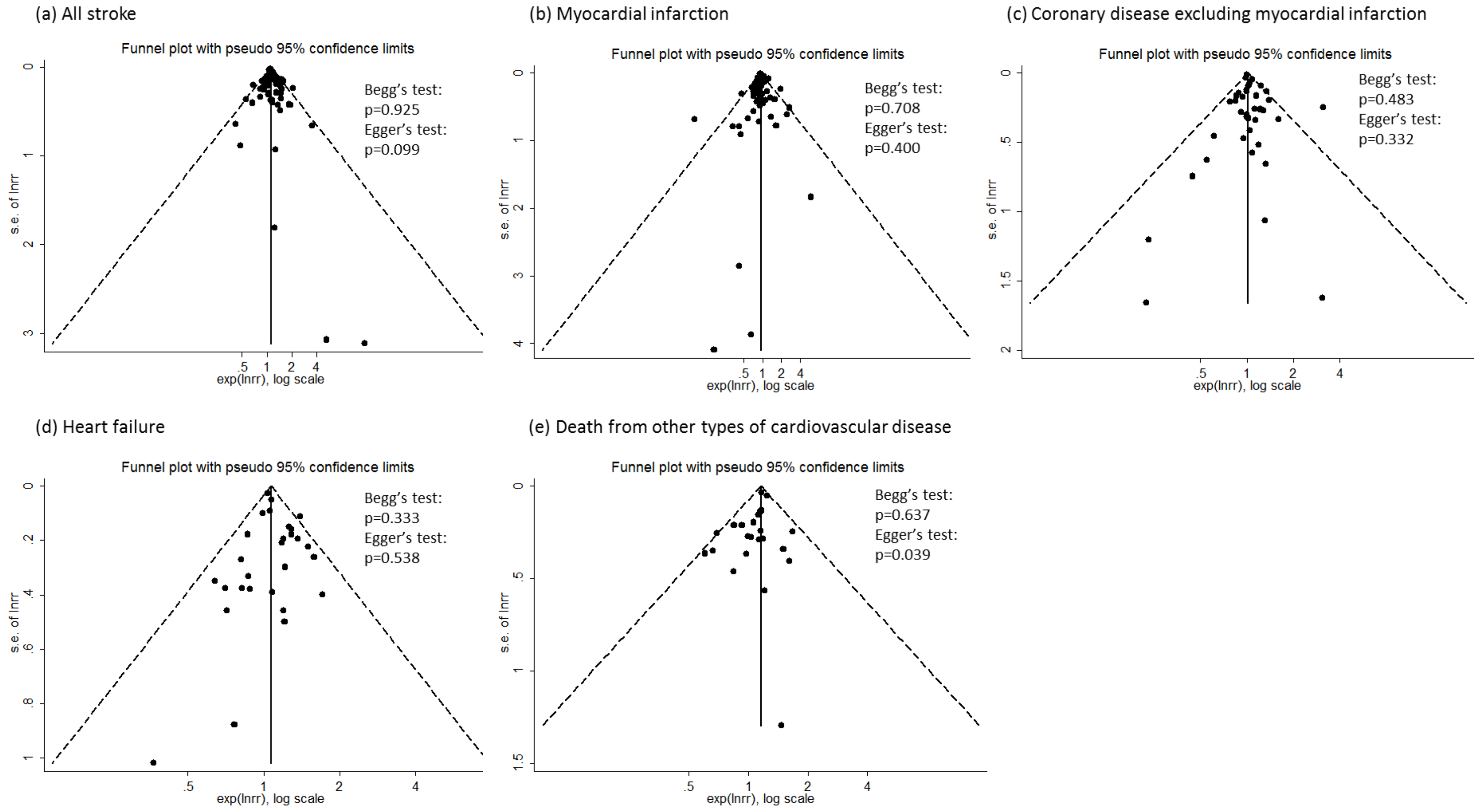

eFigure 22. Estimated future years of life lost in individuals reporting drinking above a range of hypothetical alcohol consumption thresholds compared to those reporting drinking less than the hypothetical alcohol consumption thresholds.

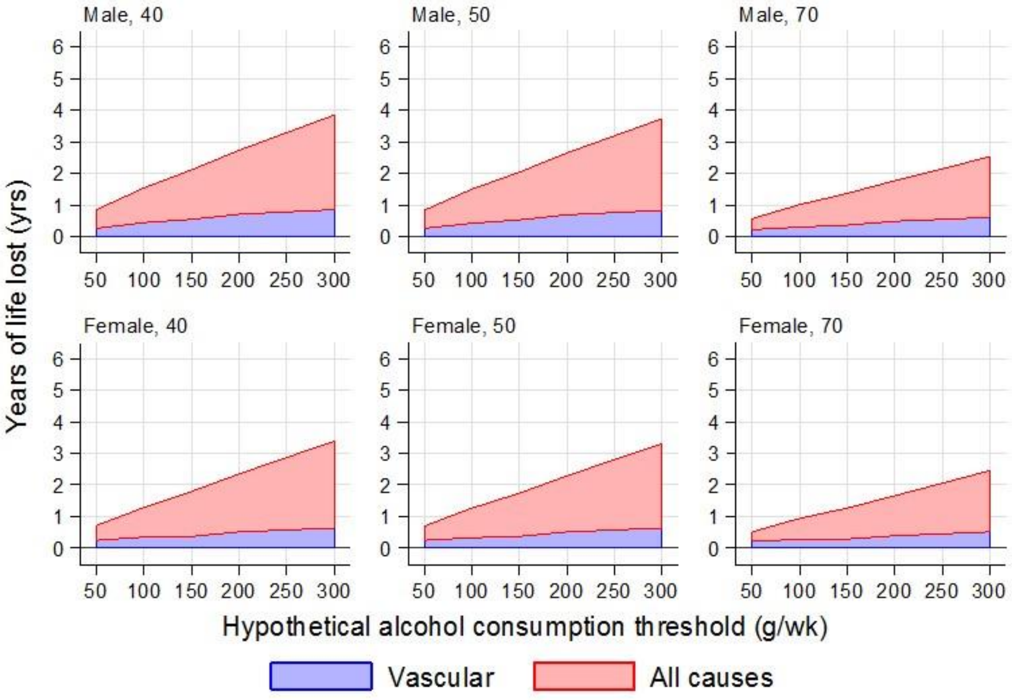

Interpretation: Males who reported drinking above 196 g/wk threshold have approximately 2.7 years (95% CI: 2.4-3.1) lower life expectancy at age 40 years than those who reported drinking below 196 g/wk. Similarly, males who reported drinking above 112 g/wk threshold have approximately 1.6 years (95% CI: 1.3-1.8) lower life expectancy at 40 years than those who reported drinking below 112 g/wk.

The estimates of cumulative survival from 40 years of age onward among the drinking groups were calculated by applying hazard ratios (specific to age at risk) for all-cause mortality associated with baseline alcohol consumption to US death rates at the age of 40 years or older.

Annex 6. Emerging Risk Factors Collaboration Investigators

Air Force/Texas Coronary Artherosclerosis Prevention Study (AFTCAPS): Robert W Tipping; Artherosclerosis Risk in Communities Study (ARIC): David Couper, Elizabeth Selvin, Pamela Lutsey; Cohort of Progetto CUORE (ATENA, MATISS83, 87 & 93): Chiara Donfrancesco, Luigi Palmieri, Simona Giampaoli; ATTICA Study (ATTICA): Christina Chrysohoou, Christos Pitsavos, Dimitrios Tousoulis; Australian Diabetes, Obesity, and Lifestyle Study (AUSDIAB): Dianna J Magliano, Jonathan E Shaw, Paul Z Zimmet; Busselton Health Study (BHS): Matthew W Knuiman; Bruneck Study (BRUN): Johann Willeit, Marlene Notdurfter, Siegfried Weger; British Women's Health and Heart Study (BWHHS): Antoinette Amuzu, Caroline E Dale, Juan P Casas; Caerphilly Prospective Study (CAPS): Yoav Ben-Shlomo; Cardiovascular Study in the Elderly (CASTEL): Edoardo Casiglia, Valérie Tikhonoff; Charleston Health Study (CHARL): Susan E Sutherland; Cardiovascular Health Study (CHS): Bruce M Psaty, Mary Cushman; Copenhagen City Heart Study (COPEN): Anne Tybjærg-Hansen, Janne S Tolstrup, Morten Grønbaek; Data from an Epidemiological Study on the Insulin Resistance Syndrome (DESIR): Beverley Balkau, Fabrice Bonnet, Michel Marre; Diet and Risk of Cardiovascular Disease in Spain (DRECE): David Lora Pablos, Miguel Menendez Orenga, Pilar Cancelas Navia; Dubbo Study of the Elderly (DUBBO): John McCallum, Yechiel Friedlander; Edinburgh Artery Study (EAS): Jackie Price, Stela Mclachlan; The Established Populations for the Epidemiologic Study of the Elderly Studies, Boston (EPESEBOS): James O Taylor; The Established Populations for the Epidemiologic Study of the Elderly Studies, Iowa (EPESEIOW): Robert Wallace; The Established Populations for the Epidemiologic Study of the Elderly Studies, New Haven (EPESENHA): Harlan Krumholz; The Established Populations for the Epidemiologic Study of the Elderly Studies, North Carolina (EPSENCA): Dan Blazer; Epidemiologische Studie zu Chancen der Verhütung und optimierten Therapie chronischer Erkrankungen in der älteren Bevölkerung (ESTHER): Ben Schöttker, Bernd Holleczek, Kai-Uwe Saum; Finrisk Cohort 1992 and 1997 (FINRISK92 FINRISK97): Erkki Vartiainen, Kennet Harald, Pekka Jousilahti; Fletcher Challenge Blood Study (FLETCHER): Mark Woodward; Funagata Study (FUNAGATA): Takamasa Kayama, Takeo Kato, Toshihide Oizumi; Golstrup Study (GOLSTRUP): Else Marie Bladbjerg, Jørgen Jespersen; Cohorts of the Risk Factors and Life Expectancy Pooling Project (GREPCO, MICOL, and NHR): Maurizio Trevisan; Helsinki Businessmen Study (HBS): Timo Strandberg; Hertfordshire Cohort Study (HCS): Cyrus Cooper, Elaine Dennison, Karen Jameson; Health in Men Study (HIMS): Graeme J Hankey, Leon Flicker, Osvaldo P Almeida; Hisayama Study (HISAYAMA): Toshiharu Ninomiya, Yasufumi Doi, Yutaka Kiyohara; Cohorts of CONOR (HUBRO, OPPHED, FINNMARK, and TROMS): Anne Johanne Søgaard, Inger Ariansen; Ikawa, Kyowa, and Noichi Study (IKNS) and Osaka Study (OSAKA): Akihiko Kitamura, Hiroyasu Iso, Kazumasa Yamagishi; North Karelia Project (KARELIA): Veikko Salomaa; Kuopio Ischaemic Heart Disease Study (KIHD): Jukka T Salonen, Kurl Sudhir, Tomi-Pekka Tuomainen; Longitudinal Aging Study Amsterdam (LASA): Hannie C Comijs, Renate de Jongh; Monitoring of CVD Risk Factors Project/Monitoring Project on Chronic Disease Risk Factors (MCVDRFP/MORGEN): Anneke Blokstra, Jet HA Smit, W M Monique Verschuren; MESA: Ralph Sacco, Robyn McClelland; MONICA/KORA Augsburg Surveys S1, S2, and S3 (MONICA_KORA1, MONICA_KORA2, MONICA_KORA3): Christa Meisinger, Wolfgang Koenig; MRC Study of Older People (MRCOLD): Astrid Fletcher; Multiple Risk Factor Intervention Trial 1 (MRFIT): Lewis H Kuller; First and Third National Health and Nutrition Examination Surveys (NHANES1, NHANES III): Richard F Gillum; Northwick Park Heart Study II (NPHSII): Jackie A Cooper; Nova Scotia Heart Survey (NSHS: Jonathan Shaffer, Joseph E Schwartz, Susan Kirkland; Oslo Study (OSLO2): Anne-Lise Lund Håheim; Puerto Rico Heart Health Program (PRHHP): Carlos J Crespo; Prospective Epidemiological Study of Myocardial Infarction (PRIME): Dominique Arveiler, Jean Ferrières, Michèle Montaye; Prospective Cardiovascular Münster Study (PROCAM): Gerd Assmann, Helmut Schulte; Prospective Study of Pravastatin in the Elderly at Risk (PROSPER): Stella Trompet, Wouter Jukema; Quebec Cardiovascular Study (QUEBEC): Benoît Lamarche, Bernard Cantin, Gilles R Dagenais; Rancho Bernardo Study (RANCHO): Deborah Wingard, Gail Laughlin, Kay Tee Khaw; The Rotterdam Study I (RS-1): Arfan Ikram, Frank van Rooij, Kim V E Braun; The Rotterdam Study II (RS-2): Ester A L de Jonge, Jessica Kiefte-de Jong, Maryam Kavousi; The Rotterdam Study III (RS-3): Henning Tiemeier, Taulant Muka, Zhangling Chen; Scottish Heart Health Extended Cohort (SHHEC): Hugh Tunstall-Pedoe; Toyama Study (TOYAMA): Hideaki Nakagawa, Masao Ishizaki, Yuko Morikawa; Uppsala Longitudinal Study of Adult Men (ULSAM): Martin Ingelsson, Vilmantas Giedraitis; Württemberg Construction Workers Cohort (WCWC): Dietrich Rothenbacher, Heiner Claessen, Hermann Brenner; Women's Health Initiative (Hormones and Biomarkers Predicting Stroke in Women; WHIHABPS): Amanda Fretts, Stacey Jolly, William James Howard; Whitehall I Study (WHITEI): Martin Shipley, Mika Kivimaki; Whitehall II Study (WHITEII): Eric J Brunner, Martin Shipley; West of Scotland Coronary Prevention Study (WOSCOPS): Michele Robertson, Naveed Sattar; Zutphen Elderly Study (ZUTE): Edith Feskens, Marianne Geleijnse, Daan Kromhout.
